# Molecular markers, mechanisms and metrics of biological aging: a scoping review

**DOI:** 10.3389/fragi.2026.1682867

**Published:** 2026-09-08

**Authors:** Alison Ziesel, Jennifer Reeves, Anastasia Mallidou, Lorelei Newton, Ryan E. Rhodes, Jie Zhang, Theone Paterson, Hosna Jabbari

**Affiliations:** 1 Department of Biomedical Engineering, University of Alberta, Edmonton, AB, Canada; 2 Department of Psychology, University of Victoria, Victoria, BC, Canada; 3 School of Nursing, University of Victoria, Victoria, BC, Canada; 4 Institute on Aging and Lifelong Health, University of Victoria, Victoria, BC, Canada; 5 School of Exercise Science, Physical and Health Education, University of Victoria, Victoria, BC, Canada; 6 Gustavson School of Business, University of Victoria, Victoria, BC, Canada

**Keywords:** biological age, biological clocks, blood-based biomarkers, metabolites, molecular biomarkers

## Abstract

Biological aging is a rapidly growing area of research, which entails characterizing the rate of aging independent of an individual’s chronological age. In this scoping review, we analyze the results of biological aging research in 435 papers published in a 12 year window spanning 2011 through June 2023, 309 of which focus on non-methylation-based biomarkers, revealing changing patterns of molecular markers of biological aging use over time as well as the development of novel metrics of biological aging. We further identify consistent and discordant research findings, as well as areas of potential future research focusing on questions of measurement with biomarker-based assessment and other variables relevant to the study of biological age.

## 1 Introduction

Biological aging is the process by which organisms develop, mature, and age, in parallel with but not exclusively dependent upon chronological age. Consequently, developmental time points and age-related disease risk may not be accurately predicted solely on chronological age. As an organism matures, the difference between chronological and biological age may widen, meaning that later in life the biological age differential can be significant. For these reasons it is important to be able to assess the progress of biological aging to precisely determine intervals of developmental change and disease vulnerability.

Biological aging is characterized by changes from the subcellular to the organismal level, and may be observed both microscopically and individually. These measurable features may indicate an individual entering a relatively advanced life stage, where they are prematurely more susceptible to of age-related conditions. Assessing individuals for biological aging changes may allow for intervention before disease onset, making accurate measurement of biological age critical for identifying windows for therapeutic and preventative measures to promote continuing good health.

The questions of what biological age represents, and how biological aging occurs, have been investigated for decades. Initially characterized as ‘functional age’ during the 1950s (McFarland, 1958), biological age resurged as a serious inquiry in the 1960s and 1970s (Dirken, 1972; Furukawa et al., 1975), although there was some debate as to its utility (Costa and McCrae, 1980; Dean and Morgan, 1988). Clinical parameters, blood- or tissue-derived protein or metabolite levels were often employed in early studies and their use has persisted. The early 1990s heralded the observation that telomere length in cells lacking telomerase decrease with progressive cell cycles, suggesting that it may be a usable proxy for cellular aging (Levy et al., 1992). In the following decade it was discovered that mitochondrial DNA (mtDNA) levels decline with age in skeletal muscle and other tissues (Welle et al., 2003; Cree et al., 2008). Changes in global and site-specific methylation were also observed, with work in this field conducted as early as 1967 (Berdyshev et al., 1967; Romanov and Vanyushin, 1981; Fernandez et al., 2012; Bocklandt et al., 2011), with the first, second and third generation epigenetic clocks following in later decades (Hannum et al., 2013; Horvath, 2013; Belsky et al., 2015; Levine et al., 2018; Lu et al., 2019; Belsky DW et al., 2020).

The preceding represents a high-level overview of decades of work and cannot adequately describe the discoveries and novel strategies developed over those years. While there exist reviews that examine detailed subsets of this work, we did not identify a comprehensive review considering all molecular biological features of biological aging, with the algorithms developed to interpret those data. We have prepared two reviews: this work focusing on molecular (non-methylation-based) features and algorithms, and a second review considering all methylation-based approaches to the calculation of biological age. These reviews address a critical gap in the literature by unifying a fragmented field—one where most studies focus narrowly on specific markers or algorithms—into a comprehensive synthesis of the molecular features of biological aging and related ‘clocks’. By examining research from the 12 year period spanning January 2011 through June 2023 and using established frameworks, we systematically integrate molecular, epigenetic, and algorithmic approaches. Unlike previous reviews, these works provide a broad, methodologically transparent review of biological aging measures and also lay the groundwork for developing a more systematic, focused review of socioeconomic, clinical, and demographic factors in biological aging. In doing so, we overcome the challenges that have deterred other scholars—such as the field’s rapid expansion, data fragmentation, interdisciplinary complexity, and methodological variability—and contribute a credible, impactful map of current knowledge, key limitations, and future directions for research.

Key questions surrounding the measurement of biological age with respect to chronological age or mortality emerge throughout the literature assessed for this scoping review. Some measurement questions are methodology specific, while other questions pertain to variables other than genetic and epigenetic.

To consider these questions, we undertook a scoping review study of those topics, focusing on works published during a 12 year window between 2011 and 2023. A scoping review, rather than a systematic review, was employed to expose relative strengths and shortcomings within the broader field of research. The purpose of a scoping review is to map available evidence for a given domain of knowledge, and to identify those persistent key questions and gaps in available evidence. Scoping reviews canvas available knowledge in a given field, and are an appropriate first step prior to a more focused systematic review. Scoping reviews are not intended as a critical analysis of the contributing studies, nor as a means for drawing higher order comparisons or knowledge synthesis. The identification of key questions to pursue within the domains of molecular biological age indicators, algorithmic approaches and their relationships and applicability are prioritized. The reader may choose to focus on particular sections of this review that are especially relevant to their own research.

## 2 Materials and methods

This review adheres to guidelines in the PRISMA Extension for Scoping Reviews (Tricco et al., 2018), and was guided by Arksey and O’Malley’s methodological framework (Arksey and O’Malley, 2005).

### 2.1 Search strategy

Four databases were searched from January 2011 to June 2023: PsycINFO, CINAHL, PubMed, and SPORTDiscus. Our search strategy included terms associated with biological and chronological aging in the abstract and title. Additionally, searches included relevant MeSH headings. Limits were applied to our search findings, specifically publication date (January 2011 to end of June 2023), subject (humans), age (adults aged 18 or older), and publication language (English). Refer to Appendix A for the full search strategy and search terms used.

### 2.2 Eligibility criteria

#### 2.2.1 Population

To be included, studies needed to include only adult human participants. Studies were included if they examined healthy adults or adults with specific health conditions (e.g., cancer). Studies including use of human-derived cell lines and human remains were retained.

#### 2.2.2 Outcomes

Studies chosen for inclusion compare a molecular indicator of biological aging with chronological age. Studies which examined the biological aging of only a specific organ were excluded. Only those biological age measures based on non-methylation data were included in this work.

#### 2.2.3 Design

Empirical primary research studies were considered for inclusion if they were peer-reviewed and published in a journal. Non-research, theses, dissertations, theoretical papers, seminar papers, case studies, book chapters, study protocols, review articles, and articles in-press during the period assessed were excluded. Emphasizing published, peer-reviewed studies prioritizes data quality and transparency and ensures that the synthesis reflects well-vetted research and established patterns. By placing methodological rigor, reliability, and credibility at the forefront, this review provides a solid foundation for future inquiries and expansion of the field’s knowledge base. We did not impose any limits regarding study size or number of participants in a given study; even small or modestly sized studies may reveal important insights for this field.

#### 2.2.4 Study selection and data charting

Reviewers used the software platform Covidence (Covidence, 2024) to screen article titles and abstracts. Two reviewers examined each title and abstract, with conflicts resolved by a third reviewer. Full text articles were next screened, with conflicts resolved by three reviewers re-examining and discussing the article in the context of inclusion criteria.

Data was extracted by reviewers, including the aims, population, methodology, and findings of the studies. Extracted data were then verified by one additional reviewer.

#### 2.2.5 Data synthesis

A composite list of all outcomes which were compared to chronological and/or biological indicators of aging was composed and categorized, resulting in 6 groups which are described in Table 1. Due to the breadth of information summarized by our work, we have focused on the underlying molecular biological features of biological aging, as well as the algorithms and clocks established for determining biological age.

**TABLE 1:** Categories of biological aging data analyzed in this review.

Categories
Mitochondrial DNA
Gene expression
Telomere length
Dunedin pace of aging
Klemera-Doubal algorithm
Novel and specific clocks
Other non-methylation features

## 3 Results

### 3.1 Study characteristics

**FIGURE 1:**
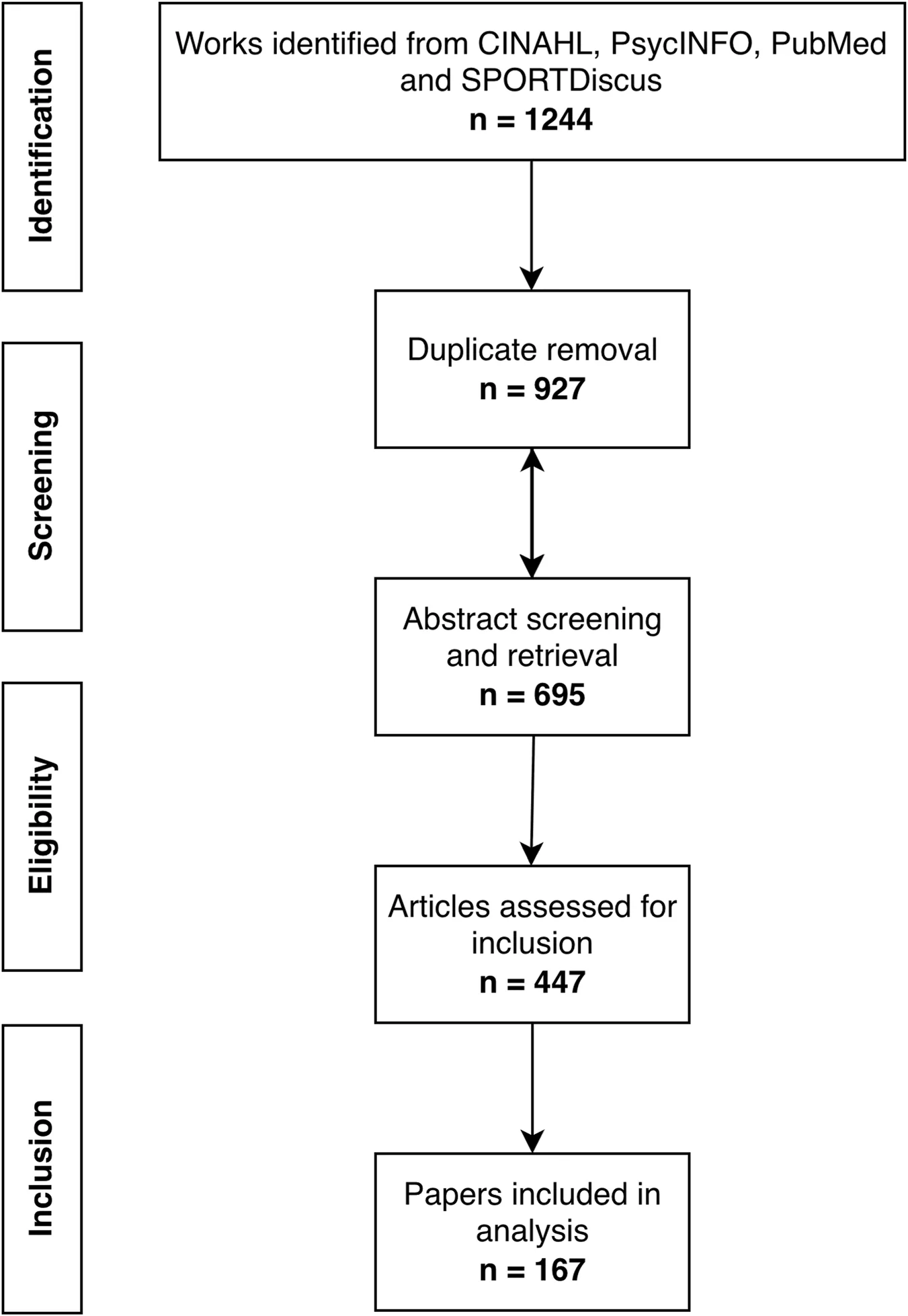
Flowchart detailing paper selection and filtration.

We identified 1,244 works from four databases during our data collection phase, of which 317 were duplicates (Figure 1). The remaining 927 records were screened for title and abstract, and 232 were removed. From 695 full text articles, of which one could not be retrieved, we excluded another 248 based on our inclusion and exclusion criteria. This resulted in 447 articles for analysis. Prior to preparing this review, we excluded additional works for using exclusively non-molecular metrics of biological aging, resulting in 435 papers; of these, 309 included non-methylation-based biomarkers of aging, which were analyzed in this review.

The total number of participants in the included manuscripts is 3,185,571; due to overlap in datasets between some studies, the number of unique participants is lower. A total of 146 studies used data from a non-unique dataset: the most frequently used datasets included the National Health and Nutrition Examination Survey (NHANES) in 21 manuscripts, the Health and Retirement Study (HRS) in 9, the Dunedin Cohort in ten, the Normative Aging Study (NAS) in five, and the Lothian Birth Cohorts of 1921 and/or 1936 in three. Twenty three studies used data from multiple datasets. The remaining studies considered populations unique to those publications.

Studies focused on different population parameters: some studies selected participants based on age, with 114 studies considering middle-aged and older adults. Seven studies focused on young adults, 114 studies included young and middle-aged adults, 35 studies focused solely on middle-aged adults, and nine studies analyzed long-lived individuals including centenarians. Some studies focused on health outcomes, with 15 studies considering mental and cognitive health, and 41 studies focusing on specific physical health conditions including but not limited to chronic cardiovascular disease, ischemic stroke, or infertility. Five studies included cancer patients and survivors, and six studies examined HIV positive individuals. Twenty two studies examined lifestyle parameters, including eight which focused on athletes, and one on tobacco smokers. Career influence on biological age was examined in eight studies, while four studies examined differential aging rates in twins. Some studies specifically recruited by sex, including 25 studies involving females only, and 21 studies with only males. A majority of the included studies examined the general population rather than focusing on subpopulations. Table 2 summarizes the study characteristics of those works reviewed here.

**TABLE 2:** Characteristics of those studies analyzed in this review. NHANES: National Health and Nutrition Examination Survey; HRS Health and Retirement Study; NAS Normative Aging Study; LBC21/36 Lothian Birth Cohort(s) of 1921 and/or 1936.

Characteristic	# of studies	Percentage
Location		
United States	94	30.4%
United Kingdom	17	5.5%
China	22	7.1%
Other countries	158	51.1%
Multiple countries (including above)	16	5.2%
Not reported	2	0.6%
Age		
All adults (18–99)	72	23.3%
Young adults (18–29)	106	34.3%
Adults (30–59)	247	80.0%
Older adults (60–99)	234	75.7%
Centenarians (100 +)	9	2.9%
Gender		
Mixed	263	85.1%
Female only	25	8.1%
Male only	21	6.8%
Study populations		
NHANES	21	6.8%
HRS	9	2.9%
Dunedin cohort	10	3.2%
NAS	5	1.6%
LBC21/36	3	1.0%
Other non-unique	146	47.2%
Multiple datasets	23	7.4%
Unique datasets	115	37.2%
Outcomes		
Mental health outcomes	15	4.9%
Physical health outcomes	41	13.3%
No outcome focus	253	81.9%
Lifestyle parameters		
Athletes	8	2.6%
Tobacco smokers	1	0.3%
Career choice	8	2.6%
Other lifestyle parameters	5	1.6%
No specific parameters	287	92.9%

**FIGURE 2:**
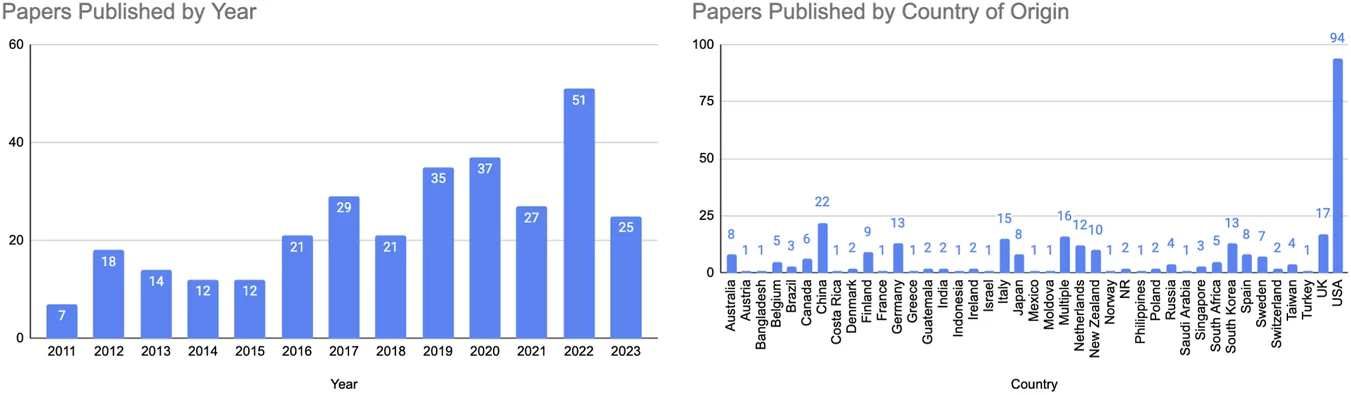
Left panel: papers published per year analyzed in this review, with 2023 data collection ending in June. Right panel: country of origin for studies included in this review, which provides information regarding the ethnic and racial composition of study populations included in this work.

Publication on biological aging increased steadily during the period of time reviewed (Figure 2A). Overall, studies were conducted in 39 countries, with the most frequent being the United States (94 manuscripts), followed by 22 in China, and 17 in the United Kingdom. Sixteen studies occurred in multiple countries, and two studies did not report the country they occurred in. Figure 2B details the breakdown by country for those works included in this manuscript.

**FIGURE 3:**
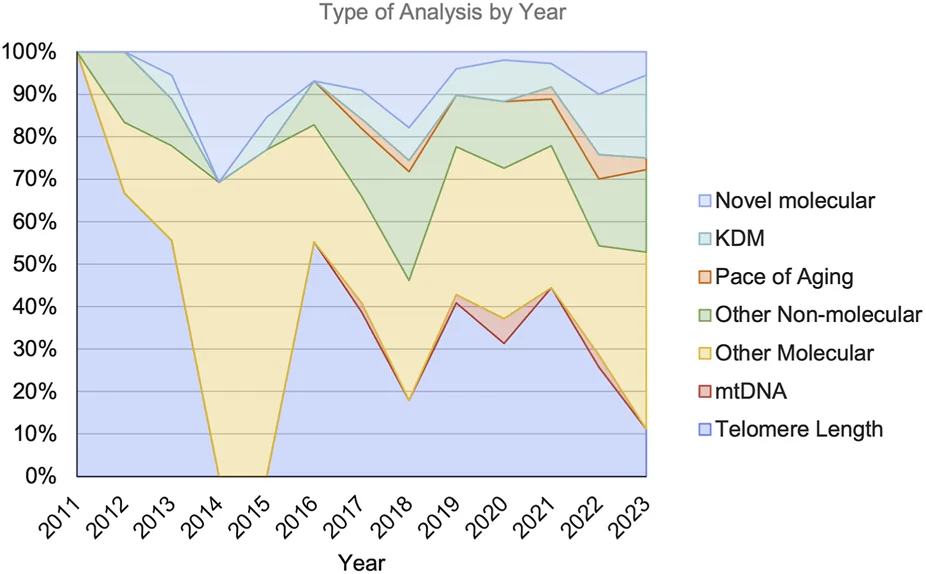
Types of biological aging analyses conducted per year as a percentage of works published in that year.

All manuscripts reviewed included one or more assessments of biological age, with telomere length employed by just over half of the studies. Several published studies describe clocks developed based on novel data sources, such as locomotor activity, facial photographs, or blood biomarkers. Other measures including mitochondrial DNA copy number, transcriptome levels and allostatic load are represented in the papers reviewed here. Figure 3 describes the breakdown of analyses employed in papers published per year.

### 3.2 Molecular biology features

#### 3.2.1 Mitochondrial DNA copy number

Mitochondrial DNA (mtDNA) copy number and mitochondrial function are both thought to decline with age. A study conducted with participants from the NAS observe an association between decreased mtDNA copy number and chronological age, as well as with biological age as determined by the PhenoAge epigenetic clock and with decreased telomere length in a follow-up visit (Dolcini et al., 2020). In a 2017 study on the effects of fine particulate air pollution exposure, Nwanaji-Enwerem and colleagues found that while mtDNA copy number did not significantly associate with chronological age, it was significantly negatively correlated with Horvath’s pan-tissue clock (Nwanaji-Enwerem et al., 2017). Similarly, a pair of studies derived from the VITamin D and OmegA-3 TriaL-Depression Endpoint Prevention (VITAL-DEP) found no association between mtDNA copy number and chronological age (Vyas et al., 2019; Vyas et al., 2020). However, a paper published by Praveen *et al.* describe a significant negative correlation between mtDNA copy number and chronological age, with mtDNA copy number higher in women than men among participants younger than 60 years of age (Praveen et al., 2020). Furthermore, they found significant positive associations between mtDNA copy number and telomere length, plasma folate levels, and vitamin B12 levels among older participants. A study conducted with the HRS population reports a modest association between increased difficulty in activities of daily life and mtDNA copy number (Crimmins, 2020). Studies conducted in 2022 report a positive association between mtDNA copy number and healthy lifestyle, a negative association with suicidal ideation and psychological distress, but no significant association with adverse childhood experiences (Hautekiet et al., 2022; Iannarelli et al., 2022).

### 3.3 Gene expression

Some studies reviewed have explored the relationship between aging and gene expression. A study conducted with African American participants of the Family and Community Health Study used the Peters transcriptome index to determine age acceleration and found associations with histories of both juvenile and adult social adversity and weakly, with male sex, and increased aging (Peters et al., 2015; Simons et al., 2019). Gheorghe and colleagues analyzed three tissue microarray datasets and found tissue-specific time points when widespread changes in gene expression occur (Gheorghe et al., 2014). Validating previous findings established in the InCHIANTI study population, Holly *et al.* describe a set of five differentially expressed genes, LRRN3, GRAP, CCR6, VAMP5 and CD27, which were similarly identified in the San Antonio Family Heart Study population, three of which (LRRN3, CCR6 and CD27) were additionally validated in the Exeter 10,000 study population (Holly et al., 2013). In a pair of studies first analyzing the Framingham Offspring cohort and following up with the Framingham Generation 3 cohort and the Rotterdam Study, 448 and 481 genes respectively were identified differentially expressed according to biological and chronological age (Lin et al., 2017; 2019). A 2016 study describes upregulation of the genes telomerase and TPP1 associated with longer telomeres in endurance athletes (Denham et al., 2016). Three related studies investigating ocular health found that in HIV negative individuals, expression of CDKN2A was correlated with chronological age, but not in HIV positive individuals (Pathai et al., 2013b; Pathai et al., 2013c; Pathai et al., 2013a). Rentscher and colleagues also examined expression of CDKN2A and found its expression was not correlated with chronological age, but rather with chronic stress exposure, perceived stress, and accumulated daily stress (Rentscher et al., 2019; Rentscher et al., 2020). In contrast, a study of CDKN2A and Nrf2 gene expression found that CDKN2A expression was significantly correlated with chronological age, while Nrf2 was not, in a population of late-stage renal disease patients (Sumida et al., 2020). Histidine-rich glycoprotein expression was positively associated with increased mortality risk and may represent an easily measured indicator of biological aging (Hong et al., 2020). A small study of pathogenic *de novo* DNA methyltransferase 3A (DNMT3A) mutations found affected patients exhibited accelerated aging, with different rates of aging observed with different mutations of DNMT3A (Jeffries et al., 2019).

#### 3.3.1 MicroRNAs

MicroRNAs (miRNAs) can regulate translation of genes by binding the untranslated regions of their target mRNAs. In a 2014 study of 20 breast cancer patients, Hatse and colleagues identified 15 miRNAs that were differentially expressed with age (Hatse et al., 2014). In a small cell culture study, it was found that rotenone-induced cellular stress could upregulate expression of mir-663, and that cells derived from older donors showed higher levels of induction than younger donors (Waaijer et al., 2014). A study of 78 participants including super-centenarians revealed that the miRNAs mir-21-5p, associated with the inflammatory response, and mir-126-3p, associated with angiogenesis, were both shown to increase with chronological age except in the ultracentenarians studied (Accardi et al., 2022).

#### 3.3.2 Polygenic disease risk

In a 2020 paper, Crimmins and colleagues associate the relationship between polygenic disease risk scores (PGS) and various outcomes, using those risk scores as a proxy for biological aging of specific physiological systems. They find that among non-Hispanic white HRS participants there is an inverse association between the longevity PGS and overall mortality, as well as between the general cognition PGS and cognitive dysfunction outcomes (Crimmins, 2020). In a study combining data from the Women’s Health Initiative (WHI), Levine and colleagues identified a single nucleotide polymorphism (SNP) associated with earlier age at menopause and increased age acceleration (Levine et al., 2016).

### 3.4 Telomere length and attrition

Telomere length is a commonly measured feature of biological aging, and in the course of this review we collected 162 papers published that analyzed telomere length. The majority of the papers (76% or 123 publications) report a significant negative relationship between telomere length and chronological age. Of the remainder, (15% or 25 publications) found no statistically significant relationship between telomere length and chronological age; a minority (9% or 14 publications) did not explicitly characterize the relationship. Telomere length was used to assess the biological aging impact of many diverse variables.

#### 3.4.1 Demographic parameters and telomere length

Demographic variables including sex and ethnicity influence telomere length. Most publications report longer telomeres in female rather than male subjects (Ahola et al., 2012; Baranyi et al., 2022; Carroll et al., 2013; Carroll et al., 2019a; Cole et al., 2018; Geronimus et al., 2015; Honig et al., 2015; Katz et al., 2022; Lincoln et al., 2019; Meier et al., 2019; Verhoeven et al., 2014; von Falkenhausen et al., 2022) but a minority of publications reported the inverse (Banszerus et al., 2019; Meyer et al., 2016; McLachlan et al., 2020; Vetter et al., 2019). A study of 4,053 older Chinese adults found that telomeres can lengthen with time, and this effect was strongest in those who exhibited the shortest initial telomeres (Huang et al., 2021). Race and ethnicity also were associated with differing telomere lengths. In many studies, African Americans were found to have longer telomeres than non-Hispanic white participants (An and Yan, 2017; Kemp and Ferraro, 2021; Lincoln et al., 2019; Meier et al., 2019; Needham et al., 2019; Pearce et al., 2021). However, von Kanel and colleagues describe shorter telomeres in black South Africans as compared to socioeconomically-matched white South Africans (von Känel et al., 2015). Few studies reported on the effects of Hispanic ancestry, where individuals with Hispanic ancestry exhibited shorter telomeres than did non-Hispanic whites in an American study (Carroll et al., 2019a), but the longevity-exhibiting Nicoyans of Costa Rica exhibited modestly longer telomeres than other Costa Ricans (Rehkopf et al., 2013). Family history was also associated with telomere length: men with long-lived mothers exhibit longer telomeres, and studies suggest a heritability for telomere length of roughly 55% (Honig et al., 2015; Kemp BR and Ferraro, 2021; Khan et al., 2017).

#### 3.4.2 Physical parameters and telomere length

Numerous studies have investigated the relationship between physical features and telomere length. One frequently studied feature is body weight or body mass index (BMI), and report the association between shorter telomeres and an elevated BMI (Ahola et al., 2012; An and Yan, 2017; Carroll et al., 2019b; Kingma et al., 2012; Martens et al., 2018; Mehta et al., 2021; Meier et al., 2019; Shin et al., 2016b; Strandberg et al., 2011; von Känel et al., 2014; von Känel et al., 2015). At birth, telomere length was found to be positively associated with birth weight in males, however (Kuzawa et al., 2022). Elevated waist circumference was also identified in association with shortened telomeres, as was increased body fat (Aguiar et al., 2021; Lee et al., 2011; Révész et al., 2017; Shin et al., 2016a). Inversely, higher lean body mass or appendicular lean mass was associated with longer telomeres (Chen et al., 2021; Meyer et al., 2016). Weight loss reveals an association with elongation of telomeres in female subjects only in a study of bioenteric intragastric balloon recipients, but not in a study considering exercise, metformin, or combination treatment in overweight women (Carulli et al., 2016; Nwanaji-Enwerem et al., 2021). Elevated athleticism is associated with improved telomere retention with aging (Aguiar et al., 2021; Borghini et al., 2015; Denham et al., 2013; Denham et al., 2016; Hernando et al., 2020; Muniesa et al., 2017; Østhus et al., 2012; Seki et al., 2023). Conversely, Normando *et al.* report in a Brazilian study that decreased physical activity was associated with longer telomeres in female participants, with the inverse reported for men (Normando P et al., 2020). In many studies, however, regular physical activity was positively associated with telomere length (Carroll et al., 2013, 2019a; Hoen et al., 2013; Katz R et al., 2022; Kingma et al., 2012). However, attributes indicative of physical frailty yield inconsistent results (Arts et al., 2018; Pathai et al., 2013a; Bountziouka et al., 2022; Brouwers et al., 2015; Vetter et al., 2022). Increased fatigability was also associated with decreased telomere length (Katz et al., 2022).

Dietary considerations also influence telomere length. A study of the Korean Genome Epidemiology Study population found that a traditional Korean diet including seafood, whole grains, legumes, vegetables and seaweed was associated with longer telomeres than a Western diet emphasizing red and processed meat and refined grains (Lee et al., 2015). Among hypertensive patients, very low and moderate intake of dietary copper was associated with decreased telomere length, thought to be due to its antioxidant properties (Gong et al., 2022). Elevated serum levels of inorganic phosphate, linked to poorer diet, were similarly associated with shorter telomeres (McClelland et al., 2016; Shiels et al., 2011; Xie et al., 2023). Higher levels of vitamin C and potassium were associated with longer telomeres in a study of older Koreans (Lee D et al., 2017). Analyses of NHANES study data find that consumption of lower fat milk, higher dietary fiber, greater quantities of fruits and vegetables and lower blood levels of gamma-tocopherol are all associated with longer telomeres (Tucker, 2017; Tucker, 2018; Tucker, 2019; Tucker et al., 2021). Alcohol consumption and alcohol abuse are associated with shorter telomeres (Lee J et al., 2017; Shin et al., 2016b; Strandberg et al., 2012; von Känel et al., 2014; von Känel et al., 2015), as is cigarette use (Carroll et al., 2013; Carroll et al., 2019a; Erdmann et al., 2017; Kemp and Ferraro, 2021; Shiels et al., 2011; Strandberg et al., 2011; Topiwala et al., 2022; Watkins et al., 2016) and illicit drug abuse (Cabrera-Mendoza et al., 2023; Lin et al., 2021).

Certain SNPs have been associated with telomere length: the alternative allele for rs4880, in the SOD2 gene, was associated with shorter telomeres, as was the alternativee alleles for rs9939609 or rs2736100 (Hernando et al., 2020; Roberts et al., 2014; Shin et al., 2016a). Carriers of a mutant allele of the SERPINE1 gene exhibited longer than wildtype telomeres (Khan et al., 2017).

Blood-based biomarkers are also associated with telomere length. Elevated HDL cholesterol is associated with longer telomeres (Maeda et al., 2011; Révész et al., 2017) although at least one study reports a inverse relationship (Tian et al., 2017). Elevated triglycerides and glucose both associate with decreased telomere length (Tian et al., 2017), as do elevated C-reactive protein, IL-6 and TNF α , particularly when the latter two are both elevated (O’Donovan et al., 2011b; Révész et al., 2014). In studies of Australian men, Yeap and colleagues observed that circulating estradiol, IGF1, IGFBP3 and dihydrotestosterone levels are all associated with increased telomere length, while sex hormone binding globulin (SHBG) levels are negatively correlated (Yeap et al., 2016; Yeap et al., 2019; Yeap et al., 2020). A later study expanded upon this finding, noting that telomere length and low testosterone association was mediated by SHBG (Marriott et al., 2023). In older adults, high plasma ferritin levels are associated with shorter telomeres, while elevated folate and vitamin B12 levels correlate with longer telomeres (Liu et al., 2019; Praveen et al., 2020). Maeda and colleagues observe sex-specific associations with blood composition features and telomere length: red blood cell counts were positively correlated with lengthened telomeres in Japanese study participants of both sexes, while other blood parameters differed (Maeda et al., 2011; 2019). In a study of 55 women, elevated urinary malondialdehyde levels, indicative of oxidative stress, was positively associated with telomere length while urinary cortisol was negatively associated (Barha et al., 2017; Erdmann et al., 2017). Gut flora composition was found to be associated with methylated telomere length in a study of 43 women (Maeda et al., 2021a).

#### 3.4.3 Psychological parameters and telomere length

Mental health and psychological features are known to relate to telomere length. In a study of 468 American military veterans, greater hostility and difficulties controlling anger were significantly associated with decreased telomere length (Watkins et al., 2016). Research analyzing the experiences of 600 adults with mild to moderate cognitive impairment found that those who experienced physical, psychological, or especially combination abuse exhibited significantly shorter telomeres (Fang et al., 2021). In a study of healthy middle-aged women, shorter telomeres were associated with an increased propensity for mind wandering, but not perceived stress or rumination (Epel et al., 2013). Work-related stress has been investigated, finding longer telomeres among those who report less work-related exhaustio and greater learning opportunity at work, and shorter among those who report the effects of depersonalization (“burnout”), decreased social support, and increased reliance on workplace compensation behaviors (Ahola et al., 2012; Chmelar et al., 2017; Weber et al., 2019). However, other studies did not confirm the relationship between chronic stress exposure, perceived stress, and accumulated daily stress with telomere length (Rentscher et al., 2019). Results regarding the association between telomere length and major depressive disorder are mixed, with some studies finding a negative association (Mehta et al., 2021; Verhoeven et al., 2014; Wium-Andersen et al., 2017), a negative association only in conjunction with other history such as childhood physical neglect or racial discrimination (Chae et al., 2016; Lee J et al., 2017; Lincoln et al., 2019; Vincent et al., 2017), or no significant association (Hautekiet et al., 2022). No association between telomere length and autism diagnosis or hypersexual disorder diagnosis was detected in a pair of studies published in 2022 (Boström et al., 2022; Okazaki et al., 2022). In the Strong Heart Family Study, Indigenous American men with severe depressive symptoms exhibited shorter telomeres, but not men with less severe symptoms or women with any severity of depressive symptoms, while the Dunedin Study found men, but not women, with internalizing disorder and depression exhibited shorter telomeres (Shalev et al., 2014; Zhao Q et al., 2016). Other studies have explicitly identified no relationship between the presence of depression and an impact on telomere length (Ryan and McLoughlin, 2020). The presence of anxiety was associated with impaired telomere length (Hoen et al., 2013; Révész et al., 2014; Shalev et al., 2014).

Childhood adverse events, including physical, psychological or sexual abuse, may induce a change in telomere length, but some studies demonstrated the absence of an association (Iannarelli et al., 2022). In a study of Finns separated from their parents during the Second World War, it was found that those children separated from both parents who also experienced physical or psychological abuse exhibited shorter telomeres than participants without those experiences, decades later (Savolainen et al., 2014). Mason *et al.* found no association between telomere length and a history of childhood abuse in a study of 1,135 women recruited for the Nurses’ Health Study II (Mason et al., 2015). Contrary to expectations, former Swiss indentured child servants who exhibited PTSD had longer telomeres than control individuals (Küffer et al., 2016). In other studies, PTSD was associated with shorter telomeres, and increasingly shorter telomeres for those individuals who also experienced childhood abuse (O’Donovan et al., 2011a).

Mental health interventions may offer some remedy: a study on the effects of meditation practices found that lovingkindness meditation was associated with reduced telomere loss (Le Nguyen et al., 2019). In a small study of myocardial infarction (MI) patients and control individuals, 60 days of relaxation practice preserved telomere length in controls only (Pavanello et al., 2019). Additionally, sleep and sleep quality have been assessed in conjunction with telomere length. Work done with 672 participants of the Multiethnic Study of Atherosclerosis (MESA) found associations between reduced telomere length and severe obstructive sleep apnea and other sleep parameters (Carroll et al., 2019a). A study in the Korean Genome Epidemiology study found that time spent snoring during sleep was associated with decreased telomere length (Shin et al., 2016b). Certain prescription sleep aids were associated with telomere attrition in men (Maeda et al., 2021b). Delayed circadian rhythm, self-reported moderately late chronotype, chronic short sleep and later sleep onset time were all associated with shorter telomeres, while self-reported extremely early chronotype was associated with longer telomeres, in a study of 2,936 Dutch adults (Wynchank et al., 2019).

#### 3.4.4 Clinical parameters and telomere length

Health and disease states have shown associations with telomere length. In reproductive health, telomere length was significantly positively associated with the number of surviving offspring a woman has in a small initial study, and in a further study, experience of child mortality was significantly negatively associated (Barha et al., 2016, 2017). Smeets and colleagues found that there is a positive correlation between gestational age and telomere length in young adults (Smeets et al., 2015). Telomere length was associated with maternal energy reserves as represented by arm fat area (Kuzawa et al., 2022). No link with pre-eclampsia was detected for telomere length in a 2022 study, nor was a link detected with early versus normal ovarian aging in a separate study of 97 women (Christensen et al., 2021; Pruszkowska-Przybylska P et al., 2022).

Overall risk of mortality typically correlates with decreased telomere length, although not reported by all studies (Gao et al., 2019; Glei et al., 2015; Honig et al., 2012; Li et al., 2020). Diabetes and diabetes duration are associated with telomere loss (Al-Daghri et al., 2021; Carroll et al., 2013; Cheng et al., 2020; Lee D et al., 2017; Tian et al., 2017; Tucker, 2022); diabetes interventions were found to lengthen telomeres in participants, particularly among those with the shortest telomeres (Hovatta et al., 2012). Similarly cardiovascular disease and cardiovascular parameters, including retinal microvasculature, are associated with telomere length (Cheng et al., 2020; Denham et al., 2013, 2016; Hammadah M et al., 2017; Hastings et al., 2019; Honkonen et al., 2020; Katz et al., 2022; Maeda et al., 2018; Mehta et al., 2021; Østhus et al., 2012; Raschenberger et al., 2013; Révész et al., 2014; Shokr et al., 2022; Zhang et al., 2014c). In a small study of patients implanted with cardioverter defibrillators (ICDs), those who had received therapy from their ICDs had reduced telomere length than did those who had not received ICD therapeutic intervention (Sawhney et al., 2016). A study of Italian early onset acute MI patients revealed an association with hypertensive status and reduced telomere length but did not associate early onset MI with reduced telomere length (Russo et al., 2012). Among chronic obstructive pulmonary disease (COPD) patients, telomere length was not associated with spirometric lung function measurements, but was associated with the 6 minute walk distance and mean daily step count measures (Wan et al., 2021). A small study of spinal surgery patients found that patients with the shortest telomeres were most at risk for post-operative complications (Safaee et al., 2023).

Diagnosis or history of cancer was associated with shortened telomeres, and colorectal tumor tissues exhibit shorter telomeres than adjacent cancer-free tissue (An and Yan, 2017; Gao et al., 2019; Katz et al., 2022; Maxwell F et al., 2011). A study of early breast cancer patients found that telomeres were not shorted by 12 months’ chemotherapeutic treatment (Brouwers et al., 2016). Infection with HIV was associated with a reduction in telomere length (Mehta et al., 2021; Minami et al., 2019; Pathai et al., 2013b; Pathai et al., 2013c; von Känel et al., 2014, von Känel et al., 2015). In a study of fibromyalgia, it was found that telomeres were shortest among those patients with higher pain and more severe depressive symptoms (Hassett et al., 2012). Multiple sclerosis patients were found to have shorter telomeres than age-matched controls but did not exhibit an accelerated loss relative to controls (Habib et al., 2020). A study of schizophrenia patients found that while there was no significant impact to telomere length based on diagnosis alone, there was an interaction between sex and diagnosis in which schizophrenic women showed the greatest reduction in telomere length relative to healthy controls and schizophrenic men (Wolkowitz et al., 2017).

Dhillon and colleagues reveal reduced telomere length among carriers of the APOE- ε 4 allele, and an inverse relationship between plasma levels of advanced glycosylation end products and telomere length (Dhillon et al., 2020). A 2020 study found that Alzheimer’s disease patients exhibited accelerated telomere loss relative to individuals with mild cognitive impairment (MCI), contradicting a previous 2012 study that found that MCI patients exhibited shorter telomeres than controls and Alzheimer’s disease patients, and that those MCI patients who progressed to Alzheimer’s disease exhibited longer telomeres than those who did not progress (Lee et al., 2020; Movérare-Skrtic et al., 2012). Work with the Washington Heights-Inwood Community Aging Project population found that in women, telomeres were shorter in those with dementia and were also a risk factor for developing dementia (Honig et al., 2012). Related, a 2022 study describes an association between elevated aging in the brain and shorter telomere length (Yu et al., 2022). A study of elderly adults without dementia found shorter telomeres associated with greater degree of subcortical atrophy as detected by magnetic resonance imaging (MRI) (Wikgren et al., 2014). While not associated with rheumatoid arthritis (RA) severity, a study including 145 RA patients and 87 controls found that reduced telomere length was associated with a worse coronary artery calcium score in RA patients (Ormseth et al., 2016). Patients with Prader-Willi syndrome (PWS) exhibit lower median telomere length than both controls and non-PWS participants born short for gestational age and subsequently treated with growth hormone (Donze et al., 2020).

#### 3.4.5 Environmental parameters and telomere length

Features including living environment and pollutant exposure may influence telomere length. Satisfaction with one’s neighborhood correlates positively with telomere length (Geronimus et al., 2015). A study examining the KORA F4 and NAS cohorts found that air pollution components had sex-related effects, where only men showed telomere shortening in response to increasing black carbon particulate exposure (Ward-Caviness et al., 2016). An additional study on air pollution’s effects on telomere length found that for males, mid-adulthood exposure was especially impactful, while for females, air pollution exposure levels perinatally were more critical (Baranyi et al., 2022). Exposure to organophosphate insecticides caused shortened telomeres among those in the second quartile of exposure, according to a study of 1724 NHANES participants (Ock et al., 2020). Data derived from the Scottish Family Health Study found that exposure to second hand cigarette smoke caused telomere attrition (Lu et al., 2017). An inverse association between telomere length and total dietary exposure to persistent organic pollutants and polychlorinated biphenyls (PCBs) was identified in a 2022 study (Alonso-Pedrero et al., 2022). Among women, higher urinary phthalate and phenol metabolites were associated with both an increased risk of breast cancer and telomere attrition, with telomere length being a modifier in the association between exposure and breast cancer risk (Zhang et al., 2022).

#### 3.4.6 Social parameters and telomere length

Socioeconomic status is frequently studied in conjunction with telomere length. Higher household income is positively associated with telomere length (Chae et al., 2016; Shiels et al., 2011; Yen and Lung, 2013) as is higher educational attainment (Honig et al., 2015; Geronimus et al., 2015; Kingma et al., 2012; Surtees et al., 2012), marriage (Yen and Lung, 2013) and home ownership (Carroll et al., 2013). A study of Detroit residents found that the effects of poverty led to greater telomere attrition in white participants than in African American participants (Geronimus et al., 2015). However, social class was not associated with telomere length (Surtees et al., 2012) or unexpectedly, was found to be associated negatively with telomere length in men (Yu et al., 2015). The West of Scotland Twenty-07 Study found that members of different generations exhibited different response patterns to socioeconomic conditions (Robertson et al., 2012). Parental effects are also observed, where a parent’s occupation or educational attainment influences offspring telomere length (Carroll et al., 2013; Kajantie et al., 2012; Normando et al., 2020). Reports of lifetime discrimination were significantly associated with decreased telomere length (Carroll et al., 2022). Other influences included a high belief in justice for the self being associated with reduced loss of telomeres (Lucas et al., 2018) and perceived unfair treatment associating with increased loss of telomeres (Rej et al., 2020). Telomere length was associated with land use mixture increase, particularly among foreign-born and less acculturated Mexican Americans (Zhao H et al., 2021).

### 3.5 Other molecular biomarkers

#### 3.5.1 Non-genetic biomarkers

Non-genetic markers, including serum protein or metabolite levels, and physiological features such as forced expiratory volume or systolic blood pressure, are often used to assess biological age. Studying middle-aged adults, Levine found that a set of merely seven blood-based biomarkers: C-reactive protein (CRP), glycated hemoglobin (HbA1C), serum alkaline phosphatase, forced expiratory volume (FEV1), systolic blood pressure and in men, cytomegalovirus optical density and serum albumin while in women, total cholesterol and serum urea nitrogen, were highly informative with regards to biological age, and produced significantly better mortality prediction than chronological age (Levine, 2013). Beam and colleagues use these markers to demonstrate in a study of 40 middle-aged twins, biological age significantly negatively correlates with physical functioning and certain aspects of memory (Beam et al., 2020). In the same year Crimmins describes work on the HRS population, finding these markers associated with increased difficulty in activities of daily life, multimorbidity, cognitive dysfunction and mortality (Crimmins, 2020). These markers were also used in a study that found that African Americans exhibited an elevated biological age, and that the risk of mortality was greater in African Americans relative to white study participants (Levine and Crimmins, 2014). Using a subset of these markers, it was found that the rate of biological aging is changing over time: while biological age is lower for younger subpopulations, improvements in biological age deceleration are greatest among the oldest male study participants, possibly due to cultural shifts including smoking cessation (Levine and Crimmins , 2018). Studying a further restricted subset of these markers in a large merged data set found that 481 genes significantly change their expression levels responsive to this biological age measure (Lin et al., 2019). Using a set of 22 biomarkers, a 2023 study identified accelerated biological aging in non-Hispanic Blacks and Hispanics relative to non-Hispanic whites regardless of sex, which was modified by educational attainment (Farina et al., 2023).

An overlapping group of clinical biomarkers was identified in research derived from the Canadian Study on Health and Aging population, where serum albumin, calcium, serum creatinine, diastolic blood pressure, hemoglobin, serum alkaline phosphatase, inorganic phosphorus, total protein, thyroid-stimulating hormone (TSH) and blood urea nitrogen were found to be significantly correlated with chronological age (Mitnitski et al., 2017). The same set of biomarkers were used in a study combining the Rotterdam Study and NHANES study populations and found that this measure of biological age was a better predictor of diabetes, stroke, cancer and mortality than chronological age, and roughly equivalent at assessing the risk of coronary heart disease and all-cause mortality (Waziry et al., 2019). In an investigation of the BASE and BASE II study populations, a novel set of biomarkers was found to be more accurate than the Vidal-Bralo or PhenoAge clocks in determining mortality and subjective health (Drewelies et al., 2022). A 2021 paper describes a combination of Levine’s markers along with the Targeted Aging with Metformin (TAME) assay and found that these together were good predictors of multimorbidity, but not better than chronological age at predicting cognitive dysfunction (Barzilai, 2017; Crimmins et al., 2021). A 2021 study in the HRS population found biological age as determined by Levine’s biomarkers was associated with persistent feelings of loneliness and social isolation (Crowe et al., 2021). These biomarkers were also associated with elevated physical activity among COPD patients and improved biological age, as well as with a diet relatively high in carbohydrates with moderate lipid and protein components, associating the marker set with general health (Senior et al., 2022; Wan et al., 2021).

In the Singapore Longitudinal Aging Study population, a study found that 8 and 10 biomarkers adequately described biological aging in men and women respectively, and that a wide range of behavioral and socioeconomic factors were linked to biological aging, including housing type, marital status, physical activity and diet (Ng et al., 2020). Using only physiological and physical fitness variables, Jee and colleagues describe a set of 8 partially overlapping parameters for each sex that model biological age in the Korean population, and they describe an association of advanced biological age with both elevated BMI and sarcopenia (Jee et al., 2012). Later work with the Korean KNHANES III study examined a set of seven biomarkers: systolic blood pressure, waist circumference, glutamic oxaloacetic transaminase, ferritin, blood urea nitrogen, creatinine, and FEV1, finding an association of biological age with both diabetes and increased glucose tolerance (Jee and Park, 2017). Meisel and colleagues developed a blood-based biomarker panel with significantly better predictive power of tooth loss than chronological age (Meisel et al., 2019). Examining a panel of 36 blood biomarkers, a study of 4,592 participants found that those with a higher polyphenol antioxidant diet exhibited a lower difference between chronological and biological age, particularly among men (Esposito et al., 2021). Work examining 144 plasma protein profiles and anthropometric measurements found dietary and lifestyle effects associated with changes in biological age, finding consumption of fatty fish and moderate daily coffee beneficial, with smoking, high soda consumption, obesity and minimal exercise detrimental (Enroth et al., 2015).

In a study of 1,581 older adults, parameters including body composition, energy availability and consumption, homeostatic equilibrium measures, and neurodegenerative and neuroplastic measures were assessed in relationship with chronological age, finding a substantial number of features that contributed to biological age (Kuo et al., 2020). 12 metabolites linked to the fatty acid/tri-citric acid cycle metabolism and glycolysis predicted a biological age significantly correlated with chronological age in a set of 604 older adults (Johnson et al., 2019). In a study of 47 plasma metabolites, 37 were found to be significantly associated with age, including those involved in bioenergetic pathways, suggesting a decrease in mitochondrial beta-oxidation and accumulation of unsaturated fatty acids leads to an increased oxidative damage and inflammation with age (Sol et al., 2023). Thirty-four inflammation, vitamin B and kynurenine pathway blood biomarkers were assessed for their biological age predictive capacity; 29 were associated with chronological age, 17 were found to be associated with mortality at followup, and a score composed of 10 biomarkers was associated with higher all-cause mortality in the study population (Dugué et al., 2022). Among 3,558 participants in a wellness program, Earls and colleagues found that biological age as derived from proteomics data outperformed laboratory results and metabolite panels in correlation with chronological age and mean absolute error (Earls et al., 2019). They found health conditions including obesity, hypertension, elevated cholesterol, lung infection, type II diabetes and breast cancer associated with this measure of biological aging, and that participants in the wellness program exhibited a reduced biological age after program completion. In a study examining the Moli-Sani study population, increase in energy-adjusted dietary inflammation index was associated with increased biological age as estimated by blood biomarker levels (Martínez et al., 2023). Other efforts to leverage proteomics data for biological age determination include mass spectrometry-based analyses: a measure based on five peptide fragments including apolipoprotein A1 (ApoA1), fibrinogen alpha chain, complement C3, complement C4a, and breast cancer type 2 susceptibility protein was described in a 2020 publication, while a 2019 study employs mass spectrometry data biomarkers developed in the MARK-AGE study, finding that for HIV patients, biological age was elevated relative to healthy controls (De Francesco et al., 2019; Bürkle et al., 2015; Cao et al., 2020). A 2022 study demonstrated both positive and negative correlations with chronological age for urinary oxidative stress markers, suggesting these as a useful metric for calculating biological age (Mukli et al., 2022). Saliva has also been explored as a source of biological aging data: in a study of 79 saliva proteins, 69 were found to decrease in healthy elderly participants, while 37 proteins increased in Alzheimer’s participants (Contini et al., 2022). Non-invasive cardiovascular measurements have also been assessed for their informational content: the left atrium reservoir strain, left atrium conduit strain rate and the ratio of left atrium conduit strain rate to booster strain rate were found to be reduced in older adults as compared to younger adults (Koh et al., 2023).

Specific studies focusing on related proteins have revealed patterns of pathogenesis. Work restricted to measurements of senescence-associated secretory phenotype (SASP) markers found an association between increased frailty and certain SASP markers among a merged cohort of elderly healthy adults, aortic stenosis surgery patients and ovarian cancer patients, with levels of GDF15, OPN and TNFR1 significant among all three subgroups (Schafer et al., 2020). A previous study had identified GDF15 as positively associated with increased morbidity, cognitive impairment and decreased functional independence, as well as being a significant mortality predictor in a study of older adults (Sanchis et al., 2019). In a study of older adults with late onset depression, it was found that depressed adults exhibited higher GDF15 serum levels, and that later onset of depression associated with elevated GDF15 (Mastrobattista et al., 2023). Further, a 2022 study involving 76 participants later identified a moderate correlation between GDF15 and chronological age in postmenopausal women, suggesting that SASP markers may be indicative of biological age (Shin et al., 2022). An investigation of a panel of nine inflammatory biomarkers was used to develop an inflammatory biological age measure, finding seven of the nine significantly correlated with chronological age (Lin et al., 2017). A study focused on five biomarkers found an association between CRP and BMI as well as waist circumference; the same study identified a correlation between GDF15 and smoking pack-years in men, but not women (Srour et al., 2022).

Other studies approached the question of disease-specific genes and proteins underlying biological aging. In studies of breast cancer, Brouwers and colleagues found association of IL-6, IGF1, MCP1 and TNF α with chronological age, and frailty was particularly associated with levels of IL-6 (Brouwers et al., 2015; Brouwers et al., 2016). Findings of significance for IL-6 and TNF α were confirmed in a 2022 paper, additionally linking circulating levels of those proteins to increased multi-morbidity risk (St Sauver et al., 2022). Circulating IL-37 levels were found to be lower in older adults compared to middle aged adults, and an association with the IL-37:CRP ratio with maximal oxygen consumption ( $VO_{2}$ max) and BMI was found; the IL-37:IL-6 ratio was also positively correlated with $VO_{2}$ max and lean body mass (Brunt et al., 2023). Berben and colleagues found combinations of blood biomarkers associated with frailty and tumor immune infiltrate characteristics in a study of 65 older breast cancer patients (Berben et al., 2021). A 2016 study explored urine metabolome data in a study of bariatric surgery patients, generating a metabolic age score that was predictive of survival and weight loss at 13 years of follow-up (Hertel et al., 2016). Fraszczyk and colleagues, also studying 40 bariatric surgery recipients, found an improvement in levels of liver enzymes following surgery, suggesting these markers may also reflect a metabolic biologic age metric (Fraszczyk et al., 2020). In individuals with mild traumatic brain injury, it was determined that chronological age at injury is associated with increased brain aging following that injury as determined by MRI (Amgalan et al., 2022).

Studies of hormone levels have revealed patterns underlying biological aging in different disease states. In a study of COPD patients, an inverse relationship with chronological age and significant depression of dehydroepiandrosterone and growth hormone levels was observed, suggesting the biological age of a COPD patient is as much as 24 years older than age-matched controls (Karametos et al., 2019). An Indonesian study of fertility-challenged women found a negative correlation between chronological age and antral follicle count and anti-Mullerian hormone, and a positive correlation with follicle stimulating hormone (Wiweko et al., 2013). Data from the Baltimore Memory Study was employed to identify a relationship between increasing waking cortisol levels and chronological age, as well as lower average waking cortisol levels and slower decline in African Americans, while socioeconomic vulnerability was associated with faster diurnal decline (Samuel et al., 2018). An additional study involving 91 participants found morning and evening cortisol secretion was highest in men with severely accelerated biological age, suggesting disruption of circadian rhythm may be a feature of accelerated biological aging (Gorelik et al., 2022).

Plasma protein levels have also revealed association with biological aging. A study of N-terminal fragment B-type natriuretic peptide precursor (NT-proBNP) found a significant association with NT-proBNP levels and mortality risk (Muscari et al., 2021). Plasma soluble urokinase plasminogen activator receptor (suPAR) was found to correlate positively with age, and elevated suPAR was associated with lower functional capacity, poorer cognitive function, accelerated facial aging and increased physical limitations (Rasmussen et al., 2021). suPAR was also elevated in older adults and associated with increased risk of cardiovascular disease (Koh et al., 2023). A significant positive association between levels of HbA1C and chronological age was identified in healthy study participants, suggesting that diabetics with elevated HbA1C may exhibit accelerated biological aging (Suvarna et al., 2017). A study of 701 middle-aged Han Chinese found 19 immunoglobulin G (IgG) glycans that were correlated with chronological age, 10 of which were consistently correlated in both men and women (Yu et al., 2016). And in a small study of men, terminal galactosylation of ferritin was posited as a biomarker of healthy aging (Dunston et al., 2012).

Macromolecular cellular features, such as the presence of damage foci and damaged telomeres, were found to be positively associated with donor chronological age in a study of cultured fibroblasts (Waaijer et al., 2016). The length, coverage area and curvature of dermis elastic fiber decreased with chronological age, suggesting that dermal characteristics can serve as a measure of biological aging (Waaijer et al., 2012). Hair whitening was found to be significantly linked to coronary artery disease, allowing for the potential of premature hair whitening to be an indicator of advanced biological aging (Kocaman et al., 2012). In a small study of cognitive function in women diagnosed with breast cancer, the authors found a positive association between leukocyte DNA damage and chronological age, and reduced executive function scores with increasing DNA damage (Carroll et al., 2019b).

#### 3.5.2 Allostatic load

Allostatic load is a measure of accumulated chronic stress on physiological systems, including cardiovascular function, renal function, immune system status and neuroendocrine systems and represents ‘wear and tear’ biological aging (Guidi et al., 2020). A study of the Lothian Birth Cohort of 1936 assessing the relationship between brain predicted age differences (brain-PAD) and biological aging found a positive association between worsening brain-PAD scores and allostatic load (Cole et al., 2018). Assessing sex-specific relationships with allostatic load, McCrory and colleagues found in a study of 490 older Irish adults that metabolic allostatic load was positively associated with epigenetic age acceleration in men, while cardiovascular and metabolic allostatic load were positively associated with epigenetic aging acceleration in women (McCrory et al., 2020). A later study found elevated allostatic load associated positively with all-cause mortality risk, poorer physical functioning, and lower educational attainment and material resources (Hastings et al., 2022). In a study of reproductive history among female participants in the NHANES population, premenopausal women showed no association between allostatic load and number of births or time since last birth, while postmenopausal women revealed a positive significant association between the number of births and allostatic load (Shirazi et al., 2020).

#### 3.5.3 Basal oxidation

Oxidation levels may be associated with biological aging. A small study using plasma levels of the anti-aging protein S-klotho found that elevated levels of S-klotho were associated with increased basal fat oxidation and decreased basal carbohydrate oxidation, but not associated with chronological age (Amaro-Gahete et al., 2019). A 2023 study reports a negative association between klotho levels and dietary inflammatory index (Xie et al., 2023).

#### 3.5.4 Physiological dysregulation

Physiological dysregulation incorporates data from multiple organ systems to assess age-related dysfunction. In a study of 93 male pilots, Bauer and colleagues measure 18 markers of organ function and find a non-linear relationship with increasing physiological dysregulation until middle age, and then a decline in overall physiological dysregulation (Bauer et al., 2020). A 2022 study characterized patterns of nutrition associated with minimal biological aging measured by physiological dysregulation, including moderate macronutrient uptake and ingestion of vitamins E and C, especially in combination (Senior et al., 2022). Using 12 biomarkers, a physiological dysregulation-based calculation was able to predict mortality risk in a Chinese population (Liu, 2021). Among hospitalized COVID-19 patients, a Russian study observed that with increases in physiological dysregulation, the risk of both deterioration and mortality increased (Strazhesko et al., 2022).

#### 3.5.5 Telomerase activity

Associated with telomere length is activity of the multi-subunit enzyme telomerase, which restores telomere length lost during DNA synthesis in cell division. Chronological age is reported as having an inverse relationship with telomerase activity (Carroll et al., 2019b; Fedintsev et al., 2017). In a study of patients who had received implanted cardioverter defibrillators (ICDs) following diagnosis of coronary artery disease and previous MI, those patients who had received therapy from their ICDs exhibited elevated telomerase activity relative to those who had not (Sawhney et al., 2016). A study of 94 women diagnosed with breast cancer found that executive function, attention, and motor speed all positively correlated with telomerase activity (Carroll et al., 2019b). Telomerase expression was found to be higher in endurance athletes with the highest cardiorespiratory fitness and lowest resting heart rates compared to less fit athletes and controls (Denham et al., 2016).

#### 3.5.6 Molecular markers of bone

For forensic or archaeological purposes, biological age of human remains may need to be determined. As blood-based and DNA-based biomarkers may be absent or degraded, methods to correlate biological age with skeletal features have been developed. In a 2017 study of 81 sets of Mexican and Mesoamerican remains, auricular surface (AS) and pubic symphysis (PS) were used to predict biological age of the decedents. Among those remains for whom chronological age was known, AS- and PS-based biological age prediction exhibited a mean difference of 4.1 and 4.5 years respectively (Couoh, 2017).

### 3.6 Biological aging algorithms and clocks

#### 3.6.1 Pace of aging

The ‘Pace of Aging’ third generation clock was developed in 2015 and is based on 18 biomarkers covering a variety of different organ systems (Belsky et al., 2015). In their seminal paper, Belsky and colleagues compared their Pace of Aging clock against a 10 biomarker clock and found they correlated moderately positively. The Pace of Aging measure correlated negatively with physical and cognitive ability measures, and positively with increased physical limitation. Pace of Aging also associated with qualitative assessments of aging, including self-rated health and facial aging. In a follow-up study, it was found that Pace of Aging was significantly correlated with the age of one’s longest-lived grandparent, childhood social class and health, IQ, self control and adverse childhood experiences (Belsky et al., 2017). A third study found weak correlation between Pace of Aging and methylation-based epigenetic clocks and telomere length (Belsky et al., 2018a). They also confirmed their earlier findings, with Pace of Aging significantly associated with physical functioning, cognitive functioning and self-rated health and facial aging. A 2021 paper found that suPAR, a biomarker of inflammation and biological aging, was positively correlated with Pace of Aging (Rasmussen et al., 2021). Adolescent behaviors including smoking, antisocial behavior, and obesity have been associated with accelerated Pace of Aging later in life (Bourassa et al., 2022; Langevin et al., 2022). Socioeconomic disadvantage was also associated with accelerated biological aging, and this effect was partly modified by differences in health behavior (Schrempft et al., 2022). A quartet of unhealthy behaviors (obesity, low physical activity, smoking and alcohol consumption) were also correlated with elevated Pace of Aging (Kankaanpää et al., 2022). However, the presence of macular drusen, a retinal defect associated with oxidative stress, was not associated with a difference in Pace of Aging biological age (Wilson et al., 2023). Using the Pace of Aging metric and facial aging measurements, work done in the Dunedin Study population found that participants with high quality relationships with low or absent intimate partner violence had the lowest pace of aging compared to those in lower quality, higher violence relationships (Bourassa et al., 2020).

#### 3.6.2 Klemera-Doubal algorithm

The Klemera-Doubal method (KDM) is often applied to clinical biomarker measures to assess biological age (Klemera and Doubal, 2006). Two key features of the algorithm should be highlighted: Klemera and Doubal conceptualized the problem as minimizing distance between regression lines and biomarker points within the multidimensional space of all analyzed biomarkers, and they recognized that chronological age itself could be a marker of biological age, although this last point is not without controversy. KDM has been employed steadily since its initial publication with a variety of overlapping sets of biomarkers. Originally proposed as an improvement upon multiple linear regression (MLR)-based approaches, this premise has been tested and found valid (Levine ME, 2013; Jee and Park, 2017; Zhong et al., 2020). In a 2013 publication, Levine compares KDM, MLR and principal component analysis (PCA) and found that a set of 7 biomarkers selected by PCA and used in a KDM calculation of biological age performed best in mortality prediction (Levine, 2013). A later paper compared the three approaches with a more expansive set of 68 clinical biomarkers and found that KDM-calculated biological age was the best predictor of frailty, especially in men; for mortality, all three algorithms outperformed chronological age (Zhong et al., 2020). In a study of 3,642 women, Jee and colleagues similarly found that in a comparison of KDM, MLR and PCA, KDM performed as well as PCA (Jee and Park, 2017).

Biological age as calculated by KDM was found to be superior to chronological age in predicting risk of diabetes, stroke and cancer, while chronological age was better suited to predicting risk of dementia and Alzheimer’s disease (Waziry et al., 2019). Work with the ACCORD trial detected a 12 year older biological age as calculated by KDM in diabetics as compared to healthy controls; prediabetics were 2.69 years older than healthy non-diabetics (Bahour et al., 2022). Similarly, work with the CARDIA study population detected higher odds of cardiovascular disease and stroke associated with elevated biological age (Forrester et al., 2022a). Major coronary event, ischemic stroke, or intracerebral hemorrhage were all associated with elevated biological age in a Chinese cohort (Chen et al., 2023). KDM-based biological age has also been associated with multimorbidity and all-cause mortality as well as mortality due to cancer and diabetes (Bahour et al., 2022; Chen et al., 2023; Crimmins, 2020; Dmitrieva et al., 2023; Forrester et al., 2022b; Graf et al., 2022; Shapiro et al., 2023). However, a study of hospitalized COVID-19 patients found no association between KDM-calculated biological age and mortality due to COVID-19 infection (Strazhesko et al., 2022). KDM-measured biological age has been found to differ between men and women, with different formulas being calculated for each sex in several studies (Johnson et al., 2019; Zhong et al., 2020). There may also be racial components to KDM-measured biological age: in a 2014 study using 10 biomarkers, it was found that African American NHANES study participants showed a higher biological age than did their white counterparts, leading to an overall 46% increase in risk of mortality, 40% higher cardiovascular mortality, and 51% higher cancer mortality (Levine ME and Crimmins, 2014). A pair of studies published in 2022 support these findings, observing that Black study participants were biologically older than white participants, and in the CARDIA study, an increased biological age in Black males led to a significantly increased risk of cardiovascular disease compared to white males (Forrester et al., 2022a; Graf et al., 2022). A 2021 study using 12 biomarkers found that the KDM calculation on those features was predictive of mortality (Liu, 2021). A KDM model trained on American NHANES data was also found to be predictive of mortality in a Taiwanese study population, suggesting there may be commonalities in biomarkers between racial groups (Gaydosh et al., 2020). Further, KDM-based biological aging rates are not static: a 2018 paper describes a decline in KDM-measured biological age acceleration in later generations, largely attributable to modifiable health-related behaviors including smoking cessation (Levine and Crimmins, 2018).

Decline in physical functioning is associated with elevated KDM-calculated biological age (Beam et al., 2020; Belsky et al., 2018b; Hastings et al., 2019). A study of the HRS population detected an association between limitations in activities of daily life and elevated biological age, and was significantly exacerbated in Black study participants (Graf et al., 2022). A 2022 study reported that worse physical and emotional health were associated with elevated biological age, also finding grip strength inversely associated with KDM-calculated biological age (Hastings et al., 2022). Similarly, declining cognitive function is associated with higher biological age (Beam et al., 2020; Belsky et al., 2018a; Crimmins, 2020; Gaydosh et al., 2020; Hastings et al., 2019). Short-delay free recall, self-reported health and disability, subjective aging parameters such as facial aging, increased comorbidities, hospitalization, and difficulty with activities of daily life associate with KDM-based biological age (Belsky et al., 2018b; Hastings et al., 2019; Zhong et al., 2020). Mental health impacts including perceived stress, experienced discrimination and depression also are linked to elevated biological age (Forrester et al., 2022b; Hastings et al., 2022). In a pair of studies using partially overlapping sets of inflammation-related biomarkers, Lin and colleagues studied whole blood gene expression changes associated with changes in KDM-calculated biological age (Lin et al., 2017; 2019). They found 448 genes whose expression level changed in response to biological age as measured by 7 inflammation-associated biomarkers, and 481 genes changed in association with 6 biomarkers in a later study. Urinary markers of oxidative stress were used to develop a KDM-based biological clock, indicating that urinary metabolites are a useful data source for biological aging (Mukli et al., 2022). Biological age as calculated by KDM was lower in calorie-restricted diet adherents versus those with no restriction (Belsky et al., 2018a). A study of macronutrient consumption found that above average consumption of carbohydrates in conjunction with typical consumption of proteins and lipids exhibited the lowest level of biological aging among dietary compositions (Senior et al., 2022). Inflammatory dietary potential is positively associated with biological age acceleration in a study of NHANES participants (Xie et al., 2023). In a study of obese older adults, it was found that either diet alone or in conjunction with exercise reduced biological age acceleration more than did exercise alone (Ho et al., 2022). Hydration was found to associate with biological aging: higher sodium serum levels, a proxy for hydration habits, resulted in a worsening KDM-calculated biological age (Dmitrieva et al., 2023). In a study of pre- and postmenopausal women, parity was associated with different rates of biological aging as measured by KDM, but only in postmenopausal women (Shirazi et al., 2020). The lowest rate of biological aging in this study was observed for women who had 3 or 4 live births.

A pair of studies assessing the relationship of socioeconomic and lifestyle features with KDM-calculated age were published in 2020, both making use of the Singapore Longitudinal Aging Study population. Both studies observed the age decelerating effects of increased education, although other features responded differently (Ng et al., 2020; Zhong et al., 2020). Ng and colleagues reported that accelerated biological aging was especially associated with being single, widowed or divorced, with living alone, housing type, unintended weight loss, smoking, elevated alcohol consumption, high nutritional risk or exhibiting poor nutrition (Ng et al., 2020). They also observed that marital status was more significant for men and diet more significant for women. In the study undertaken by Zhong and colleagues, they affirmed that marital status was significant for men only, but also observed health activity, social activity, productivity activity, recreational activity and cognitive stimulation were significantly associated with biological age (Zhong et al., 2020). Sleeping features including snoring and insomnia are associated with elevated biological age, and conversely an early chronotype and normal sleep duration were linked to improved biological aging (Gao et al., 2022). Environmental parameters including neighborhood resource availability and air pollution associate with KDM-calculated biological age (Forrester et al., 2022b; Pan R et al., 2023). A 2023 study observed that lower socioeconomic status exacerbated mortality risk associated with elevated biological age (Shapiro et al., 2023).

#### 3.6.3 Novel and specific clocks

The Health Aging Index (HAI) was used along with KDM-calculated biological age and homeostatic dysregulation measures to identify improvement in HAI among older obese adults who adhered to a diet and exercise weight loss treatment (Ho et al., 2022). Intrinsic Capacity (IC) score, which is based on cognitive and physical performance, as well as geriatric depression diagnosis associated a lower baseline IC with higher inflammatory marker levels, particularly TNFR1 and GDF15 levels (Lu et al., 2023). The MARK-AGE project biomarkers, which include DNA methylation as well as serum metabolites, peptides, and N-glycans, was used to analyze a population of HIV-positive and control blood donors (De Francesco et al., 2019). That study found that the HIV-positive participants were significantly biologically older, particularly male participants. BrainAge, which associates with chronological age positively, was also found to associate positively with depressive episodes, their duration, and age of onset in a study of 42 depressed patients (MJ et al., 2023).

Several studies analyzed for this review use markers of metabolic syndrome to calculate biological age. In a pair of studies published in 2018, Han and colleagues studied metabolic syndrome status-related biological age based on waist circumference, blood pressure, fasting blood glucose, serum triglycerides, HDL cholesterol, total cholesterol, and chronological age (Han et al., 2018a; Han et al., 2018b). They found increased biological age by this metric associated with male sex, increased BMI, chronic disease diagnosis including hypertension and diabetes, reduced sleep, lower educational attainment, marital issues, unemployment, low income, lack of exercise, being an ex-smoker and low consumption of alcohol, while decreased biological age was associated with higher usage of nutrition data and higher socioeconomic status. A 2018 study based on 18 physical parameters and biomarkers developed a biological aging clock that indicated increased risk of mortality, hypertension, diabetes, stroke and cancer (Kang et al., 2018). In a study of 263,000 participants, metabolic syndrome features were analyzed using PCA, revealing different aging formulas for men and women (Kang et al., 2017). Waist circumference, mean blood pressure, fasting blood sugar, serum triglycerides and HDL levels were all strongly associated with metabolic syndrome-based biological age, although to different degrees in men and women. An additional study established a metabolic syndrome-adjacent ‘body shape clock’, calculating different biological age formulations for men and women (Bae et al., 2013). Waist to hip ratio was found to be most strongly associated with chronological age among the markers considered, with hip circumference and height also showing strong association. Using a stacking model and 19 biomarkers, Yang and colleagues developed a novel clock with a mean absolute error (MAE) of 4.4 years in the test data set, which linked accelerating biological age with increasing comorbidity (Yang et al., 2022). Similarly, a clock using the XGBoost algorithm and only five biomarker features found association with both all-cause and cause-specific mortality (Wang et al., 2022). Using a limited number of methylation sites and measurements of signal joint T cell receptor arrangement excision circles (sjTRECs), Paparazzo *et al.* were able to produce a blood-based novel clock with a MAE of 4.43 years (Paparazzo E et al., 2022). In a study of Polish men, a body shape-based biological age was calculated based on ponderal index, waist circumference, chest index, pelvis-acromial index and total body water, finding that biologically older participants showed poorer performance on physical tests (Gołab et al., 2016). A separate study that considered female residents of Tiraspol used physical parameters and grip strength to divide the study into ‘slow aging’ and ‘fast aging’ subgroups found that their especially long-lived study participants were more likely to exhibit a lower BMI, shorter height, lower grip strength, lower skeletal muscle mass and fat mass, and higher systolic blood pressure (Negasheva et al., 2014). In a study of young women, a clock based on physical parameters and body shape revealed delayed biological aging associated with elevated weekly physical activity (Kolokoltsev et al., 2022). A ‘physical fitness age’ was found to be a better predictor of sarcopenia than was chronological age in a Japanese study, and a deficit in physical fitness age was found to be associated with a history of diabetes, obesity, and depression (Toyoshima et al., 2022).

In 2022, a study was published comparing a novel measure of physiological age, based on frailty, comorbidities, and limitations in activities of daily life, across different nations, revealing a worse physiological age among Italians, Israelis and Americans, while Swiss, Dutch, Greek, Swedish and Danish citizens exhibited an improved physiological age (Rapp et al., 2022). This study also found an association between biological age and education, income, and marital status. Using a multidimensional measure of disease status, risk factors, socioeconomic and functional status along with blood-based biomarkers, a 2023 study developed a clock that was more accurate than chronological age at predicting mortality (Salignon et al., 2023). Studies of facial photograph-based perceived age was found to be linked with body adiposity in a paper positing perceived age as a proxy for biological age (Żelaźniewicz et al., 2022). Other image-based studies have focused on skin; both found a positive association for their skin-based biological age and chronological age, with skin aging possibly serving as a proxy for systemic oxidative stress (Bobrov et al., 2018; Zhao X et al., 2016).

A 2023 study of nine biomarkers tested the performance of PCA, MLR and KDM to predict biological age, and found that PCA and MLR outperformed KDM-based calculations in terms of correlation with chronological age (Li et al., 2023). In a series of three published studies, Zhang and colleagues use PCA to select different sets of biomarkers to estimate biological age in a Han Chinese population (Zhang et al., 2014a; Zhang et al., 2014b; Zhang et al., 2017). Pyrkov and colleagues examine the relationship of locomotor activity-based metrics with biological aging; they determined that a PCA-based locomotor activity clock outperformed other algorithms trained (Pyrkov et al., 2018a; Pyrkov et al., 2018b). Using the NHANES study participants and a set of 17 selected biomarkers, Rahman and Adjeroh describe an exploration of MLR, KDM and Deep Neural Networks (DNN) in conjunction with centroid- or medoid-based age neighborhood construction, finding that the DNN performed best, with medoid-based estimation performing best for mortality modeling (Rahman and Adjeroh, 2020). A study developed using the Canadian Longitudinal Study on Aging developed six interrelated measures of biological aging, corresponding to different physiological systems, which showed different rates of aging in men and women, and different associations with mortality, multimorbidity, and hospitalization (Verschoor et al., 2021).

Research has also explored the predictive capacity of physical ability on biological age. The functional aging index which is composed of gait speed, subjective sensory ability, muscle strength and lung function, was found to significantly increase risk of hospitalization and mortality, with one study showing an associated mortality risk of 27% per standard deviation of functional aging index-measured biological age (Finkel et al., 2017; Li et al., 2020). That work found that while functional aging index correlated with frailty index, it negatively correlated with cognitive function (Li et al., 2020). The study also associated 32% and 15% increased mortality risk per standard deviation of biological age measured by frailty index and cognitive function respectively. An additional study found that frailty index was a better predictor of mortality than Horvath’s pan-tissue clock (Kim et al., 2017). Using 29 indices of cardiovascular function and testing four different machine learning approaches, Schumann and colleagues found that the Gaussian process regression model outperformed other approaches in biological age and chronological age correlation, and found that cardiovascular changes were associated with obesity in accelerated biological age (Schumann et al., 2023). One paper described the use of fBioAge, which is a composite of grip strength, FEV1, vision and hearing (Sternäng et al., 2019). The study found that the ‘slow aging’ group tended to be older, more likely male, had more schooling, better literacy and fluency, better recall, processing speed, and semantic knowledge. Immunity-based and immune cell proportion clocks have also been developed in recent years, with three papers reporting clocks based on immune system features. The 2021 ImmunoAge clock found an association between anxiety and elevated ImmunoAge in women, while healthy centenarians exhibited a lower ImmunoAge (Martínez de Toda I et al., 2021). That paper also reported a 1 month nutritional intervention resulted in improved ImmunoAge. Using an immunity-based clock in a study of 22 men with clinically stable COPD, it was found that COPD patients have an elevated immune-based age due to a combination of immunosenescence, pro-inflammatory state and oxidative stress (Maté et al., 2021). A 2023 paper describing immune cell proportions in 17 participants found delayed age-related changes in those cell type proportions in supercentenarians; they also observed an association with lower inflammation (Zhu et al., 2023).

Next, we address the novel, often purpose-focused clocks examined for this review. A novel clock based on cardiovascular parameters has been developed using linear regression techniques, finding sex-specific associations with different clinical features and cardiovascular health (Fedintsev et al., 2017). A 2015 paper describes the development of a ‘healthy aging score’ based on expression of 150 genes, where a better score was associated with better renal function in a 12 years follow-up (Sood et al., 2015). Post-mortem brain samples from Alzheimer’s patients exhibited a lower healthy aging score, as did samples from individuals with MCI. Other novel clocks have been developed using elastic net regression, including a metabolomic age clock (Robinson et al., 2020). This clock makes use of serum and urine biomarkers assessed by liquid chromatography-mass spectrometry and nuclear magnetic resonance spectroscopy, was trained on a population of men, correlated with chronological age, and revealed that elevated metabolomic age was associated with being elevated BMI, habitual heavy drinking, presence of diabetes, depressive symptoms/depression, anxiety and PTSD (Robinson et al., 2020). The GlycanAge clock, based on measurements of IgG glycan structures, found 21 glycan structures significantly associated with chronological age, from which the authors developed their clock (Krištić et al., 2014). They found that galactosylation was the most significant age-related glycosylation change, and that men and women exhibit different patterns of change in glycosylation.

Medical imaging has also been explored for novel clock development. The chest radiograph based ‘CXR-Age’ clock is associated with baseline cardiovascular disease risk factors including male sex, diabetes or hypertension diagnoses, obesity, and smoking status (Raghu et al., 2021). Two retinal imaging based clocks have been developed: 2022s ‘RetiAge’ clock associated with all-cause mortality, cardiovascular and cancer mortality, while a novel clock based on retinal microvasculature published in the same year finds that this non-invasive data source be informative for biological aging and cardiovascular disease risk (Nusinovici et al., 2022; Shokr et al., 2022).

Finally, we address deep learning-based clocks, trained on a variety of input data including blood biomarkers, physical behavior, and facial appearance (Mamoshina et al., 2018; Mamoshina et al., 2019; Rahman and Adjeroh, 2019; Rahman and Adjeroh, 2020; Xia et al., 2020). A pair of studies demonstrated that deep learning-based methodologies are highly competitive with other machine learning approaches, finding that with a large training set, deep neural networks could achieve comparable correlations and MAE (Mamoshina et al., 2018; Mamoshina et al., 2019). Training DNNs on three genetically distinct populations revealed that models performed best on the populations they were trained on, implying that there were population-specific significances associated with the blood-based biomarkers features employed (Mamoshina et al., 2018). A DNN trained on non-smokers’ blood-derived biomarkers measured accelerated aging in a smoking test population (Mamoshina et al., 2019). A DNN clock based on 36 markers with a MAE of 6.0 years demonstrated improvements in measured biological age following adherence to either the Mediterranean or DASH diets (Esposito et al., 2022). A similarly accurate DNN-based clock developed using the Moli-Sani population found that elevated biological age was associated with all-cause mortality, hospitalization, cardiovascular disease, ischemic heart disease and cerebrovascular disease (Gialluisi et al., 2022). A second clock developed from the same study population determined that elevated dietary inflammatory index resulted in increased biological age (Martínez et al., 2023). A DNN clock based on 16 biomarkers and exhibiting a MAE of 5.68 years, revealed an association of stroke history, liver disease and lung conditions, but not heart disease or cancer history, with elevated biological age (Galkin et al., 2022). This clock also revealed that smoking, marital status, and sleep quality were key factors in influencing biological age. Using a combination of chronological age and locomotor activity collected from the NHANES data set, applied to a convolutional long short-term memory (ConvLSTM) neural network, this network could predict biological age more accurately than other network structures tested (Rahman and Adjeroh, 2019). A study employing a back propagation artificial neural network (BP-ANN) to develop a biological aging clock achieved a moderate correlation with chronological age, and found that a diet rich in both polyphenols and proboiotics led to a decrease in biological age acceleration (Meng et al., 2023). Lastly, the CNNPerceivedAge CNN can determine chronological and perceived age from a photo, and associate health parameters such as BMI, blood pressure, and clinical blood biomarkers with that photo (Xia et al., 2020).

## 4 Discussion

**FIGURE 4:**
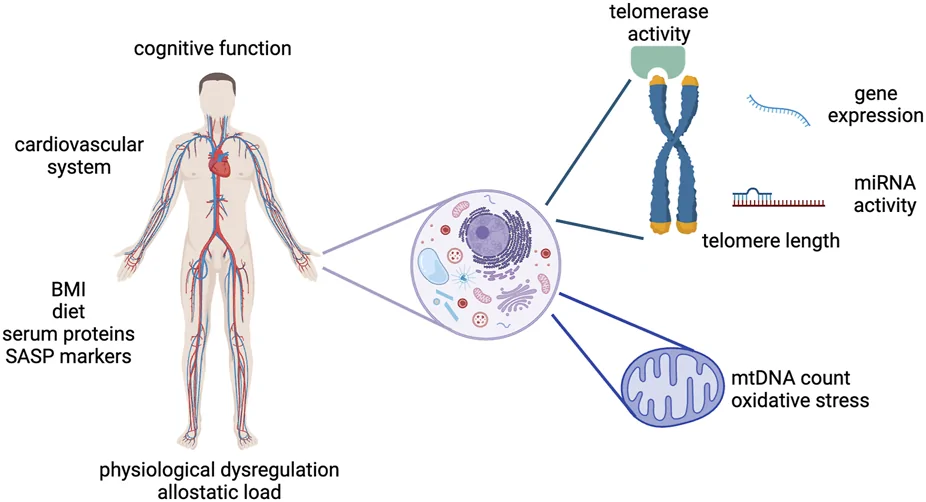
Summary of the findings presented in this review, including data pertaining to the organismal, cellular, and subcellular levels. SASP - senescence associated secretory phenotype/proteins. Figure created with BioRender.com.

The Results section above reveals a map of the available domain knowledge for non-methylation-based measures of biological age, and for the associated algorithms for those measures. By focusing on major features of biological age measures such as telomere length attrition, gene expression patterns, mitochondrial DNA abundance and others, along with specific measurement algorithms, we make possible comparisons between methodologies; Figure 4 reveals the relationships between these features. In contrast, what this review does not examine is the epigenetic methods of biological age measurement. We have recently published a companion review article that correspondingly focuses on methylation-based measures of biological age (Ziesel et al., 2025). For a comparison of these two broad categories, we direct readers to the combined version of these reviews (Ziesel et al., 2024).

Biological aging research has undergone incredible progress, with this review article recounting many of the novel measurement strategies employed to describe the aging process. This research has led to the formulation of new hypotheses, while leaving some longstanding questions unsatisfactorily answered. By making use of the scoping review methodology we have identified current knowledge gaps and broken identified gaps into five distinct groupings: questions regarding measurement, issues pertaining to factors outside the organism including infectious disease, questions regarding the biological aging process, questions of group differences including sex- and ethnicity-based distinctions and general biomarker-based measurement questions.

### 4.1 Measurement questions

Some of the reviewed works observe that different biological age measures such as telomere length, mitochondrial DNA copy number, methylation, or blood-based biomarkers do not always concur. Is there a direct relationship between these different measurements and if so, what is the nature of that relationship? What biological processes might underlie these very different molecular features? Indirectly related to this question is the characterization of different markers of biological age as responsive to aging, versus generative of the aging phenotype. Differentiating between these two feature types would greatly facilitate intervention and diagnostic efforts.

The influence of different environmental or health conditions on biological aging has yet to be fully characterized. It is unclear whether these conditions are influencing biological aging rates or the measurement of biological aging. This relates to the responsive versus generative dichotomy of biological age markers, as conditions that alter aging generative markers would authentically modify biological aging trajectories, while those that influence responsive markers could be reflective only of changes in biological age measurement.

A more specific question regarding measurement of biological age focuses on the use of medical imaging to assess changes in internal organs. Do these approaches overcome the difficulty of directly sampling those tissues, or are volumetric and morphological changes not sufficiently specific to the process of biological aging? Which changes are informative, while which are less specific?

### 4.2 Biomarker-based measurement questions

Many of the biological aging clocks reviewed here make use of blood-based biomarkers. Why do some biomarkers exhibit significant sex-based differences in relation to biological aging? Do these reflect a difference in biological aging rates between the sexes? A number of biomarker-based studies are done in Caucasian and Asian populations but with one exception to be discussed shortly, we have not found any systematic research comparing biomarkers between ethnicities in the time period reviewed here. Are blood-based biomarkers ethnicity-dependent?

### 4.3 Other measurement questions

One paper compared biomarker-based ethnic differences between three distinct groups (Mamoshina et al., 2018). This paper employed a deep learning-based approach to assessing biological age in large populations, generating distinct models trained on each of the groups considered, and then tested on non-training data. An additional paper used deep learning trained on facial photographs to assess biological aging (Xia et al., 2020). While neural network and deep learning approaches are undoubtedly powerful, are they at risk of learning non-aging features such as underlying ethnic differences? Beyond sex- and ethnicity-based differences, there are further potential confounding factors that may stymie the development of a so-called universal biological aging clock such as socioeconomic status or lifestyle differences. Given these factors, is a pan-ethnicity biological clock genuinely possible, or will different sub-groups respond more accurately to bespoke measurements?

Within larger ethnic groups, different patterns of biological aging are also observed. In the work of von Kanel and colleagues, it was repeatedly found that Black South African teachers displayed shorter telomeres than their Caucasian colleagues, contrary to findings in American populations (von Känel et al., 2014; von Känel et al., 2015). What underlies these differences in observations? Do other markers of biological aging in South Africans diverge from patterns observed in Americans? Investigating these differences may help to elucidate non-biological factor influence on patterns of biological aging.

### 4.4 Extra-organismal variables

While many papers considered the impact of chronic disease on the biological aging rate, we found relatively few papers that looked at infectious chronic disease. Those works were limited to examinations of the influence of HIV infection on the aging process, associations with CMV, as well as early work on the impact of COVID-19. A characterization of the influence of other chronic infectious conditions such as hepatitis on the biological aging process would be welcome, as would work on endemic viruses including Epstein-Barr virus. Further, are there differences in the influence of untreated bacterial, fungal or parasitic infections versus viral infections on the biological aging process in affected individuals? Are individuals of advanced biological age more susceptible to chronic infection to begin with? When during the time frame of a chronic infection does impact to biological aging occur?

### 4.5 Biological age variables

Modifiable lifestyle and environmental variables are almost certain to contribute to biological aging rates in individuals and populations. Are these modifiable variables contributors to biological aging, or are they covariates with other factors, such as socioeconomic factors, that are the primary contributors to the biological aging process?

Work has been performed to investigate the impacts of critical early life events on biological age decades later in life. When those early life events were found to have an impact on biological age, it is unclear whether the effect was acute or delayed, leading either to a lifelong acceleration in aging or a deferred impact to biological age. Regardless, the identification of specific features to assess those early life impacts to biological aging would be invaluable.

While telomere length has been explored in a number of model organisms including non-vertebrate organisms, not all measures of biological aging have been characterized in model organisms. Are certain biological aging markers human-specific, primate-specific, mammalian-specific, or general animal features of biological aging? The answer to this expansive question will greatly influence which animal models are most relevant to the study of human aging.

### 4.6 Limitations

While we have undertaken to present the available relevant data on biological aging in a fair and object manner, there are certain shortcomings in our approach. Firstly, our attempt to cover a relatively long period of time means that each individual work during that time period can only be described in sketches and key findings. The selection of employed inclusion and exclusion criteria may have overlooked relevant work that occurred during our review period. Our decision to split our review work into two companion reviews, one focusing on methylation-based biomarkers of biological aging, and this review, addressing all other biomarkers, means that it is difficult to synthesize knowledge across this chasm; this decision was made to prevent a single review from being unreasonably lengthy. Publication bias, in which negative findings are less likely to be successfully published, means that those findings are at greater risk of being excluded from any review process. While we have attempted to broadly cover as many research disciplines with work into biological aging as possible, certainly some fields have been more richly represented to the detriment of others.

## 5 Conclusion

This review has taken a careful examination of a twelve and a half year window of work pertaining to the study of biological aging, focusing on methodological developments and molecular features. While a great breadth and depth of work has been undertaken, questions remain to be answered, and new testable hypotheses have been generated. Several of these outstanding questions pertain to the universality of a hypothetical biological clock, or whether such a clock is possible. Our current body of knowledge and methodology sets the stage for exciting possibilities in further understanding of the process of biological aging, along with opportunities for early surveillance and intervention.

## References

[B1] Accardi G. Bono F. Cammarata G. Aiello A. Herrero M. T. Alessandro R. (2022). miR-126-3p and miR-21-5p as hallmarks of bio-positive ageing; correlation analysis and machine learning prediction in young to ultra-centenarian Sicilian population. Cells 11, 1505. 10.3390/cells11091505 PMC9099697 35563810

[B2] Aguiar S. S. Rosa T. S. Sousa C. V. Santos P. A. Barbosa L. P. Deus L. A. (2021). Influence of body fat on oxidative stress and telomere length of master athletes. J. Strength & Cond. Res. (Lippincott Williams & Wilkins) 35, 1693–1699. 10.1519/jsc.0000000000002932 30640301

[B3] Ahola K. Sirén I. Kivimäki M. Ripatti S. Aromaa A. Lönnqvist J. (2012). Work-related exhaustion and telomere length: a population-based study. PLoS One 7, e40186. 10.1371/journal.pone.0040186 22808115 PMC3394788

[B4] Al-Daghri N. M. Abdi S. Sabico S. Alnaami A. M. Wani K. A. Ansari M. G. A. (2021). Gut-derived endotoxin and telomere length attrition in adults with and without type 2 diabetes. Biomolecules 11. 1693, 10.3390/biom11111693 34827691 PMC8615790

[B5] Alonso-Pedrero L. Donat-Vargas C. Bes-Rastrollo M. Ojeda-Rodríguez A. Zalba G. Razquin C. (2022). Dietary exposure to polychlorinated biphenyls and dioxins and ts relationship to telomere length in subjects older than 55 years from the SUN project. Nutrients 14. 353, 10.3390/nu14020353 PMC8779661 35057533

[B6] Amaro-Gahete F. J. De-la-O A Jurado-Fasoli L. Ruiz J. R. Castillo M. J. (2019). Association of basal metabolic rate and fuel oxidation in basal conditions and during exercise, with plasma S-klotho: the FIT-AGEING study. Aging (Albany NY) 11, 5319–5333. 10.18632/aging.102100 31390595 PMC6710061

[B7] Amgalan A. Maher A. S. Ghosh S. Chui H. C. Bogdan P. Irimia A. (2022). Brain age estimation reveals older adults’ accelerated senescence after traumatic brain injury. Geroscience 44, 2509–2525. 10.1007/s11357-022-00597-1 PMC9768106 35792961

[B8] An R. Yan H. (2017). Body weight status and telomere length in U.S. middle-aged and older adults. Obes. Res. Clin. Pract. 11, 51–62. 10.1016/j.orcp.2016.01.003 26895795

[B9] Arksey H. O’Malley L. (2005). Scoping studies: towards a methodological framework. Int. J. Fo Soc. Res. Methodol. 8, 19–32. 10.1080/1364557032000119616

[B10] Arts M. H. L. Collard R. M. Comijs H. C. de Jonge L. Penninx BWJH Naarding P. (2018). Leucocyte telomere length is no molecular marker of physical frailty in late-life depression. Exp. Gerontol. 111, 229–234. 10.1016/j.exger.2018.07.016 30071286

[B11] Bae C. Y. Kang Y. G. Suh Y. S. Han J. H. Kim S. S. Shim K. W. (2013). A model for estimating body shape biological age based on clinical parameters associated with body composition. Clin. Interv. Aging 8, 11–18. 10.2147/CIA.S38220 23293513 PMC3534388

[B12] Bahour N. Cortez B. Pan H. Shah H. Doria A. Aguayo-Mazzucato C. (2022). Diabetes mellitus correlates with increased biological age as indicated by clinical biomarkers. Geroscience 44, 415–427. 10.1007/s11357-021-00469-0 34773197 PMC8589453

[B13] Banszerus V. L. Vetter V. M. Salewsky B. König M. Demuth I. (2019). Exploring the relationship of relative telomere length and the epigenetic clock in the LipidCardio cohort. Int. J. Mol. Sci. 20, 3032. 10.3390/ijms20123032 31234328 PMC6628615

[B14] Baranyi G. Deary I. J. McCartney D. L. Harris S. E. Shortt N. Reis S. (2022). Life-course exposure to air pollution and biological ageing in the Lothian Birth Cohort 1936. Environ. Int. 169, 107501. 10.1016/j.envint.2022.107501 36126422

[B15] Barha C. K. Hanna C. W. Salvante K. G. Wilson S. L. Robinson W. P. Altman R. M. (2016). Number of children and telomere length in women: a prospective, longitudinal evaluation. PLoS One 11, e0146424. 10.1371/journal.pone.0146424 26731744 PMC4701185

[B16] Barha C. K. Salvante K. G. Hanna C. W. Wilson S. L. Robinson W. P. Altman R. M. (2017). Child mortality, hypothalamic-pituitary-adrenal axis activity and cellular aging in mothers. PLoS One 12, e0177869. 10.1371/journal.pone.0177869 28542264 PMC5444612

[B17] Barzilai N. (2017). Targeting aging with metformin (tame). Innovation Aging 1, 743. 10.1093/geroni/igx004.2682

[B18] Bauer H. Nowak D. Herbig B. (2020). Age, aging and physiological dysregulation in safety-critical work: a retrospective longitudinal study of helicopter emergency medical services pilots. Int. Arch. Occup. Environ. Health 93, 301–314. 10.1007/s00420-019-01482-9 31696315

[B19] Beam C. R. Turkheimer E. Finkel D. Levine M. E. Zandi E. Guterbock T. M. (2020). Midlife study of the Louisville twins: connecting cognitive development to biological and cognitive aging. Behav. Genet. 50, 73–83. 10.1007/s10519-019-09983-6 31820295 PMC7033012

[B20] Belsky D. W. Caspi A. Houts R. Cohen H. J. Corcoran D. L. Danese A. (2015). Quantification of biological aging in young adults. Proc. Natl. Acad. Sci. U. S. A. 112, E4104–E4110. 10.1073/pnas.1506264112 26150497 PMC4522793

[B21] Belsky D. W. Caspi A. Cohen H. J. Kraus W. E. Ramrakha S. Poulton R. (2017). Impact of early personal-history characteristics on the pace of aging: implications for clinical trials of therapies to slow aging and extend healthspan. Aging Cell 16, 644–651. 10.1111/acel.12591 28401731 PMC5506399

[B22] Belsky D. W. Huffman K. M. Pieper C. F. Shalev I. Kraus W. E. (2018a). Change in the rate of biological aging in response to caloric restriction: CALERIE biobank analysis. Journals Gerontology Ser. A Biol. Sci. & Med. Sci. 73, 4–10. 10.1093/gerona/glx096 28531269 PMC5861848

[B23] Belsky D. W. Moffitt T. E. Cohen A. A. Corcoran D. L. Levine M. E. Prinz J. A. (2018b). Eleven telomere, epigenetic clock, and biomarker-composite quantifications of biological aging: do they measure the same thing? Am. J. Epidemiol. 187, 1220–1230. 10.1093/aje/kwx346 29149257 PMC6248475

[B24] Belsky D. W. Caspi A. Arseneault L. Baccarelli A. Corcoran D. L. Gao X. (2020). Quantification of the pace of biological aging in humans through a blood test, the DunedinPoAm DNA methylation algorithm. Elife 9, e54870. 10.7554/eLife.54870 32367804 PMC7282814

[B25] Berben L. Antoranz A. Kenis C. Smeets A. Vos H. Neven P. (2021). Blood immunosenescence signatures reflecting age, frailty and tumor immune infiltrate in patients with early luminal breast cancer. Cancers 13, 2185. 10.3390/cancers13092185 PMC8125302 34063210

[B26] Berdyshev G. D. Korotaev G. K. Boiarskikh G. V. Vaniushin B. F. (1967). Nucleotide composition of DNA and RNA from somatic tissues of humpback and its changes during spawning. Biokhimiia Mosc. Russ. 32, 988–993. 5628601

[B27] Bobrov E. Georgievskaya A. Kiselev K. Sevastopolsky A. Zhavoronkov A. Gurov S. (2018). PhotoAgeClock: deep learning algorithms for development of non-invasive visual biomarkers of aging. Aging (Albany NY) 10, 3249–3259. 10.18632/aging.101629 30414596 PMC6286834

[B28] Bocklandt S. Lin W. Sehl M. E. Sánchez F. J. Sinsheimer J. S. Horvath S. (2011). Epigenetic predictor of age. PLoS One 6, e14821. 10.1371/journal.pone.0014821 21731603 PMC3120753

[B29] Borghini A. Giardini G. Tonacci A. Mastorci F. Mercuri A. Mrakic-Sposta S. (2015). Chronic and acute effects of endurance training on telomere length. Mutagenesis 30, 711–716. 10.1093/mutage/gev038 26001753

[B30] Boström A. D. E. Andersson P. Chatzittofis A. Savard J. Rask-Andersen M. Öberg K. G. (2022). HPA-axis dysregulation is not associated with accelerated epigenetic aging in patients with hypersexual disorder. Psychoneuroendocrinology 141. 105765, 10.1016/j.psyneuen.2022.105765 35452872

[B31] Bountziouka V. Nelson C. P. Codd V. Wang Q. Musicha C. Allara E. (2022). Association of shorter leucocyte telomere length with risk of frailty. J. Cachexia Sarcopenia Muscle 13, 1741–1751. 10.1002/jcsm.12971 35297226 PMC9178164

[B32] Bourassa K. J. Caspi A. Harrington H. Houts R. Poulton R. Ramrakha S. (2020). Intimate partner violence and lower relationship quality are associated with faster biological aging. Psychol. Aging 35, 1127–1139. 10.1037/pag0000581 33211513 PMC7712579

[B33] Bourassa K. J. Moffitt T. E. Ambler A. Hariri A. R. Harrington H. Houts R. M. (2022). Association of treatable health conditions during adolescence with accelerated aging at midlife. JAMA Pediatr. 176, 392–399. 10.1001/jamapediatrics.2021.6417 PMC8861897 35188538

[B34] Brouwers B. Dalmasso B. Hatse S. Laenen A. Kenis C. Swerts E. (2015). Biological ageing and frailty markers in breast cancer patients. Aging (Albany NY) 7, 319–333. 10.18632/aging.100745 25989735 PMC4468313

[B35] Brouwers B. Hatse S. Dal Lago L. Neven P. Vuylsteke P. Dalmasso B. (2016). The impact of adjuvant chemotherapy in older breast cancer patients on clinical and biological aging parameters. Oncotarget 7, 29977–29988. 10.18632/oncotarget.8796 27102154 PMC5058657

[B36] Brunt V. E. Ikoba A. P. Ziemba B. P. Ballak D. B. Hoischen A. Dinarello C. A. (2023). Circulating interleukin-37 declines with aging in healthy humans: relations to healthspan indicators and IL37 gene SNPs. Geroscience 45, 65–84. 10.1007/s11357-022-00587-3 35622271 PMC9137444

[B37] Bürkle A. Moreno-Villanueva M. Bernhard J. Blasco M. Zondag G. Hoeijmakers J. H. (2015). Mark-age biomarkers of ageing. Mech. Ageing Dev. 151, 2–12. 10.1016/j.mad.2015.03.006 25818235

[B38] Cabrera-Mendoza B. Stertz L. Najera K. Selvaraj S. Teixeira A. L. Meyer T. D. (2023). Within subject cross-tissue analyzes of epigenetic clocks in substance use disorder postmortem brain and blood. Am. J. Med. Genet. Part B Neuropsychiatric Genet. 192, 13–27. 10.1002/ajmg.b.32920 PMC9742183 36056652

[B39] Cao W. Zheng D. Wang G. Zhang J. Ge S. Singh M. (2020). Modelling biological age based on plasma peptides in Han Chinese adults. Aging (Albany NY) 12, 10676–10686. 10.18632/aging.103286 32501290 PMC7346055

[B40] Carroll J. E. Diez-Roux A. V. Adler N. E. Seeman T. E. (2013). Socioeconomic factors and leukocyte telomere length in a multi-ethnic sample: findings from the multi-ethnic study of atherosclerosis (MESA). Brain Behav. Immun. 28, 108–114. 10.1016/j.bbi.2012.10.024 23142704 PMC3544984

[B41] Carroll J. E. Irwin M. R. Seeman T. E. Diez-Roux A. V. Prather A. A. Olmstead R. (2019a). Obstructive sleep apnea, nighttime arousals, and leukocyte telomere length: the Multi-ethnic Study of Atherosclerosis. Sleep 42, zsz089. 10.1093/sleep/zsz089 30994174 PMC6612669

[B42] Carroll J. E. Van Dyk K. Bower J. E. Scuric Z. Petersen L. Schiestl R. (2019b). Cognitive performance in survivors of breast cancer and markers of biological aging. Cancer 125, 298–306. 10.1002/cncr.31777 30474160 PMC7082843

[B43] Carroll J. E. Price J. E. Brown J. Bamishigbin O. Shalowitz M. U. Ramey S. (2022). Lifetime discrimination in low to middle income mothers and cellular aging: a prospective analysis. Soc. Sci. & Med. 311, 1–7. 10.1016/j.socscimed.2022.115356 36122526 PMC10024938

[B44] Carulli L. Anzivino C. Baldelli E. Zenobii M. F. Rocchi M. B. Bertolotti M. (2016). Telomere length elongation after weight loss intervention in obese adults. Mol. Genet. Metab. 118, 138–142. 10.1016/j.ymgme.2016.04.003 27157420

[B45] Chae D. H. Epel E. S. Nuru-Jeter A. M. Lincoln K. D. Taylor R. J. Lin J. (2016). Discrimination, mental health, and leukocyte telomere length among African American men. Psychoneuroendocrinology 63, 10–16. 10.1016/j.psyneuen.2015.09.001 26398001 PMC5407686

[B46] Chen L. Shivappa N. Dong X. Ming J. Zhao Q. Xu H. (2021). Association between appendicular skeletal muscle index and leukocyte telomere length in adults: a study from National Health and Nutrition Examination Survey (NHANES) 1999–2002. Clin. Nutr. 40, 3470–3478. 10.1016/j.clnu.2020.11.031 33309414

[B47] Chen L. Zhang Y. Yu C. Guo Y. Sun D. Pang Y. (2023). Modeling biological age using blood biomarkers and physical measurements in Chinese adults. EBioMedicine 89, 104458. 10.1016/j.ebiom.2023.104458 36758480 PMC9941058

[B48] Cheng F. Luk A. O. Tam C. H. T. Fan B. Wu H. Yang A. (2020). Shortened relative leukocyte telomere length is associated with prevalent and incident cardiovascular complications in type 2 diabetes: analysis from the Hong Kong diabetes register. Diabetes Care 43, 2257–2265. 10.2337/dc20-0028 32661111

[B49] Chmelar C. Jörres R. A. Kronseder A. Müller A. Nowak D. Weigl M. (2017). Associations between age, psychosocial work conditions, occupational well-Being, and telomere length in geriatric care professionals: a mixed-methods Study. J. Occup. Environ. Med. 59, 949–955. 10.1097/JOM.0000000000001102 28697059

[B50] Christensen M. W. Keefe D. L. Wang F. Hansen C. S. Chamani I. J. Sommer C. (2021). Idiopathic early ovarian aging: is there a relation with premenopausal accelerated biological aging in young women with diminished response to ART? J. Assist. Reprod. Genet. 38, 3027–3038. 10.1007/s10815-021-02326-7 PMC8609087 34599460

[B51] Cole J. H. Ritchie S. J. Bastin M. E. Valdés Hernández M. C. Muñoz Maniega S. Royle N. (2018). Brain age predicts mortality. Mol. Psychiatry 23, 1385–1392. 10.1038/mp.2017.62 28439103 PMC5984097

[B52] Contini C. Serrao S. Manconi B. Olianas A. Iavarone F. Bizzarro A. (2022). Salivary proteomics reveals significant changes in relation to alzheimer’s disease and aging. J. Alzheimers Dis. 89, 605–622. 10.3233/JAD-220246 35912740

[B53] Costa P. T. McCrae R. R. (1980). Functional age: a conceptual and empirical critique. Epidemiol. Aging 80, 969.

[B54] Couoh L. R. (2017). Differences between biological and chronological age-at-death in human skeletal remains: a change of perspective. Am. J. Phys. Anthropol. 163, 671–695. 10.1002/ajpa.23236 28467628

[B55] Covidence (2024). Covidence - Better Systematic Review Management.

[B56] Cree L. M. Patel S. K. Pyle A. Lynn S. Turnbull D. M. Chinnery P. F. (2008). Age-related decline in mitochondrial DNA copy number in isolated human pancreatic islets. Diabetologia 51, 1440–1443. 10.1007/s00125-008-1054-4 18528676

[B57] Crimmins E. M. (2020). Social hallmarks of aging: suggestions for geroscience research. Ageing Res. Rev. 63, 101136. 10.1016/j.arr.2020.101136 32798771 PMC7530044

[B58] Crimmins E. M. Thyagarajan B. Kim J. K. Weir D. Faul J. (2021). Quest for a summary measure of biological age: the health and retirement study. Geroscience 43, 395–408. 10.1007/s11357-021-00325-1 PMC8050146 33544281

[B59] Crowe C. L. Domingue B. W. Graf G. H. Keyes K. M. Kwon D. Belsky D. W. (2021). Associations of loneliness and social isolation with health span and life span in the U.S. health and retirement study. Journals Gerontology Ser. A Biol. Sci. & Med. Sci. 76, 1997–2006. 10.1093/gerona/glab128 33963758 PMC8514074

[B60] De Francesco D. Wit F. W. Bürkle A. Oehlke S. Kootstra N. A. Winston A. (2019). Do people living with HIV experience greater age advancement than their HIV-negative counterparts? AIDS 33, 259–268. 10.1097/QAD.0000000000002063 PMC6319574 30325781

[B61] Dean W. Morgan R. F. (1988). In defense of the concept of biological aging measurement - current status. Archives Gerontology Geriatrics 7, 191–210. 10.1016/0167-4943(88)90002-7 3052338

[B62] Denham J. Nelson C. P. O’Brien B. J. Nankervis S. A. Denniff M. Harvey J. T. (2013). Longer leukocyte telomeres are associated with ultra-endurance exercise independent of cardiovascular risk factors. PLoS One 8, e69377. 10.1371/journal.pone.0069377 23936000 PMC3729964

[B63] Denham J. O’Brien B. J. Prestes P. R. Brown N. J. Charchar F. J. (2016). Increased expression of telomere-regulating genes in endurance athletes with long leukocyte telomeres. J. Appl. Physiol. (1985) 120, 148–158. 10.1152/japplphysiol.00587.2015 26586905

[B64] Dhillon V. S. Deo P. Chua A. Thomas P. Fenech M. (2020). Shorter telomere length in carriers of APOE–*ɛ*4 and high plasma concentration of glucose, glyoxal and other advanced glycation end products (AGEs). J. Gerontol. A Biol. Sci. Med. Sci. 75, 1894–1898. 10.1093/gerona/glz203 31541246

[B65] Dirken J. M. (1972). *Functional Age of Industrial Workers: A Transversal Survey of Ageing Capacities and a Method for Assessing Functional Age* (Groningen: Netherlands Institute for Preventative Medicine TNO: Wolters-Noordhoff).

[B66] Dmitrieva N. I. Gagarin A. Liu D. Wu C. O. Boehm M. (2023). Middle-age high normal serum sodium as a risk factor for accelerated biological aging, chronic diseases, and premature mortality. EBioMedicine 87, 104404. 10.1016/j.ebiom.2022.104404 36599719 PMC9873684

[B67] Dolcini J. Wu H. Nwanaji-Enwerem J. C. Kiomourtozlogu M. A. Cayir A. Sanchez-Guerra M. (2020). Mitochondria and aging in older individuals: an analysis of DNA methylation age metrics, leukocyte telomere length, and mitochondrial DNA copy number in the VA normative aging study. Aging (Albany NY) 12, 2070–2083. 10.18632/aging.102722 32009007 PMC7041780

[B68] Donze S. H. Codd V. Damen L. Goedegebuure W. J. Denniff M. Samani N. J. (2020). Evidence for accelerated biological aging in young adults with Prader-Willi syndrome. J. Clin. Endocrinol. Metab. 105, 2053–2059. 10.1210/clinem/dgz180 31689713 PMC7150612

[B69] Drewelies J. Hueluer G. Duezel S. Vetter V. M. Pawelec G. Steinhagen-Thiessen E. (2022). Using blood test parameters to define biological age among older adults: association with morbidity and mortality independent of chronological age validated in two separate birth cohorts. Geroscience 44, 2685–2699. 10.1007/s11357-022-00662-9 36151431 PMC9768057

[B70] Dugué P. A. Hodge A. M. Ulvik A. Ueland P. M. Midttun Ø. Rinaldi S. (2022). Association of markers of inflammation, the kynurenine pathway and B vitamins with age and mortality, and a signature of inflammaging. J. Gerontol. A Biol. Sci. Med. Sci. 77, 826–836. 10.1093/gerona/glab163 34117761

[B71] Dunston C. R. Choudhury K. Griffiths H. R. (2012). Terminal galactose residues on transferrin are increased in midlife adults compared to young adults. Proteomics 12, 3147–3153. 10.1002/pmic.201100506 22930475

[B72] Earls J. C. Rappaport N. Heath L. Wilmanski T. Magis A. T. Schork N. J. (2019). Multi-omic biological age estimation and its correlation with wellness and disease phenotypes: a longitudinal study of 3,558 individuals. J. Gerontol. A Biol. Sci. Med. Sci. 74, S52–S60. 10.1093/gerona/glz220 31724055 PMC6853785

[B73] Enroth S. Enroth S. B. Johansson Å. Gyllensten U. (2015). Protein profiling reveals consequences of lifestyle choices on predicted biological aging. Sci. Rep. 5, 17282. 10.1038/srep17282 26619799 PMC4664859

[B74] Epel E. S. Puterman E. Lin J. Blackburn E. Lazaro A. Mendes W. B. (2013). Wandering minds and aging cells. Clin. Psychol. Sci. 1, 75–83. 10.1177/2167702612460234

[B75] Erdmann N. J. Harrington L. A. Martin L. J. (2017). Mammographic density, blood telomere length and lipid peroxidation. Sci. Rep. 7, 5803. 10.1038/s41598-017-06036-y 28725051 PMC5517610

[B76] Esposito S. Gialluisi A. Costanzo S. Di Castelnuovo A. Ruggiero E. De Curtis A. (2021). Dietary polyphenol intake is associated with biological aging, a novel predictor of cardiovascular disease: cross-sectional findings from the moli-sani study. Nutrients 13, 1701. 10.3390/nu13051701 PMC8157169 34067821

[B77] Esposito S. Gialluisi A. Costanzo S. Di Castelnuovo A. Ruggiero E. De Curtis A. (2022). Mediterranean diet and other dietary patterns in association with biological aging in the Moli-sani study cohort. Clin. Nutr. 41, 1025–1033. 10.1016/j.clnu.2022.02.023 35390726

[B78] Fang B. Yan E. Tung K. Liu Z. Ip P. (2021). Association between elder abuse and telomere shortening in older adults: a 2-year prospective study. Int. J. Geriatr. Psychiatry 36, 54–63. 10.1002/gps.5390 32748415

[B79] Farina M. P. Kim J. K. Crimmins E. M. (2023). Racial/ethnic differences in biological aging and their life course socioeconomic determinants: the 2016 health and retirement study. J. Aging & Health 35, 209–220. 10.1177/08982643221120743 35984401 PMC9898094

[B80] Fedintsev A. Kashtanova D. Tkacheva O. Strazhesko I. Kudryavtseva A. Baranova A. (2017). Markers of arterial health could serve as accurate non-invasive predictors of human biological and chronological age. Aging (Albany NY) 9, 1280–1292. 10.18632/aging.101227 28455973 PMC5425127

[B81] Fernandez A. F. Assenov Y. Martin-Subero J. I. Balint B. Siebert R. Taniguchi H. (2012). A DNA methylation fingerprint of 1628 human samples. Genome Res. 22, 407–419. 10.1101/gr.119867.110 21613409 PMC3266047

[B82] Finkel D. Sternäng O. Wahlin Å. (2017). Genetic and environmental influences on longitudinal trajectories of functional biological age: comparisons across gender. Behav. Genet. 47, 375–382. 10.1007/s10519-017-9851-5 28551760 PMC5486850

[B83] Forrester S. N. Whitfield K. E. Kiefe C. I. Thorpe R. J. (2022a). Navigating black aging: the biological consequences of stress and depression. Journals Gerontology Ser. B Psychol. Sci. & Soc. Sci. 77, 2101–2112. 10.1093/geronb/gbab224 PMC9683493 34875069

[B84] Forrester S. N. Zmora R. Schreiner P. J. Jacobs D. R. Jr Roger V. L. Thorpe R. J. Jr (2022b). Racial differences in the association of accelerated aging with future cardiovascular events and all-cause mortality: the coronary artery risk development in young adults study, 2007-2018. Ethn. Health 27, 997–1009. 10.1080/13557858.2020.1839021 33222499 PMC8137718

[B85] Fraszczyk E. Luijten M. Spijkerman A. M. W. Snieder H. Wackers P. F. K. Bloks V. W. (2020). The effects of bariatric surgery on clinical profile, DNA methylation, and ageing in severely obese patients. Clin. Epigenetics 12, 14. 10.1186/s13148-019-0790-2 31959221 PMC6972025

[B86] Furukawa T. Inoue M. Kajiya F. Inada H. Takasugi S. Fukui S. (1975). Assessment of biological age by multiple regression analysis. J. Gerontology 30, 422–434. 10.1093/geronj/30.4.422 1141673

[B87] Galkin F. Kochetov K. Koldasbayeva D. Faria M. Fung H. H. Chen A. X. (2022). Psychological factors substantially contribute to biological aging: evidence from the aging rate in Chinese older adults. Aging (Albany NY) 14, 7206–7222. 10.18632/aging.204264 36170009 PMC9550255

[B88] Gao X. Colicino E. Shen J. Just A. C. Nwanaji-Enwerem J. C. Wang C. (2019). Comparative validation of an epigenetic mortality risk score with three aging biomarkers for predicting mortality risks among older adult males. Int. J. Epidemiol. 48, 1958–1971. 10.1093/ije/dyz082 31038702 PMC6929530

[B89] Gao X. Huang N. Guo X. Huang T. (2022). Role of sleep quality in the acceleration of biological aging and its potential for preventive interaction on air pollution insults: findings from the UK Biobank cohort. Aging Cell 21, e13610. 10.1111/acel.13610 PMC9124313 35421261

[B90] Gaydosh L. Belsky D. W. Glei D. A. Goldman N. (2020). Testing proposed quantifications of biological aging in Taiwanese older adults. J. Gerontol. A Biol. Sci. Med. Sci. 75, 1680–1685. 10.1093/gerona/glz223 31566204 PMC7494028

[B91] Geronimus A. T. Pearson J. A. Linnenbringer E. Schulz A. J. Reyes A. G. Epel E. S. (2015). Race-ethnicity, poverty, urban stressors, and telomere length in a Detroit community-based sample. J. Health Soc. Behav. 56, 199–224. 10.1177/0022146515582100 25930147 PMC4621968

[B92] Gheorghe M. Snoeck M. Emmerich M. Bäck T. Goeman J. J. Raz V. (2014). Major aging-associated RNA expressions change at two distinct age-positions. BMC Genomics 15, 132. 10.1186/1471-2164-15-132 24524210 PMC3930826

[B93] Gialluisi A. Di Castelnuovo A. Costanzo S. Bonaccio M. Persichillo M. Magnacca S. (2022). Exploring domains, clinical implications and environmental associations of a deep learning marker of biological ageing. Eur. J. Epidemiol. 37, 35–48. 10.1007/s10654-021-00797-7 34453631

[B94] Glei D. A. Goldman N. Weinstein M. Risques R. A. (2015). Shorter ends, faster end? Leukocyte telomere length and mortality among older Taiwanese. J. Gerontol. A Biol. Sci. Med. Sci. 70, 1490–1498. 10.1093/gerona/glu191 25326284 PMC4751225

[B95] Gołab S. Woronkowicz A. Kryst Ł. (2016). Biological aging and physical fitness in men aged 20-70 years from Kraków, Poland. Am. J. Hum. Biol. 28, 503–509. 10.1002/ajhb.22820 27416874

[B96] Gong H. Yu Q. Yuan M. Jiang Y. Wang J. Huang P. (2022). The relationship between dietary copper intake and telomere length in hypertension. J. Nutr. Health & Aging 26, 510–514. 10.1007/s12603-022-1787-7 PMC12879115 35587764

[B97] Gorelik S. G. Belousova O. N. Treneva E. V. Bulgakova S. V. Zakharova N. O. Nesterenko S. A. (2022). Effect of daily rhythms of cortisol secretion on the rate of aging in men. Arch. Razi Inst. 77, 1233–1239. 10.22092/ARI.2022.357394.2034 36618277 PMC9759242

[B98] Graf G. H. Crowe C. L. Kothari M. Kwon D. Manly J. J. Turney I. C. (2022). Testing black-white disparities in biological aging among older adults in the United States: analysis of DNA-Methylation and blood-chemistry methods. Am. J. Epidemiol. 191, 613–625. 10.1093/aje/kwab281 PMC9077113 34850809

[B99] Guidi J. Lucente M. Sonino N. Fava G. A. (2020). Allostatic load and its impact on health: a systematic review. Psychotherapy Psychosomatics 90, 11–27. 10.1159/000510696 32799204

[B100] Habib R. Ocklenburg S. Hoffjan S. Haghikia A. Epplen J. T. Arning L. (2020). Association between shorter leukocyte telomeres and multiple sclerosis. J. Neuroimmunol. 341, 577187. 10.1016/j.jneuroim.2020.577187 32050150

[B101] Hammadah M. Al Mheid I. Wilmot K. Ramadan R. Abdelhadi N. Alkhoder A. (2017). Telomere shortening, regenerative capacity, and cardiovascular outcomes. Circ. Res. 120, 1130–1138. 10.1161/CIRCRESAHA.116.309421 27956416 PMC5376244

[B102] Han K. T. Kim D. W. Kim S. J. (2018a). Biological age is associated with the active use of nutrition data. Int. J. Environ. Res. Public Health 15, 2431. 10.3390/ijerph15112431 30388785 PMC6266208

[B103] Han K. T. Kim D. W. Kim S. J. (2018b). Is sleep duration associated with biological age (BA)? analysis of (2010-2015) South Korean NHANES dataset South Korea. Int. J. Environ. Res. Public Health 15, 2009. 10.3390/ijerph15092009 30223512 PMC6163725

[B104] Hannum G. Guinney J. Zhao L. Zhang L. I. Hughes G. Sadda S. (2013). Genome-wide methylation profiles reveal quantitative views of human aging rates. Mol. Cell 49, 359–367. 10.1016/j.molcel.2012.10.016 23177740 PMC3780611

[B105] Hassett A. L. Epel E. Clauw D. J. Harris R. E. Harte S. E. Kairys A. (2012). Pain is associated with short leukocyte telomere length in women with fibromyalgia. J. Pain 13, 959–969. 10.1016/j.jpain.2012.07.003 23031395

[B106] Hastings W. J. Shalev I. Belsky D. W. (2019). Comparability of biological aging measures in the National Health and Nutrition Examination study, 1999-2002. Psychoneuroendocrinology 106, 171–178. 10.1016/j.psyneuen.2019.03.012 30999227 PMC6599717

[B107] Hastings W. J. Almeida D. M. Shalev I. (2022). Conceptual and analytical overlap between allostatic load and systemic biological aging measures: analyses from the National Survey of Midlife Development in the United States. J. Gerontol. A Biol. Sci. Med. Sci. 77, 1179–1188. 10.1093/gerona/glab187 PMC9159656 34180993

[B108] Hatse S. Brouwers B. Dalmasso B. Laenen A. Kenis C. Schöffski P. (2014). Circulating MicroRNAs as easy-to-measure aging biomarkers in older breast cancer patients: correlation with chronological age but not with fitness/frailty status. PLoS One 9, e110644. 10.1371/journal.pone.0110644 25333486 PMC4204997

[B109] Hautekiet P. Saenen N. D. Martens D. S. Debay M. Van der Heyden J. Nawrot T. S. (2022). A healthy lifestyle is positively associated with mental health and well-being and core markers in ageing. BMC Med. 20, 328. 10.1186/s12916-022-02524-9 PMC9520873 36171556

[B110] Hernando B. Gil-Barrachina M. Tomás-Bort E. Martinez-Navarro I. Collado-Boira E. Hernando C. (2020). The effect of long-term ultra-endurance exercise and SOD2 genotype on telomere shortening with age. J. Appl. Physiol. (1985) 129, 873–879. 10.1152/japplphysiol.00570.2020 32881625

[B111] Hertel J. Friedrich N. Wittfeld K. Pietzner M. Budde K. Van der Auwera S. (2016). Measuring biological age *via* metabonomics: the metabolic age score. J. Proteome Res. 15, 400–410. 10.1021/acs.jproteome.5b00561 26652958

[B112] Ho E. Qualls C. Villareal D. T. (2022). Effect of diet, exercise, or both on biological age and healthy aging in older adults with obesity: secondary analysis of a randomized controlled trial. J. Nutr. Health & Aging 26, 552–557. 10.1007/s12603-022-1812-x PMC9236175 35718862

[B113] Hoen P. W. Rosmalen J. G. Schoevers R. A. Huzen J. van der Harst P. de Jonge P. (2013). Association between anxiety but not depressive disorders and leukocyte telomere length after 2 years of follow-up in a population-based sample. Psychol. Med. 43, 689–697. 10.1017/S0033291712001766 22877856

[B114] Holly A. C. Melzer D. Pilling L. C. Henley W. Hernandez D. G. Singleton A. B. (2013). Towards a gene expression biomarker set for human biological age. Aging Cell 12, 324–326. 10.1111/acel.12044 23311345 PMC4623317

[B115] Hong M. G. Dodig-Crnković T. Chen X. Drobin K. Lee W. Wang Y. (2020). Profiles of histidine-rich glycoprotein associate with age and risk of all-cause mortality. Life Sci. Alliance 3, e202000817. 10.26508/lsa.202000817 32737166 PMC7409555

[B116] Honig L. S. Kang M. S. Schupf N. Lee J. H. Mayeux R. (2012). Association of shorter leukocyte telomere repeat length with dementia and mortality. Arch. Neurol. 69, 1332–1339. 10.1001/archneurol.2012.1541 22825311 PMC3622729

[B117] Honig L. S. Kang M. S. Cheng R. Eckfeldt J. H. Thyagarajan B. Leiendecker-Foster C. (2015). Heritability of telomere length in a study of long-lived families. Neurobiol. Aging 36, 2785–2790. 10.1016/j.neurobiolaging.2015.06.017 26239175 PMC4562863

[B118] Honkonen M. Vääräniemi K. Saijonmaa O. Nyman A. Tikkakoski A. J. Koskela J. (2020). Leukocyte telomere length is inversely associated with arterial wave reflection in 566 normotensive and never-treated hypertensive subjects. Aging (Albany NY) 12, 12376–12392. 10.18632/aging.103459 32575070 PMC7343461

[B119] Horvath S. (2013). DNA methylation age of human tissues and cell types. Genome Biol. 14, 1–20. 10.1186/gb-2013-14-10-r115 24138928 PMC4015143

[B120] Hovatta I. de Mello V. D. Kananen L. Lindström J. Eriksson J. G. Ilanne-Parikka P. (2012). Leukocyte telomere length in the Finnish Diabetes Prevention study. PLoS One 7, e34948. 10.1371/journal.pone.0034948 22493726 PMC3321039

[B121] Huang Z. Liu C. Ruan Y. Guo Y. Sun S. Shi Y. (2021). Dynamics of leukocyte telomere length in adults aged 50 and older: a longitudinal population-based cohort study. Geroscience 43, 645–654. 10.1007/s11357-020-00320-y 33469834 PMC8110630

[B122] Iannarelli N. J. Wade T. J. Dempster K. S. Moore J. MacNeil A. J. O’Leary D. D. (2022). No mediation effect of telomere length or mitochondrial DNA copy number on the Association between adverse childhood experiences (ACEs) and central arterial stiffness. J. Am. Heart Assoc. 11, e026619. 10.1161/JAHA.122.026619 36285820 PMC9673639

[B123] Jee H. Park J. (2017). Selection of an optimal set of biomarkers and comparative analyses of biological age estimation models in Korean females. Arch. Gerontol. Geriatr. 70, 84–91. 10.1016/j.archger.2017.01.005 28110151

[B124] Jee H. Jeon B. H. Kim Y. H. Kim H. K. Choe J. Park J. (2012). Development and application of biological age prediction models with physical fitness and physiological components in Korean adults. Gerontology 58, 344–353. 10.1159/000335738 22433233

[B125] Jeffries A. R. Maroofian R. Salter C. G. Chioza B. A. Cross H. E. Patton M. A. (2019). Growth disrupting mutations in epigenetic regulatory molecules are associated with abnormalities of epigenetic aging. Genome Res. 29, 1057–1066. 10.1101/gr.243584.118 31160375 PMC6633263

[B126] Johnson L. C. Parker K. Aguirre B. F. Nemkov T. G. D’Alessandro A. Johnson S. A. (2019). The plasma metabolome as a predictor of biological aging in humans. Geroscience 41, 895–906. 10.1007/s11357-019-00123-w 31707594 PMC6925078

[B127] Kajantie E. Pietiläinen K. H. Wehkalampi K. Kananen L. Räikkönen K. Rissanen A. (2012). No association between body size at birth and leucocyte telomere length in adult life–evidence from three cohort studies. Int. J. Epidemiol. 41, 1400–1408. 10.1093/ije/dys127 22984146

[B128] Kang Y. G. Suh E. Chun H. Kim S. H. Kim D. K. Bae C. Y. (2017). Models for estimating the metabolic syndrome biological age as the new index for evaluation and management of metabolic syndrome. Clin. Interv. Aging 12, 253–261. 10.2147/CIA.S123316 28203066 PMC5295798

[B129] Kang Y. G. Suh E. Lee J. W. Kim D. W. Cho K. H. Bae C. Y. (2018). Biological age as a health index for mortality and major age-related disease incidence in Koreans: National Health Insurance Service - Health screening 11-year follow-up study. Clin. Interv. Aging 13, 429–436. 10.2147/CIA.S157014 29593385 PMC5865564

[B130] Kankaanpää A. Tolvanen A. Heikkinen A. Kaprio J. Ollikainen M. Sillanpää E. (2022). The role of adolescent lifestyle habits in biological aging: a prospective twin study. Elife 11. e80729, 10.7554/eLife.80729 36345722 PMC9642990

[B131] Karametos I. Tsiboli P. Togousidis I. Hatzoglou C. Giamouzis G. Gourgoulianis K. I. (2019). Chronic obstructive pulmonary disease as a main factor of premature aging. Int. J. Environ. Res. Public Health 16, 540. 10.3390/ijerph16040540 30781849 PMC6406938

[B132] Katz R. Gay E. L. Kuipers A. L. Lee J. H. Honig L. S. Christensen K. (2022). Association of leukocyte telomere length with perceived physical fatigability. Exp. Gerontol. 170, 111988. 10.1016/j.exger.2022.111988 36302456 PMC11467958

[B133] Kemp B. R. Ferraro K. F. (2021). Are biological consequences of childhood exposures detectable in telomere length decades later? J. Gerontol. A Biol. Sci. Med. Sci. 76, 7–14. 10.1093/gerona/glaa019 31956916 PMC8355457

[B134] Khan S. S. Shah S. J. Klyachko E. Baldridge A. S. Eren M. Place A. T. (2017). A null mutation in SERPINE1 protects against biological aging in humans. Sci. Adv. 3, eaao1617. 10.1126/sciadv.aao1617 29152572 PMC5687852

[B135] Kim S. Myers L. Wyckoff J. Cherry K. E. Jazwinski S. M. (2017). The frailty index outperforms DNA methylation age and its derivatives as an indicator of biological age. Geroscience 39, 83–92. 10.1007/s11357-017-9960-3 28299637 PMC5352589

[B136] Kingma E. M. de Jonge P. van der Harst P. Ormel J. Rosmalen J. G. (2012). The association between intelligence and telomere length: a longitudinal population based study. PLoS One 7, e49356. 10.1371/journal.pone.0049356 23166646 PMC3498156

[B137] Klemera P. Doubal S. (2006). A new approach to the concept and computation of biological age. Mech. Ageing Dev. 127, 240–248. 10.1016/j.mad.2005.10.004 16318865

[B138] Kocaman S. A. Çetin M. Durakoğlugil M. E. Erdoğan T. Çanga A. Çiçek Y. (2012). The degree of premature hair graying as an independent risk marker for coronary artery disease: a predictor of biological age rather than chronological age. Anadolu Kardiyol. Derg. 12, 457–463. 10.5152/akd.2012.150 22677402

[B139] Koh A. S. Siau A. Gao F. Chioh F. W. J. Leng S. Zhao X. (2023). Left atrial phasic function in older adults is associated with fibrotic and low-grade inflammatory pathways. Gerontology 69, 47–56. 10.1159/000522632 35316808 PMC9492896

[B140] Kolokoltsev M. Romanova E. Vorozheikin A. Bocharin I. Karuzin K. Martusevich A. (2022). The effect of physical activity on biological age and body composition in 18-19-year-old girls. J. Phys. Educ. & Sport 22, 981–987. 10.7752/jpes.2022.04125

[B141] Krištić J. Vučković F. Menni C. Klarić L. Keser T. Beceheli I. (2014). Glycans are a novel biomarker of chronological and biological ages. J. Gerontol. A Biol. Sci. Med. Sci. 69, 779–789. 10.1093/gerona/glt190 24325898 PMC4049143

[B142] Küffer A. L. O’Donovan A. Burri A. Maercker A. (2016). Posttraumatic stress disorder, adverse childhood events, and buccal cell telomere length in elderly Swiss former indentured child laborers. Front. Psychiatry 7, 147. Publisher: Frontiers Media S.A, 10.3389/fpsyt.2016.00147 PMC5005955 27630582

[B143] Kuo P. L. Schrack J. A. Shardell M. D. Levine M. Moore A. Z. An Y. (2020). A roadmap to build a phenotypic metric of ageing: insights from the Baltimore Longitudinal study of aging. J. Intern Med. 287, 373–394. 10.1111/joim.13024 32107805 PMC7670826

[B144] Kuzawa C. W. Ryan C. P. Adair L. S. Lee N. R. Carba D. B. MacIsaac J. L. (2022). Birth weight and maternal energy status during pregnancy as predictors of epigenetic age acceleration in young adults from metropolitan cebu, Philippines. Epigenetics 17, 1535–1545. 10.1080/15592294.2022.2070105 PMC9586628 35574972

[B145] Langevin S. Caspi A. Barnes J. C. Brennan G. Poulton R. Purdy S. C. (2022). Life-course persistent antisocial behavior and accelerated biological aging in a longitudinal birth cohort. Int. J. Environ. Res. Public Health 19. 14402, 10.3390/ijerph192114402 36361282 PMC9657643

[B146] Le Nguyen K. D. Lin J. Algoe S. B. Brantley M. M. Kim S. L. Brantley J. (2019). Loving-kindness meditation slows biological aging in novices: evidence from a 12-week randomized controlled trial. Psychoneuroendocrinology 108, 20–27. 10.1016/j.psyneuen.2019.05.020 31185369

[B147] Lee M. Martin H. Firpo M. A. Demerath E. W. (2011). Inverse association between adiposity and telomere length: the Fels Longitudinal Study. Am. J. Hum. Biol. 23, 100–106. 10.1002/ajhb.21109 21080476 PMC3245638

[B148] Lee J. Y. Jun N. R. Yoon D. Shin C. Baik I. (2015). Association between dietary patterns in the remote past and telomere length. Eur. J. Clin. Nutr. 69, 1048–1052. 10.1038/ejcn.2015.58 25872911

[B149] Lee D. B. Kim E. S. Neblett E. W. (2017). The link between discrimination and telomere length in African American adults. Health Psychol. 36, 458–467. 10.1037/hea0000450 28425738

[B150] Lee J. Y. Shin C. Baik I. (2017). Longitudinal associations between micronutrient consumption and leukocyte telomere length. J. Hum. Nutr. Diet. 30, 236–243. 10.1111/jhn.12403 27550625

[B151] Lee E. H. Han M. H. Ha J. Park H. H. Koh S. H. Choi S. H. (2020). Relationship between telomere shortening and age in Korean individuals with mild cognitive impairment and Alzheimer’s disease compared to that in healthy controls. Aging (Albany NY) 13, 2089–2100. 10.18632/aging.202206 33323554 PMC7880372

[B152] Levine M. E. (2013). Modeling the rate of senescence: can estimated biological age predict mortality more accurately than chronological age? J. Gerontol. A Biol. Sci. Med. Sci. 68, 667–674. 10.1093/gerona/gls233 23213031 PMC3660119

[B153] Levine M. E. Crimmins E. M. (2014). Evidence of accelerated aging among African Americans and its implications for mortality. Soc. Sci. Med. 118, 27–32. 10.1016/j.socscimed.2014.07.022 25086423 PMC4197001

[B154] Levine M. E. Crimmins E. M. (2018). Is 60 the new 50? Examining changes in biological age over the past two decades. Demography 55, 387–402. 10.1007/s13524-017-0644-5 29511995 PMC5897168

[B155] Levine M. E. Lu A. T. Chen B. H. Hernandez D. G. Singleton A. B. Ferrucci L. (2016). Menopause accelerates biological aging. Proc. Natl. Acad. Sci. U. S. A. 113, 9327–9332. 10.1073/pnas.1604558113 27457926 PMC4995944

[B156] Levine M. E. Lu A. T. Quach A. Chen B. H. Assimes T. L. Bandinelli S. (2018). An epigenetic biomarker of aging for lifespan and healthspan. Aging (Albany NY) 10, 573–591. 10.18632/aging.101414 29676998 PMC5940111

[B157] Levy M. Z. Allsopp R. C. Futcher A. B. Greider C. W. Harley C. B. (1992). Telomere end-replication problem and cell aging. J. Mol. Biol. 225, 951–960. 10.1016/0022-2836(92)90096-3 1613801

[B158] Li X. Ploner A. Wang Y. Magnusson P. K. Reynolds C. Finkel D. (2020). Longitudinal trajectories, correlations and mortality associations of nine biological ages across 20-years follow-up. Elife 9, e51507. 10.7554/eLife.51507 32041686 PMC7012595

[B159] Li Z. Zhang W. Duan Y. Niu Y. He Y. Chen Y. (2023). Biological age models based on a healthy Han Chinese population. Archives Gerontology & Geriatrics 107, 104905, N.PAG–N.PAG. 10.1016/j.archger.2022.104905 36542874

[B160] Lin H. Lunetta K. L. Zhao Q. Rong J. Benjamin E. J. Mendelson M. M. (2017). Transcriptome-wide association study of inflammatory biologic age. Aging (Albany NY) 9, 2288–2301. 10.18632/aging.101321 29135455 PMC5723687

[B161] Lin H. Lunetta K. L. Zhao Q. Mandaviya P. R. Rong J. Benjamin E. J. (2019). Whole blood gene expression associated with clinical biological age. J. Gerontol. A Biol. Sci. Med. Sci. 74, 81–88. 10.1093/gerona/gly164 30010802 PMC6298179

[B162] Lin Y. F. Chen P. Y. Liu H. C. Chen Y. L. Chou W. H. Huang M. C. (2021). Shortened leukocyte telomere length in young adults who use methamphetamine. Transl. Psychiatry 11, 519. 10.1038/s41398-021-01640-z 34628468 PMC8502172

[B163] Lincoln K. D. Lloyd D. A. Nguyen A. W. (2019). Social relationships and salivary telomere length among middle-aged and older African American and white adults. J. Gerontol. B Psychol. Sci. Soc. Sci. 74, 1053–1061. 10.1093/geronb/gbx049 28486613 PMC6703231

[B164] Liu Z. (2021). Development and validation of 2 composite aging measures using routine clinical biomarkers in the Chinese population: analyses from 2 prospective cohort studies. Journals Gerontology Ser. A Biol. Sci. & Med. Sci. 76, 1627–1632. 10.1093/gerona/glaa238 PMC8521780 32946548

[B165] Liu B. Sun Y. Xu G. Snetselaar L. G. Ludewig G. Wallace R. B. (2019). Association between body iron status and leukocyte telomere length, a biomarker of biological aging, in a nationally representative sample of US adults. J. Acad. Nutr. Diet. 119, 617–625. 10.1016/j.jand.2018.09.007 30563782 PMC6548326

[B166] Lu L. Johnman C. McGlynn L. Mackay D. F. Shiels P. G. Pell J. P. (2017). Association between exposure to second-hand smoke and telomere length: cross-sectional study of 1303 non-smokers. Int. J. Epidemiol. 46, 1978–1984. 10.1093/ije/dyx212 29040594

[B167] Lu A. T. Quach A. Wilson J. G. Reiner A. P. Aviv A. Raj K. (2019). DNA methylation GrimAge strongly predicts lifespan and healthspan. Aging (Albany NY) 11, 303–327. 10.18632/aging.101684 30669119 PMC6366976

[B168] Lu W. H. Gonzalez-Bautista E. Guyonnet S. Lucas A. Parini A. Walston J. D. (2023). Plasma inflammation-related biomarkers are associated with intrinsic capacity in community-dwelling older adults. J. Cachexia Sarcopenia Muscle 14, 930–939. 10.1002/jcsm.13163 36660894 PMC10067471

[B169] Lucas T. Woerner J. Pierce J. Granger D. A. Lin J. Epel E. S. (2018). Justice for all? Beliefs about justice for self and others and telomere length in African Americans. Cultur. Divers Ethn. Minor Psychol. 24, 498–509. 10.1037/cdp0000212 30058830 PMC6188832

[B170] Maeda T. Oyama J. I. Sasaki M. Arima T. Makino N. (2011). The correlation between the clinical laboratory data and the telomere length in peripheral blood leukocytes of Japanese female patients with hypertension. J. Nutr. Health Aging 15, 240–244. 10.1007/s12603-010-0137-3 21369674 PMC12879645

[B171] Maeda T. Horiuchi T. Makino N. (2018). Epigenetic status of subtelomere of peripheral leukocytes corresponds to cardiographic parameters with a sex association. Geriatr. Gerontol. Int. 18, 1415–1419. 10.1111/ggi.13472 29978589

[B172] Maeda T. Horiuchi T. Makino N. (2019). The approximate formulas predicting personal somatic telomere length using patient blood test data. Can. J. Physiol. Pharmacol. 97, 1090–1093. 10.1139/cjpp-2019-0262 31340127

[B173] Maeda T. Horiuchi T. Makino N. (2021a). Chromosomal terminal methylation status is associated with gut microbiotic alterations. Mol. Cell Biochem. 476, 157–163. 10.1007/s11010-020-03892-7 32888159

[B174] Maeda T. Horiuchi T. Makino N. (2021b). Telomere shortening velocity of patients administered with hypnotics is accelerated in a gender-differential manner. Can. J. Physiol. Pharmacol. 99, 278–283. 10.1139/cjpp-2020-0291 32687724

[B175] Mamoshina P. Kochetov K. Putin E. Cortese F. Aliper A. Lee W. S. (2018). Population specific biomarkers of human aging: a big data study using South Korean, Canadian, and Eastern european patient populations. J. Gerontol. A Biol. Sci. Med. Sci. 73, 1482–1490. 10.1093/gerona/gly005 29340580 PMC6175034

[B176] Mamoshina P. Kochetov K. Cortese F. Kovalchuk A. Aliper A. Putin E. (2019). Blood biochemistry analysis to detect smoking status and quantify accelerated aging in smokers. Sci. Rep. 9, 142. 10.1038/s41598-018-35704-w 30644411 PMC6333803

[B177] Marriott R. J. Murray K. Budgeon C. A. Codd V. Hui J. Arscott G. M. (2023). Serum testosterone and sex hormone-binding globulin are inversely associated with leucocyte telomere length in men: a cross-sectional analysis of the UK Biobank study. Eur. J. Endocrinol. 188. 236, 247. 10.1093/ejendo/lvad015 36751991

[B178] Martens D. S. Wei F. F. Cox B. Plusquin M. Thijs L. Winckelmans E. (2018). Retinal microcirculation and leukocyte telomere length in the general population. Sci. Rep. 8, 7095. 10.1038/s41598-018-25165-6 29728662 PMC5935741

[B179] Martínez de Toda I. Vida C. Díaz-Del Cerro E. De la Fuente M. (2021). The immunity clock. J. Gerontol. A Biol. Sci. Med. Sci. 76, 1939–1945. 10.1093/gerona/glab136 33979432

[B180] Martínez C. F. Esposito S. Di Castelnuovo A. Costanzo S. Ruggiero E. De Curtis A. (2023). Association between the inflammatory potential of the diet and biological aging: a cross-sectional analysis of 4510 adults from the moli-sani study cohort. Nutrients 15. 1503, 10.3390/nu15061503 36986232 PMC10056325

[B181] Mason S. M. Prescott J. Tworoger S. S. De Vivo I. Rich-Edwards J. W. (2015). Childhood physical and sexual abuse history and leukocyte telomere length among women in middle adulthood. PLoS One 10, e0124493. 10.1371/journal.pone.0124493 26053088 PMC4459951

[B182] Mastrobattista E. Lenze E. J. Reynolds C. F. Mulsant B. H. Wetherell J. Wu G. F. (2023). Late-life depression is associated with increased levels of GDF-15, a pro-aging mitokine. Am. J. Geriatr. Psychiatry 31, 1–9. 10.1016/j.jagp.2022.08.003 36153290 PMC9701166

[B183] Maté I. Martínez de Toda I. Arranz L. Álvarez-Sala J. L. De la Fuente M. (2021). Accelerated immunosenescence, oxidation and inflammation lead to a higher biological age in COPD patients. Exp. Gerontol. 154, 111551. 10.1016/j.exger.2021.111551 34530106

[B184] Maxwell F. McGlynn L. M. Muir H. C. Talwar D. Benzeval M. Robertson T. (2011). Telomere attrition and decreased fetuin-A levels indicate accelerated biological aging and are implicated in the pathogenesis of colorectal cancer. Clin. Cancer Res. 17, 5573–5581. 10.1158/1078-0432.CCR-10-3271 21753158

[B185] McClelland R. Christensen K. Mohammed S. McGuinness D. Cooney J. Bakshi A. (2016). Accelerated ageing and renal dysfunction links lower socioeconomic status and dietary phosphate intake. Aging (Albany NY) 8, 1135–1149. 10.18632/aging.100948 27132985 PMC4931858

[B186] McCrory C. Fiorito G. McLoughlin S. Polidoro S. Cheallaigh C. N. Bourke N. (2020). Epigenetic clocks and allostatic load reveal potential sex-specific drivers of biological aging. J. Gerontol. A Biol. Sci. Med. Sci. 75, 495–503. 10.1093/gerona/glz241 31603985

[B187] McFarland R. A. (1958). Health and safety in transportation. Public Health Rep. 73, 663–680. 13579104 PMC1951561

[B188] McLachlan K. J. J. Cole J. H. Harris S. E. Marioni R. E. Deary I. J. Gale C. R. (2020). Attitudes to ageing, biomarkers of ageing and mortality: the Lothian Birth Cohort 1936. J. Epidemiol. Community Health 74, 377–383. 10.1136/jech-2019-213462 31992610 PMC7079194

[B189] Mehta S. R. Iudicello J. E. Lin J. Ellis R. J. Morgan E. Okwuegbuna O. (2021). Telomere length is associated with HIV infection, methamphetamine use, inflammation, and comorbid disease risk. Drug Alcohol Depend. 221, 108639. 10.1016/j.drugalcdep.2021.108639 33621803 PMC8026664

[B190] Meier H. C. S. Parks C. G. Liu H. B. Sandler D. P. Simonsick E. M. Deane K. (2019). Cellular aging over 13 years associated with incident antinuclear antibody positivity in the Baltimore Longitudinal Study of Aging. J. Autoimmun. 105, 102295. 10.1016/j.jaut.2019.06.006 31303354 PMC6878149

[B191] Meisel P. Pink C. Nauck M. Völzke H. Kocher T. (2019). Construction of a biological age score to predict tooth loss over 10 years. J. Dent. Res. 98, 1096–1102. 10.1177/0022034519861037 31256728

[B192] Meng N. Zhou Y. Zhang Q. Yu X. Li H. Liu Y. (2023). Using inflammatory biological age to evaluate the preventing aging effect of a polyphenol-probiotic-enhanced dietary pattern in adults aged 50 years and older. J. Agric. Food Chem. 71, 6314–6325. 10.1021/acs.jafc.2c07241 37057839

[B193] Meyer A. Salewsky B. Spira D. Steinhagen-Thiessen E. Norman K. Demuth I. (2016). Leukocyte telomere length is related to appendicular lean mass: cross-sectional data from the Berlin aging study II (BASE-II). Am. J. Clin. Nutr. 103, 178–183. 10.3945/ajcn.115.116806 26675777

[B194] Minami R. Takahama S. Yamamoto M. (2019). Correlates of telomere length shortening in peripheral leukocytes of HIV-infected individuals and association with leukoaraiosis. PLoS One 14, e0218996. 10.1371/journal.pone.0218996 31246986 PMC6597162

[B195] Mitnitski A. Howlett S. E. Rockwood K. (2017). Heterogeneity of human aging and its assessment. J. Gerontol. A Biol. Sci. Med. Sci. 72, 877–884. 10.1093/gerona/glw089 27216811 PMC5458417

[B196] Movérare-Skrtic S. Johansson P. Mattsson N. Hansson O. Wallin A. Johansson J. O. (2012). Leukocyte telomere length (LTL) is reduced in stable mild cognitive impairment but low LTL is not associated with conversion to Alzheimer’s disease: a pilot study. Exp. Gerontol. 47, 179–182. 10.1016/j.exger.2011.12.005 22210159

[B197] Mukli P. Wu D. H. Csipo T. Owens C. D. Lipecz A. Racz F. S. (2022). Urinary biomarkers of oxidative stress in aging: implications for prediction of accelerated biological age in prospective cohort studies. Oxid. Med. Cell Longev. 2022, 6110226. 10.1155/2022/6110226 35571254 PMC9106456

[B198] Muniesa C. A. Verde Z. Diaz-Ureña G. Santiago C. Gutiérrez F. Díaz E. (2017). Telomere length in elite athletes. Int. J. Sports Physiol. Perform. 12, 994–996. 10.1123/ijspp.2016-0471 27918657

[B199] Muscari A. Bianchi G. Forti P. Magalotti D. Pandolfi P. Zoli M. (2021). N-terminal pro B-type natriuretic peptide (NT-proBNP): a possible surrogate of biological age in the elderly people. Geroscience 43, 845–857. 10.1007/s11357-020-00249-2 32780292 PMC8110633

[B200] Needham B. L. Salerno S. Roberts E. Boss J. Allgood K. L. Mukherjee B. (2019). Do black/white differences in telomere length depend on socioeconomic status? Biodemogr. Soc. Biol. 65, 287–312. 10.1080/19485565.2020.1765734 33243026 PMC7703670

[B201] Negasheva M. Lapshina N. Okushko R. Godina E. (2014). Biological age and tempos of aging in women over 60 in connection with their morphofunctional characteristics. J. Physiol. Anthropol. 33, 12. 10.1186/1880-6805-33-12 24887211 PMC4046622

[B202] Ng T. P. Zhong X. Gao Q. Gwee X. Chua D. Q. L. Larbi A. (2020). Socio-environmental, lifestyle, behavioural, and psychological determinants of biological ageing: the Singapore longitudinal ageing study. Gerontology 66, 603–613. 10.1159/000511211 33197920

[B203] Normando P. Bezerra F. F. Santana B. A. Calado R. T. Santos-Rebouças C. B. Epel E. S. (2020). Association between socioeconomic markers and adult telomere length differs according to sex: pro-Saúde study. Braz J. Med. Biol. Res. 53, e10223. 10.1590/1414-431X202010223 33053112 PMC7552895

[B204] Nusinovici S. Rim T. H. Yu M. Lee G. Tham Y. C. Cheung N. (2022). Retinal photograph-based deep learning predicts biological age, and stratifies morbidity and mortality risk. Age Ageing 51, afac065. 10.1093/ageing/afac065 PMC8973000 35363255

[B205] Nwanaji-Enwerem J. C. Colicino E. Dai L. Cayir A. Sanchez-Guerra M. Laue H. E. (2017). Impacts of the mitochondrial genome on the relationship of long-term ambient fine particle exposure with blood DNA methylation age. Environ. Sci. Technol. 51, 8185–8195. 10.1021/acs.est.7b02409 28636816 PMC5555236

[B206] Nwanaji-Enwerem J. C. Chung F. F. Van der Laan L. Novoloaca A. Cuenin C. Johansson H. (2021). An epigenetic aging analysis of randomized metformin and weight loss interventions in overweight postmenopausal breast cancer survivors. Clin. Epigenetics 13, 224. 10.1186/s13148-021-01218-y 34920739 PMC8684118

[B207] Ock J. Kim J. Choi Y. H. (2020). Organophosphate insecticide exposure and telomere length in U.S. adults. Sci. Total Environ. 709, 135990. 10.1016/j.scitotenv.2019.135990 31905589

[B208] Okazaki S. Kimura R. Otsuka I. Funabiki Y. Murai T. Hishimoto A. (2022). Epigenetic clock analysis and increased plasminogen activator inhibitor-1 in high-functioning autism spectrum disorder. PLoS One 17, e0263478. 10.1371/journal.pone.0263478 35113965 PMC8812940

[B209] Ormseth M. J. Solus J. F. Oeser A. M. Bian A. Gebretsadik T. Shintani A. (2016). Telomere length and coronary atherosclerosis in rheumatoid arthritis. J. Rheumatol. 43, 1469–1474. 10.3899/jrheum.151115 27252422 PMC4970932

[B210] Østhus I. B. Sgura A. Berardinelli F. Alsnes I. V. Brønstad E. Rehn T. (2012). Telomere length and long-term endurance exercise: does exercise training affect biological age? A pilot study. PLoS One 7, e52769. 10.1371/journal.pone.0052769 23300766 PMC3530492

[B211] O’Donovan A. Epel E. Lin J. Wolkowitz O. Cohen B. Maguen S. (2011a). Childhood trauma associated with short leukocyte telomere length in posttraumatic stress disorder. Biol. Psychiatry 70, 465–471. 10.1016/j.biopsych.2011.01.035 PMC3152637 21489410

[B212] O’Donovan A. Pantell M. S. Puterman E. Dhabhar F. S. Blackburn E. H. Yaffe K. (2011b). Cumulative inflammatory load is associated with short leukocyte telomere length in the health, aging and body composition study. PLoS One 6, e19687. 10.1371/journal.pone.0019687 21602933 PMC3094351

[B213] Pan R. Wang J. Chang W. W. Song J. Yi W. Zhao F. (2023). Association of PM(2.5) components with acceleration of aging: moderating role of sex hormones. Environ. Sci. Technol. 57, 3772–3782. 10.1021/acs.est.2c09005 36811885

[B214] Paparazzo E. Geracitano S. Lagani V. Bartolomeo D. Aceto M. A. D’Aquila P. (2022). A blood-based molecular clock for biological age estimation. Cells 12, 32. 10.3390/cells12010032 36611826 PMC9818068

[B215] Pathai S. Gilbert C. E. Lawn S. D. Weiss H. A. Peto T. Cook C. (2013a). Assessment of candidate ocular biomarkers of ageing in a South African adult population: relationship with chronological age and systemic biomarkers. Mech. Ageing Dev. 134, 338–345. 10.1016/j.mad.2013.05.002 23701820 PMC3710972

[B216] Pathai S. Lawn S. D. Gilbert C. E. McGuinness D. McGlynn L. Weiss H. A. (2013b). Accelerated biological ageing in HIV-infected individuals in South Africa: a case-control study. AIDS 27, 2375–2384. 10.1097/QAD.0b013e328363bf7f 23751258 PMC3805356

[B217] Pathai S. Shiels P. G. Weiss H. A. Gilbert C. E. Peto T. Bekker L. G. (2013c). Ocular parameters of biological ageing in HIV-infected individuals in South Africa: relationship with chronological age and systemic biomarkers of ageing. Mech. Ageing Dev. 134, 400–406. 10.1016/j.mad.2013.08.002 23994067 PMC3818088

[B218] Pavanello S. Campisi M. Tona F. Lin C. D. Iliceto S. (2019). Exploring epigenetic age in response to intensive relaxing training: a pilot study to slow Down biological age. Int. J. Environ. Res. Public Health 16, 3074. 10.3390/ijerph16173074 31450859 PMC6747190

[B219] Pearce E. E. Horvath S. Katta S. Dagnall C. Aubert G. Hicks B. D. (2021). DNA-methylation-based telomere length estimator: comparisons with measurements from flow FISH and qPCR. Aging (Albany NY) 13, 14675–14686. 10.18632/aging.203126 34083495 PMC8221337

[B220] Peters M. J. Joehanes R. Pilling L. C. Schurmann C. Conneely K. N. Powell J. (2015). The transcriptional landscape of age in human peripheral blood. Nat. Communications 6, 8570. 10.1038/ncomms9570 PMC4639797 26490707

[B221] Praveen G. Shalini T. Sivaprasad M. Reddy G. B. (2020). Relative telomere length and mitochondrial DNA copy number variation with age: association with plasma folate and vitamin B12. Mitochondrion 51, 79–87. 10.1016/j.mito.2020.01.007 31935457

[B222] Pruszkowska-Przybylska P. Brennecke S. Moses E. K. Melton P. E. (2022). Evaluation of epigenetic age calculators between preeclampsia and normotensive pregnancies in an Australian cohort. Sci. Rep. 12, 1664. 10.1038/s41598-022-05744-4 35102228 PMC8803933

[B223] Pyrkov T. V. Getmantsev E. Zhurov B. Avchaciov K. Pyatnitskiy M. Menshikov L. (2018a). Quantitative characterization of biological age and frailty based on locomotor activity records. Aging (Albany NY) 10, 2973–2990. 10.18632/aging.101603 30362959 PMC6224248

[B224] Pyrkov T. V. Slipensky K. Barg M. Kondrashin A. Zhurov B. Zenin A. (2018b). Extracting biological age from biomedical data *via* deep learning: too much of a good thing? Sci. Rep. 8, 5210. 10.1038/s41598-018-23534-9 29581467 PMC5980076

[B225] Raghu V. K. Weiss J. Hoffmann U. Aerts HJWL Lu M. T. (2021). Deep learning to estimate biological age from chest radiographs. JACC Cardiovasc Imaging 14, 2226–2236. 10.1016/j.jcmg.2021.01.008 33744131 PMC11381093

[B226] Rahman S. A. Adjeroh D. A. (2019). Deep learning using convolutional LSTM estimates biological age from physical activity. Sci. Rep. 9, 11425. 10.1038/s41598-019-46850-0 31388024 PMC6684608

[B227] Rahman S. A. Adjeroh D. A. (2020). Centroid of age neighborhoods: a new approach to estimate biological age. IEEE J. Biomed. Health Inf. 24, 1226–1234. 10.1109/JBHI.2019.2930938 31352357 PMC7362935

[B228] Rapp T. Ronchetti J. Sicsic J. (2022). Where are populations aging better? A global comparison of healthy aging across organization for economic cooperation and development countries. Value Health 25, 1520–1527. 10.1016/j.jval.2022.05.007 35710893

[B229] Raschenberger J. Kollerits B. Hammerer-Lercher A. Rantner B. Stadler M. Haun M. (2013). The association of relative telomere length with symptomatic peripheral arterial disease: results from the CAVASIC study. Atherosclerosis 229, 469–474. 10.1016/j.atherosclerosis.2013.05.027 23880207

[B230] Rasmussen L. J. H. Caspi A. Ambler A. Danese A. Elliott M. Eugen-Olsen J. (2021). Association between elevated suPAR, a new biomarker of inflammation, and accelerated aging. J. Gerontol. A Biol. Sci. Med. Sci. 76, 318–327. 10.1093/gerona/glaa178 32766674 PMC7812430

[B231] Rehkopf D. H. Dow W. H. Rosero-Bixby L. Lin J. Epel E. S. Blackburn E. H. (2013). Longer leukocyte telomere length in Costa Rica’s Nicoya Peninsula: a population-based study. Exp. Gerontol. 48, 1266–1273. 10.1016/j.exger.2013.08.005 23988653 PMC3819141

[B232] Rej P. H. Gravlee C. C. Mulligan C. J. (2020). Shortened telomere length is associated with unfair treatment attributed to race in African Americans living in Tallahassee, Florida. Am. J. Hum. Biol. 32, e23375. 10.1002/ajhb.23375 31867825

[B233] Rentscher K. E. Carroll J. E. Repetti R. L. Cole S. W. Reynolds B. M. Robles T. F. (2019). Chronic stress exposure and daily stress appraisals relate to biological aging marker p16(INK4a). Psychoneuroendocrinology 102, 139–148. 10.1016/j.psyneuen.2018.12.006 30557761 PMC6420375

[B234] Rentscher K. E. Carroll J. E. Cole S. W. Repetti R. L. Robles T. F. (2020). Relationship closeness buffers the effects of perceived stress on transcriptomic indicators of cellular stress and biological aging marker p16(INK4a). Aging (Albany NY) 12, 16476–16490. 10.18632/aging.103739 32712602 PMC7485710

[B235] Révész D. Verhoeven J. E. Milaneschi Y. de Geus E. J. Wolkowitz O. M. Penninx B. W. (2014). Dysregulated physiological stress systems and accelerated cellular aging. Neurobiol. Aging 35, 1422–1430. 10.1016/j.neurobiolaging.2013.12.027 24439483

[B236] Révész D. Verhoeven J. E. Picard M. Lin J. Sidney S. Epel E. S. (2017). Associations between cellular aging markers and metabolic syndrome: findings from the CARDIA study. J. Clin. Endocrinol. & Metabolism. 103, 148, 157. 10.1210/jc.2017-01625 29053810 PMC5761498

[B237] Roberts J. D. Dewland T. A. Longoria J. Fitzpatrick A. L. Ziv E. Hu D. (2014). Telomere length and the risk of atrial fibrillation: insights into the role of biological *versus* chronological aging. Circ. Arrhythm. Electrophysiol. 7, 1026–1032. 10.1161/CIRCEP.114.001781 25381796 PMC4294941

[B238] Robertson T. Batty G. D. Der G. Green M. J. McGlynn L. M. McIntyre A. (2012). Is telomere length socially patterned? Evidence from the West of Scotland Twenty-07 Study. PLoS One 7, e41805. 10.1371/journal.pone.0041805 22844525 PMC3402400

[B239] Robinson O. Chadeau Hyam M. Karaman I. Climaco Pinto R. Ala-Korpela M. Handakas E. (2020). Determinants of accelerated metabolomic and epigenetic aging in a UK cohort. Aging Cell 19, e13149. 10.1111/acel.13149 32363781 PMC7294785

[B240] Romanov G. A. Vanyushin B. F. (1981). Methylation of reiterated sequences in mammalian DNAs effects of the tissue type, age, malignancy and hormonal induction. Biochimica Biophysica Acta (BBA)-Nucleic Acids Protein Synthesis 653, 204–218. 10.1016/0005-2787(81)90156-8 7225396

[B241] Russo A. Palumbo L. Fornengo C. Di Gaetano C. Ricceri F. Guarrera S. (2012). Telomere length variation in juvenile acute myocardial infarction. PLoS One 7, e49206. 10.1371/journal.pone.0049206 23145125 PMC3492293

[B242] Ryan K. M. McLoughlin D. M. (2020). Telomere length in depression and association with therapeutic response to electroconvulsive therapy and cognitive side-effects. Psychol. Med. 50, 2096–2106. 10.1017/S0033291719002228 31477194

[B243] Safaee M. M. Lin J. Smith D. L. Fury M. Scheer J. K. Burke J. F. (2023). Association of telomere length with risk of complications in adult spinal deformity surgery: a pilot study of 43 patients. J. Neurosurg. Spine 38, 331–339. 10.3171/2022.10.SPINE22605 36461827

[B244] Salignon J. Rizzuto D. Calderón-Larrañaga A. Zucchelli A. Fratiglioni L. Riedel C. G. (2023). Beyond chronological age: a multidimensional approach to survival prediction in older adults. J. Gerontol. A Biol. Sci. Med. Sci. 78, 158–166. 10.1093/gerona/glac186 36075209 PMC9879753

[B245] Samuel L. J. Roth D. L. Schwartz B. S. Thorpe R. J. Glass T. A. (2018). Socioeconomic status, race/ethnicity, and diurnal cortisol trajectories in middle-aged and older adults. Journals Gerontology Ser. B Psychol. Sci. & Soc. Sci. 73, 468–476. 10.1093/geronb/gbw080 27440916 PMC5926996

[B246] Sanchis J. Ruiz V. Bonanad C. Sastre C. Ruescas A. Díaz M. (2019). Growth differentiation factor 15 and geriatric conditions in acute coronary syndrome. Int. J. Cardiol. 290, 15–20. 10.1016/j.ijcard.2019.05.034 31130280

[B247] Savolainen K. Eriksson J. G. Kananen L. Kajantie E. Pesonen A. K. Heinonen K. (2014). Associations between early life stress, self-reported traumatic experiences across the lifespan and leukocyte telomere length in elderly adults. Biol. Psychol. 97, 35–42. 10.1016/j.biopsycho.2014.02.002 24530884

[B248] Sawhney V. Campbell N. G. Brouilette S. W. Coppen S. R. Harbo M. Baker V. (2016). Telomere shortening and telomerase activity in ischaemic cardiomyopathy patients - potential markers of ventricular arrhythmia. Int. J. Cardiol. 207, 157–163. 10.1016/j.ijcard.2016.01.066 26803233

[B249] Schafer M. J. Zhang X. Kumar A. Atkinson E. J. Zhu Y. Jachim S. (2020). The senescence-associated secretome as an indicator of age and medical risk. JCI Insight 5, e133668. 10.1172/jci.insight.133668 32554926 PMC7406245

[B250] Schrempft S. Belsky D. W. Draganski B. Kliegel M. Vollenweider P. Marques-Vidal P. (2022). Associations between life-course socioeconomic conditions and the pace of aging. J. Gerontol. A Biol. Sci. Med. Sci. 77, 2257–2264. 10.1093/gerona/glab383 34951641

[B251] Schumann A. Gaser C. Sabeghi R. Schulze P. C. Festag S. Spreckelsen C. (2023). Using machine learning to estimate the calendar age based on autonomic cardiovascular function. Front. Aging Neurosci. 15, 1–10. 10.3389/fnagi.2022.899249 PMC9899796 36755773

[B252] Seki Y. Aczel D. Torma F. Jokai M. Boros A. Suzuki K. (2023). No strong association among epigenetic modifications by DNA methylation, telomere length, and physical fitness in biological aging. Biogerontology 24, 245–255. 10.1007/s10522-022-10011-0 36592269 PMC10006047

[B253] Senior A. M. Legault V. Lavoie F. B. Presse N. Gaudreau P. Turcot V. (2022). Multidimensional associations between nutrient intake and healthy ageing in humans. BMC Biol. 20, 196. 10.1186/s12915-022-01395-z 36050730 PMC9438070

[B254] Shalev I. Moffitt T. E. Braithwaite A. W. Danese A. Fleming N. I. Goldman-Mellor S. (2014). Internalizing disorders and leukocyte telomere erosion: a prospective study of depression, generalized anxiety disorder and post-traumatic stress disorder. Mol. Psychiatry 19, 1163–1170. 10.1038/mp.2013.183 24419039 PMC4098012

[B255] Shapiro I. Belsky D. W. Israel S. Youssim I. Friedlander Y. Hochner H. (2023). Familial aggregation of the aging process: biological age measured in young adult offspring as a predictor of parental mortality. Geroscience 45, 901–913. 10.1007/s11357-022-00687-0 36401109 PMC9886744

[B256] Shiels P. G. McGlynn L. M. MacIntyre A. Johnson P. C. Batty G. D. Burns H. (2011). Accelerated telomere attrition is associated with relative household income, diet and inflammation in the pSoBid cohort. PLoS One 6, e22521. 10.1371/journal.pone.0022521 21818333 PMC3144896

[B257] Shin C. Kim N. H. Baik I. (2016a). Sex-specific association between longitudinal changes in adiposity, FTO rs9939609 polymorphism, and leukocyte telomere length. J. Am. Coll. Nutr. 35, 245–254. 10.1080/07315724.2015.1005197 26260984

[B258] Shin C. Yun C. H. Yoon D. W. Baik I. (2016b). Association between snoring and leukocyte telomere length. Sleep 39, 767–772. 10.5665/sleep.5624 26715224 PMC4791610

[B259] Shin J. W. Lee E. Han S. Choe S. A. Jeon O. H. (2022). Plasma proteomic signature of cellular senescence and markers of biological aging among postmenopausal women. Rejuvenation Res. 25, 141–148. 10.1089/rej.2022.0024 35583231

[B260] Shirazi T. N. Hastings W. J. Rosinger A. Y. Ryan C. P. (2020). Parity predicts biological age acceleration in post-menopausal, but not pre-menopausal, women. Sci. Rep. 10, 20522. 10.1038/s41598-020-77082-2 33239686 PMC7689483

[B261] Shokr H. Lush V. Dias I. H. Ekárt A. De Moraes G. Gherghel D. (2022). The use of retinal microvascular function and telomere length in age and blood pressure prediction in individuals with low cardiovascular risk. Cells 11. 3037, 10.3390/cells11193037 36230999 PMC9563868

[B262] Simons R. L. Lei M.-K. Beach S. R. H. Simons L. G. Barr A. B. Gibbons F. X. (2019). Testing life course models whereby juvenile and adult adversity combine to influence speed of biological aging. J. Health Soc. Behav. 60, 291–308. 10.1177/0022146519859896 31409156 PMC7751897

[B263] Smeets C. C. Codd V. Samani N. J. Hokken-Koelega A. C. (2015). Leukocyte telomere length in young adults born preterm: support for accelerated biological ageing. PLoS One 10, e0143951. 10.1371/journal.pone.0143951 26619005 PMC4664383

[B264] Sol J. Obis È. Mota-Martorell N. Pradas I. Galo-Licona J. D. Martin-Garí M. (2023). Plasma acylcarnitines and gut-derived aromatic amino acids as sex-specific hub metabolites of the human aging metabolome. Aging Cell 22, e13821. 10.1111/acel.13821 36951231 PMC10265170

[B265] Sood S. Gallagher I. J. Lunnon K. Rullman E. Keohane A. Crossland H. (2015). A novel multi-tissue RNA diagnostic of healthy ageing relates to cognitive health status. Genome Biol. 16, 185. 10.1186/s13059-015-0750-x 26343147 PMC4561473

[B266] Srour B. Hynes L. C. Johnson T. Kühn T. Katzke V. A. Kaaks R. (2022). Serum markers of biological ageing provide long-term prediction of life expectancy-a longitudinal analysis in middle-aged and older German adults. Age Ageing 51, afab271. 10.1093/ageing/afab271 35150586

[B267] St Sauver J. Rocca W. LeBrasseur N. Chamberlain A. Olson J. Jacobson D. (2022). Inflammatory biomarkers, multi-morbidity, and biologic aging. J. Int. Med. Res. 50, 3000605221109393. 10.1177/03000605221109393 35796512 PMC9274410

[B268] Sternäng O. Palmer K. Kabir Z. N. Hasan M. I. Wahlin Å. (2019). Associations between functional biological age and cognition among older adults in rural Bangladesh: comparisons with chronological age. J. Aging Health 31, 814–836. 10.1177/0898264318757147 29441812

[B269] Strandberg T. E. Saijonmaa O. Tilvis R. S. Pitkälä K. H. Strandberg A. Y. Miettinen T. A. (2011). Association of telomere length in older men with mortality and midlife body mass index and smoking. J. Gerontol. A Biol. Sci. Med. Sci. 66, 815–820. 10.1093/gerona/glr064 21697500

[B270] Strandberg T. E. Strandberg A. Y. Saijonmaa O. Tilvis R. S. Pitkälä K. H. Fyhrquist F. (2012). Association between alcohol consumption in healthy midlife and telomere length in older men. The Helsinki Businessmen Study. Eur. J. Epidemiol. 27, 815–822. 10.1007/s10654-012-9728-0 22875407

[B271] Strazhesko I. Tkacheva O. Kashtanova D. Ivanov M. Kljashtorny V. Esakova A. (2022). Physiological health indexes predict deterioration and mortality in patients with COVID-19: a comparative study. Aging (Albany NY) 14, 1611–1626. 10.18632/aging.203915 35213841 PMC8908924

[B272] Sumida K. Han Z. Dashputre A. A. Potukuchi P. K. Kovesdy C. P. (2020). Association between Nrf2 and CDKN2A expression in patients with end-stage renal disease: a pilot study. Aging (Albany NY) 12, 16357–16367. 10.18632/aging.103685 32661200 PMC7485736

[B273] Surtees P. G. Wainwright N. W. Pooley K. A. Luben R. N. Khaw K. T. Easton D. F. (2012). Educational attainment and mean leukocyte telomere length in women in the European Prospective Investigation into Cancer (EPIC)-Norfolk population study. Brain Behav. Immun. 26, 414–418. 10.1016/j.bbi.2011.11.009 22178899

[B274] Suvarna H. I. S. Moodithaya S. Sharma R. (2017). Metabolic and cardiovascular ageing indices in relation to glycated haemoglobin in healthy and diabetic subjects. Curr. Aging Sci. 10, 201–210. 10.2174/1874609810666170216124039 28215180

[B275] Tian R. Zhang L. N. Zhang T. T. Pang H. Y. Chen L. F. Shen Z. J. (2017). Association between oxidative stress and peripheral leukocyte telomere length in patients with premature coronary artery disease. Med. Sci. Monit. 23, 4382–4390. 10.12659/msm.902106 28892468 PMC5604488

[B276] Topiwala A. Taschler B. Ebmeier K. P. Smith S. Zhou H. Levey D. F. (2022). Alcohol consumption and telomere length: mendelian randomization clarifies alcohol’s effects. Mol. Psychiatry 27, 4001–4008. 10.1038/s41380-022-01690-9 35879401 PMC9718662

[B277] Toyoshima K. Seino S. Tamura Y. Ishikawa J. Chiba Y. Ishizaki T. (2022). Difference between “Physical Fitness Age” based on physical function and chronological age is associated with obesity, hyperglycemia, depressive symptoms, and low serum albumin. J. Nutr. Health Aging 26, 501–509. 10.1007/s12603-022-1786-8 35587763 PMC12879148

[B278] Tricco A. C. Lillie E. Zarin W. O’Brien K. K. Colquhoun H. Levac D. (2018). PRISMA extension for scoping reviews (PRISMA-ScR): checklist and explanation. Ann. Intern. Med. 169, 467–473. 10.7326/M18-0850 30178033

[B279] Tucker L. A. (2017). Alpha- and gamma-tocopherol and telomere length in 5768 US men and women: a NHANES study. Nutrients 9, 601. 10.3390/nu9060601 28629117 PMC5490580

[B280] Tucker L. A. (2018). Dietary fiber and telomere length in 5674 U.S. adults: an NHANES study of biological aging. Nutrients 10, 400. 10.3390/nu10040400 29570620 PMC5946185

[B281] Tucker L. A. (2019). Milk fat intake and telomere length in U.S. women and men: the role of the milk fat fraction. Oxid. Med. Cell Longev. 2019, 1574021. 10.1155/2019/1574021 31772698 PMC6855010

[B282] Tucker L. A. (2022). Insulin resistance and biological aging: the role of body mass, waist circumference, and inflammation. Biomed. Res. Int. 2022, 2146596. 10.1155/2022/2146596 35586815 PMC9110194

[B283] Tucker L. A. Giacomello E. Toniolo L. (2021). Fruit and vegetable intake and telomere length in a random sample of 5448 U.S. adults. Nutrients 13, 1415. 10.3390/nu13051415 33922436 PMC8146059

[B284] Verhoeven J. E. Révész D. Epel E. S. Lin J. Wolkowitz O. M. Penninx B. W. (2014). Major depressive disorder and accelerated cellular aging: results from a large psychiatric cohort study. Mol. Psychiatry 19, 895–901. 10.1038/mp.2013.151 24217256

[B285] Verschoor C. P. Belsky D. W. Ma J. Cohen A. A. Griffith L. E. Raina P. (2021). Comparing biological age estimates using domain-specific measures from the Canadian longitudinal study on aging. J. Gerontol. A Biol. Sci. Med. Sci. 76, 187–194. 10.1093/gerona/glaa151 32598446 PMC7812432

[B286] Vetter V. M. Meyer A. Karbasiyan M. Steinhagen-Thiessen E. Hopfenmüller W. Demuth I. (2019). Epigenetic clock and relative telomere length represent largely different aspects of aging in the Berlin aging study II (BASE-II). J. Gerontol. A Biol. Sci. Med. Sci. 74, 27–32. 10.1093/gerona/gly184 30137208

[B287] Vetter V. M. Kalies C. H. Sommerer Y. Spira D. Drewelies J. Regitz-Zagrosek V. (2022). Relationship between 5 epigenetic clocks, telomere length, and functional capacity assessed in older adults: cross-sectional and longitudinal analyses. J. Gerontol. A Biol. Sci. Med. Sci. 77, 1724–1733. 10.1093/gerona/glab381 35032170

[B288] Vincent J. Hovatta I. Frissa S. Goodwin L. Hotopf M. Hatch S. L. (2017). Assessing the contributions of childhood maltreatment subtypes and depression case-control status on telomere length reveals a specific role of physical neglect. J. Affect Disord. 213, 16–22. 10.1016/j.jad.2017.01.031 28187293 PMC6191534

[B289] von Falkenhausen A. S. Freudling R. Waldenberger M. Gieger C. Peters A. Müller-Nurasyid M. (2022). Common electrocardiogram measures are not associated with telomere length. Aging (Albany NY) 14, 5620–5627. 10.18632/aging.204149 35787517 PMC9365565

[B290] von Känel R. Malan N. T. Hamer M. van der Westhuizen F. H. Malan L. (2014). Leukocyte telomere length and hemostatic factors in a South African cohort: the SABPA study. J. Thromb. Haemost. 12, 1975–1985. 10.1111/jth.12733 25244563

[B291] von Känel R. Malan N. T. Hamer M. Malan L. (2015). Comparison of telomere length in black and white teachers from South Africa: the sympathetic activity and ambulatory blood pressure in Africans study. Psychosom. Med. 77, 26–32. 10.1097/PSY.0000000000000123 25469684

[B292] Vyas C. M. Hazra A. Chang S. C. Qiu W. Reynolds C. F. 3rd Mischoulon D. (2019). Pilot study of DNA methylation, molecular aging markers and measures of health and well-being in aging. Transl. Psychiatry 9, 118. 10.1038/s41398-019-0446-1 30886137 PMC6423054

[B293] Vyas C. M. Ogata S. Reynolds C. F. 3rd Mischoulon D. Chang G. Cook N. R. (2020). Lifestyle and behavioral factors and mitochondrial DNA copy number in a diverse cohort of mid-life and older adults. PLoS One 15, e0237235. 10.1371/journal.pone.0237235 32785256 PMC7423118

[B294] Waaijer M. E. Gunn D. A. Catt S. D. van Ginkel M. de Craen A. J. Hudson N. M. (2012). Morphometric skin characteristics dependent on chronological and biological age: the leiden longevity study. Age (Dordr) 34, 1543–1552. 10.1007/s11357-011-9314-5 21909657 PMC3528376

[B295] Waaijer M. E. Wieser M. Grillari-Voglauer R. van Heemst D. Grillari J. Maier A. B. (2014). MicroRNA-663 induction upon oxidative stress in cultured human fibroblasts depends on the chronological age of the donor. Biogerontology 15, 269–278. 10.1007/s10522-014-9496-1 24664125

[B296] Waaijer M. E. Croco E. Westendorp R. G. Slagboom P. E. Sedivy J. M. Lorenzini A. (2016). Human aging, 53bp1, telomere-associated foci, micronuclei, biological age. Aging (Albany NY) 8, 147–157. 10.18632/aging.100890 26830451 PMC4761719

[B297] Wagenmakers M. J. Oudega M. L. Klaus F. Wing D. Orav G. Han L. K. M. (2023). BrainAge of patients with severe late-life depression referred for electroconvulsive therapy. J. Affect Disord. 330, 1–6. 10.1016/j.jad.2023.02.047 36858270

[B298] Wan E. S. Goldstein R. L. Garshick E. DeMeo D. L. Moy M. L. (2021). Molecular markers of aging, exercise capacity, & physical activity in COPD. Respir. Med. 187, 106576. 10.1016/j.rmed.2021.106576 34416615

[B299] Wang C. Guan X. Bai Y. Feng Y. Wei W. Li H. (2022). A machine learning-based biological aging prediction and its associations with healthy lifestyles: the Dongfeng-Tongji cohort. Ann. N. Y. Acad. Sci. 1507, 108–120. 10.1111/nyas.14685 34480349

[B300] Ward-Caviness C. K. Nwanaji-Enwerem J. C. Wolf K. Wahl S. Colicino E. Trevisi L. (2016). Long-term exposure to air pollution is associated with biological aging. Oncotarget 7, 74510–74525. 10.18632/oncotarget.12903 27793020 PMC5342683

[B301] Watkins L. E. Harpaz-Rotem I. Sippel L. M. Krystal J. H. Southwick S. M. Pietrzak R. H. (2016). Hostility and telomere shortening among U.S. military veterans: results from the National Health and Resilience in Veterans study. Psychoneuroendocrinology 74, 251–257. 10.1016/j.psyneuen.2016.09.006 27689898

[B302] Waziry R. Gras L. Sedaghat S. Tiemeier H. Weverling G. J. Ghanbari M. (2019). Quantification of biological age as a determinant of age-related diseases in the Rotterdam study: a structural equation modeling approach. Eur. J. Epidemiol. 34, 793–799. 10.1007/s10654-019-00497-3 30993509

[B303] Weber J. Jörres R. Kronseder A. Müller A. Weigl M. Chmelar C. (2019). Learning on the job, the use of selection, optimization, and compensation strategies, and their association with telomere length as an indicator of biological aging. Int. Arch. Occup. Environ. Health 92, 361–370. 10.1007/s00420-019-01408-5 30671630

[B304] Welle S. Bhatt K. Shah B. Needler N. Delehanty J. M. Thornton C. A. (2003). Reduced amount of mitochondrial DNA in aged human muscle. J. Appl. Physiology 94, 1479–1484. 10.1152/japplphysiol.01061.2002 12496142

[B305] Wikgren M. Karlsson T. Söderlund H. Nordin A. Roos G. Nilsson L. G. (2014). Shorter telomere length is linked to brain atrophy and white matter hyperintensities. Age Ageing 43, 212–217. 10.1093/ageing/aft172 24231584

[B306] Wilson G. A. Cheyne K. Ramrakha S. Ambler A. Tan G. S. Caspi A. (2023). Are macular drusen in midlife a marker of accelerated biological ageing? Clin. Exp. Optom. 106, 41–46. 10.1080/08164622.2021.2012428 34902293

[B307] Wium-Andersen M. K. Ørsted D. D. Rode L. Bojesen S. E. Nordestgaard B. G. (2017). Telomere length and depression: prospective cohort study and Mendelian randomisation study in 67 306 individuals. Br. J. Psychiatry 210, 31–38. 10.1192/bjp.bp.115.178798 27810892

[B308] Wiweko B. Prawesti D. M. Hestiantoro A. Sumapraja K. Natadisastra M. Baziad A. (2013). Chronological age vs biological age: an age-related normogram for antral follicle count, FSH and Anti-Mullerian hormone. J. Assist. Reprod. Genet. 30, 1563–1567. 10.1007/s10815-013-0083-1 23955628 PMC3843177

[B309] Wolkowitz O. M. Jeste D. V. Martin A. S. Lin J. Daly R. E. Reuter C. (2017). Leukocyte telomere length: effects of schizophrenia, age, and gender. J. Psychiatr. Res. 85, 42–48. 10.1016/j.jpsychires.2016.10.015 27835738

[B310] Wynchank D. Bijlenga D. Penninx B. W. Lamers F. Beekman A. T. Kooij J. J. S. (2019). Delayed sleep-onset and biological age: late sleep-onset is associated with shorter telomere length. Sleep 42, zsz139. 10.1093/sleep/zsz139 31270544

[B311] Xia X. Chen X. Wu G. Li F. Wang Y. Chen Y. (2020). Three-dimensional facial-image analysis to predict heterogeneity of the human ageing rate and the impact of lifestyle. Nat. Metab. 2, 946–957. 10.1038/s42255-020-00270-x 32895578

[B312] Xie R. Ning Z. Xiao M. Li L. Liu M. Zhang Y. (2023). Dietary inflammatory potential and biological aging among US adults: a population-based study. Aging Clin. & Exp. Res. 35, 1273–1281. 10.1007/s40520-023-02410-1 37186209

[B313] Yang Q. Gao S. Lin J. Lyu K. Wu Z. Chen Y. (2022). A machine learning-based data mining in medical examination data: a biological features-based biological age prediction model. BMC Bioinforma. 23, 411. 10.1186/s12859-022-04966-7 36192681 PMC9528174

[B314] Yeap B. B. Knuiman M. W. Divitini M. L. Hui J. Arscott G. M. Handelsman D. J. (2016). Epidemiological and mendelian randomization studies of dihydrotestosterone and Estradiol and leukocyte telomere length in men. J. Clin. Endocrinol. Metab. 101, 1299–1306. 10.1210/jc.2015-4139 26789780

[B315] Yeap B. B. Hui J. Knuiman M. W. Handelsman D. J. Flicker L. Divitini M. L. (2019). Cross-sectional associations of sex hormones with leucocyte telomere length, a marker of biological age, in a community-based cohort of older men. Clin. Endocrinol. (Oxf) 90, 562–569. 10.1111/cen.13918 30561819

[B316] Yeap B. B. Hui J. Knuiman M. W. S A Paul C. Ken K. Y. H. Flicker L. (2020). Associations of plasma IGF1, IGFBP3 and estradiol with leucocyte telomere length, a marker of biological age, in men. Eur. J. Endocrinol. 182, 23–33. 10.1530/EJE-19-0638 31658437

[B317] Yen Y. C. Lung F. W. (2013). Older adults with higher income or marriage have longer telomeres. Age Ageing 42, 234–239. 10.1093/ageing/afs122 22951603 PMC3575119

[B318] Yu R. Tang N. Leung J. Woo J. (2015). Telomere length is not associated with frailty in older Chinese elderly: cross-sectional and longitudinal analysis. Mech. Ageing Dev. 152, 74–79. 10.1016/j.mad.2015.10.002 26483096

[B319] Yu X. Wang Y. Kristic J. Dong J. Chu X. Ge S. (2016). Profiling IgG N-glycans as potential biomarker of chronological and biological ages: a community-based study in a Han Chinese population. Med. Baltim. 95, e4112. 10.1097/MD.0000000000004112 27428197 PMC4956791

[B320] Yu J. Mathi Kanchi M. Rawtaer I. Feng L. Kumar A. P. Kua E.-H. (2022). Differences between multimodal brain-age and chronological-age are linked to telomere shortening. Neurobiol. Aging 115, 60–69. 10.1016/j.neurobiolaging.2022.03.015 35472831 PMC9133148

[B321] Żelaźniewicz A. Nowak-Kornicka J. Osochocka A. Pawłowski B. (2022). Perceived facial age and biochemical indicators of glycemia in adult men and women. Sci. Rep. 12, 10149. 10.1038/s41598-022-14555-6 35710822 PMC9203806

[B322] Zhang W. G. Bai X. J. Sun X. F. Cai G. Y. Bai X. Y. Zhu S. Y. (2014a). Construction of an integral formula of biological age for a healthy Chinese population using principle component analysis. J. Nutr. Health Aging 18, 137–142. 10.1007/s12603-013-0345-8 24522464 PMC12879004

[B323] Zhang W. G. Zhu S. Y. Bai X. J. Zhao D. L. Jian S. M. Li J. (2014b). Select aging biomarkers based on telomere length and chronological age to build a biological age equation. Age (Dordr) 36, 9639. 10.1007/s11357-014-9639-y 24659482 PMC4082565

[B324] Zhang W. G. Zhu S. Y. Zhao D. L. Jiang S. M. Li J. Li Z. X. (2014c). The correlation between peripheral leukocyte telomere length and indicators of cardiovascular aging. Heart Lung Circ. 23, 883–890. 10.1016/j.hlc.2013.12.016 24881030

[B325] Zhang W. Jia L. Cai G. Shao F. Lin H. Liu Z. (2017). Model construction for biological age based on a cross-sectional study of a healthy Chinese Han population. J. Nutr. Health Aging 21, 1233–1239. 10.1007/s12603-017-0874-7 29188884 PMC12879860

[B326] Zhang X. Wolff M. S. Shen J. Parada H. Jr Santella R. M. Neugut A. I. (2022). Phthalates and phenols, leukocyte telomere length, and breast cancer risk and mortality in the Long Island breast cancer study project. Cancer Epidemiol. Biomarkers Prev. 31, 117–123. 10.1158/1055-9965.EPI-21-0830 34697054 PMC8755624

[B327] Zhao Q. Zhu Y. Yeh F. Lin J. Lee E. T. Cole S. A. (2016). Depressive symptoms are associated with leukocyte telomere length in American Indians: findings from the strong heart family study. Aging (Albany NY) 8, 2961–2970. 10.18632/aging.101104 27870638 PMC5191880

[B328] Zhao X X. van Wijk E. Yan Y. van Wijk R. Yang H. Zhang Y. (2016). Ultra-weak photon emission of hands in aging prediction. J. Photochem Photobiol. B 162, 529–534. 10.1016/j.jphotobiol.2016.07.030 27472904

[B329] Zhao H. Shen J. Chang D. Ye Y. Wu X. Chow W. H. (2021). Land use mix and leukocyte telomere length in Mexican Americans. Sci. Rep. 11, 19742. 10.1038/s41598-021-99171-6 34611226 PMC8492751

[B330] Zhong X. Lu Y. Gao Q. Nyunt M. S. Z. Fulop T. Monterola C. P. (2020). Estimating biological age in the Singapore longitudinal aging study. J. Gerontol. A Biol. Sci. Med. Sci. 75, 1913–1920. 10.1093/gerona/glz146 31179487

[B331] Zhu H. Chen J. Liu K. Gao L. Wu H. Ma L. (2023). Human PBMC scRNA-seq-based aging clocks reveal ribosome to inflammation balance as a single-cell aging hallmark and super longevity. Sci. Adv. 9, eabq7599. 10.1126/sciadv.abq7599 37379396 PMC10306289

[B332] Ziesel A. Reeves J. Mallidou A. Newton L. Rhodes R. Zhang J. (2024). Markers, mechanisms and metrics of biological aging: a scoping review. bioRxiv, 10. 10.1101/2024.10.29.620898

[B333] Ziesel A. Reeves J. Mallidou A. Newton L. Rhodes R. E. Zhang J. (2025). Methylation and algorithms in biological aging: a scoping review. Front. Aging 6, 1682873. 10.3389/fragi.2025.1682873 41488274 PMC12756485

