# Supplementary material for: Molecular markers, mechanisms and metrics of biological aging: a scoping review
Source: Front Aging. 2026 Sep 8;7:1682867. doi: 10.3389/fragi.2026.1682867 (PMC13595061; doi:10.3389/fragi.2026.1682867)
Supplement: Supplementary file 1 [file DataSheet1.pdf]

Supplementary Material

1 APPENDIX A. SEARCH STRATEGIES, INCLUSION AND EXCLUSION CRITERIA

1.1 Search Strategies

The following search terms were employed for publication database searches. Note that 'present' in search filters indicates June 2023.

1.1.1 PubMed

Search number	Query terms
1	"Aging" [Title/Abstract] OR "Epigenetics" [Title/Abstract] OR "Telomeres" [Title/Abstract]
2	"biological age" [Title/Abstract] OR "biological ageing" [Title/Abstract] OR "biological aging" [Title/Abstract]
3	telomer* [Title Abstract] OR methyl* [Title/Abstract] OR DNAm[Title/Abstract] OR "epigenetic age" [Title/Abstract] OR "epigenetic ageing "[Title/Abstract] OR "epigenetic aging" [Title Abstract] OR "age estimator" [Title/Abstract] OR "epigenetic clock" [Title/Abstract] OR "biological clock" [Title/Abstract] OR "methylation clock" [Title/Abstract] OR "age predictor" [Title/Abstract] OR "age estimation" [Title/Abstract] OR "age acceleration" [Title/Abstract] OR "accelerated age" [Title/Abstract] OR "estimate age" [Title/Abstract] OR "Dnam Age" [Title/Abstract] OR "methylation age" [Title/Abstract] OR methylation profile [Title/Abstract] OR "aging rate" [Title/Abstract] OR "ageing rate" [Title/Abstract] OR "biological markers "[Title/Abstract] OR biomarkers [Title/Abstract] OR "chronological age" [Title/Abstract] OR "chronological aging" [Title/Abstract] OR "chronological ageing" [Title/Abstract]
4	(((((DNA Methylation [MeSH Terms]) OR (Epigenesis, Genetic [MeSH Terms])) OR (Telomere Shortening [MeSH Terms])) OR (Biological Clocks/Physiology [MeSH Terms])) OR (Biological Clocks/Genetics [MeSH Terms])) OR (Aging/Genetics [MeSH Terms])
5	#1 OR #3
6	#2 AND #4 Filters: from 2011 –
7	#2 AND #4 Filters: Humans, from 2011 -
8	#2 AND #4 Filters: Humans, Adult: 19+ years, from 2011
9	#2 AND #4 Filters: Humans, Adult: 19+ years, English, from 2011 -

1.1.2 CINAHL Abstracts

Search number	Query terms	Limiters
1	AB ”Aging” OR ”Epigenetics” ”Telomeres”	Expanders - Apply equivalent subjects Search modes -Boolean/Phrase
2	AB ”biological age” OR ”biological ageing” OR ”biological aging”	Search modes - Boolean/Phrase
3	AB telomer* OR methyl* OR DNAm OR ”epigenetic age” OR ”epigenetic ageing” OR ”epigenetic aging” OR ”age estimator” OR ”epigenetic clock” OR ”biological clock” OR ”methylation clock” OR ”age predictor” OR ”age estimation” OR ”age acceleration” OR “predict age” OR ”accelerated age” OR “estimate age” OR “Dnam Age” OR “methylation age” OR ”methylation profile” OR ”model of aging” OR ”model of ageing” OR ”aging rate” OR ”ageing rate” OR ”biological markers” OR biomarkers OR ”chronological age” OR”chronological aging” OR ”chronological ageing”	Search modes - Boolean/Phrase
4	S1 OR S3	Search modes - Boolean/Phrase
5	S2 AND S4	Search modes - Boolean/Phrase
6	S2 AND S4	Limiters - Published Date: 20110101-; English Language; Human; Age Groups: All Adult

1.1.3 CINAHL Titles

Search number	Query terms	Limiters
1	TI ”Aging” OR ”Epigenetics” OR ”Telomeres”	Expanders - Apply equivalent subjects Search modes - Boolean/Phrase
2	TI ”biological age” OR ”biological ageing” OR ”biological aging”	Search modes - Boolean/Phrase
3	TI telomer* OR methyl* OR DNAm OR ”epigenetic age” OR ”epigenetic ageing” OR ”epigenetic aging” OR ”age estimator” OR ”epigenetic clock” OR ”biological clock” OR ”methylation clock” OR ”age predictor” OR ”age estimation” OR ”age acceleration” OR “predict age” OR ”accelerated age” OR “estimate age” OR “Dnam Age” OR “methylation age” OR ”methylation profile” OR ”model of aging” OR ”model of ageing” OR ”aging rate” OR ”ageing rate” OR ”biological markers” OR biomarkers OR ”chronological age” OR ”chronological aging” OR ”chronological ageing”	Search modes - Boolean/Phrase
4	S1 OR S3	Search modes - Boolean/Phrase
5	S2 AND S4	Search modes - Boolean/Phrase
6	S2 AND S4	Limiters - Published Date: 20110101- ; English Language; Human; Age Groups: All Adult

1.1.4 PsycINFO Abstracts

Search number	Query terms	Limiters
1	MA ”Aging” OR MA ”Epigenetics” OR MA ”Telomeres”	Search modes - Boolean/Phrase
2	AB ”biological age” OR ”biological ageing” OR ”biological aging”	Search modes - Boolean/Phrase
3	AB telomer* OR methyl* OR DNAm OR ”epigenetic age” OR ”epigenetic ageing” OR ”epigenetic aging” OR ”age estimator” OR ”epigenetic clock” OR ”biological clock” OR ”methylation clock” OR ”age predictor” OR ”age estimation” OR ”age acceleration” OR “predict age” OR ”accelerated age” OR “estimate age” OR “Dnam Age” OR “methylation age” OR ”methylation profile” OR ”model of aging” OR ”model of ageing” OR ”aging rate” OR ”ageing rate” OR ”biological markers” OR biomarkers OR ”chronological age” OR ”chronological aging” OR ”chronological ageing”	Search modes - Boolean/Phrase
4	S1 OR S3	Search modes - Boolean/Phrase
5	S2 AND S4	Search modes - Boolean/Phrase
6	S2 AND S4	Limiters - Publication Year: 2011-; Age Groups: Adulthood (18 yrs & older); Population Group: Human

1.1.5 PsycINFO Titles

Search number	Query terms	Limiters
1	MA ”Aging” OR MA ”Epigenetics” OR MA ”Telomeres”	Search modes - Boolean/Phrase
2	TI ”biological age” OR ”biological ageing” OR ”biological aging”	Search modes - Boolean/Phrase
3	TI telomer* OR methyl* OR DNAm OR ”epigenetic age” OR ”epigenetic ageing” OR ”epigenetic aging” OR ”age estimator” OR ”epigenetic clock” OR ”biological clock” OR ”methylation clock” OR ”age predictor” OR ”age estimation” OR ”age acceleration” OR “predict age” OR ”accelerated age” OR “estimate age” OR “Dnam Age” OR “methylation age” OR ”methylation profile” OR ”model of aging” OR ”model of ageing” OR ”aging rate” OR ”ageing rate” OR ”biological markers” OR biomarkers OR ”chronological age” OR ”chronological aging” OR ”chronological ageing”	Search modes - Boolean/Phrase
4	S1 OR S3	Search modes - Boolean/Phrase
5	S2 AND S4	Search modes - Boolean/Phrase
6	S2 AND S4	Limiters - Publication Year: 2011-; Age Groups: Adulthood (18 yrs & older); Population Group: Human

1.1.6 SPORTDiscus Titles

Search number	Query terms	Limiters
1	AB ”Aging” OR MA ”Epigenetics” OR MA ”Telomeres”	Search modes - Boolean/Phrase
2	AB ”biological age” OR ”biological ageing” OR ”biological aging”	Search modes - Boolean/Phrase
3	AB telomer* OR methyl* OR DNAm OR ”epigenetic age” OR ”epigenetic ageing” OR ”epigenetic aging” OR ”age estimator” OR ”epigenetic clock” OR ”biological clock” OR ”methylation clock” OR ”age predictor” OR ”age estimation” OR ”age acceleration” OR “predict age” OR ”accelerated age” OR “estimate age” OR “Dnam Age” OR “methylation age” OR ”methylation profile” OR ”model of aging” OR ”model of ageing” OR ”aging rate” OR ”ageing rate” OR ”biological markers” OR biomarkers OR ”chronological age” OR ”chronological aging” OR ”chronological ageing”	Search modes - Boolean/Phrase
4	S1 OR S3	Search modes - Boolean/Phrase
5	S2 AND S4	Search modes - Boolean/Phrase
6	S2 AND S4	Limiters - Published Date: 20110101-; Language: English

1.1.7 SPORTDiscus Abstracts

Search number	Query terms	Limiters
1	AB ”Aging” OR MA ”Epigenetics” OR MA ”Telomeres”	Search modes - Boolean/Phrase
2	AB ”biological age” OR ”biological ageing” OR ”biological aging”	Search modes - Boolean/Phrase
3	AB telomer* OR methyl* OR DNAm OR ”epigenetic age” OR ”epigenetic ageing” OR ”epigenetic aging” OR ”age estimator” OR ”epigenetic clock” OR ”biological clock” OR ”methylation clock” OR ”age predictor” OR ”age estimation” OR ”age acceleration” OR “predict age” OR ”accelerated age” OR “estimate age” OR “Dnam Age” OR “methylation age” OR ”methylation profile” OR ”model of aging” OR ”model of ageing” OR ”aging rate” OR ”ageing rate” OR ”biological markers” OR biomarkers OR ”chronological age” OR ”chronological aging” OR ”chronological ageing”	Search modes - Boolean/Phrase
4	S1 OR S3	Search modes - Boolean/Phrase
5	S2 AND S4	Search modes - Boolean/Phrase
6	S2 AND S4	Limiters - Published Date: 20110101-; Language: English

1.2 Inclusion and Exclusion Criteria

Criteria	Eligibility
Population	**Include**: humans aged 18 and older<br>**Exclude**: focuses on humans aged under 18; animal studies
Outcome	**Include**: includes chronological age; compares either a calculated biological age or a predictor of biological aging with chronological age<br>**Exclude**: does not compare either a calculated biological age or a predictor of biological aging with chronological age
Setting	**Include**: any country
Study Design	**Include**: empirical studies, peer reviewed and published in a journal<br>**Exclude**: non-research (including opinion pieces, commentaries, letters, editorials); grey literature (including theses and dissertations); theoretical and seminar papers; case studies; book chapters; study protocols; review articles; articles in press
Language	**Include**: English language<br>**Exclude**: Non-English language
Date Range	**Include**: any included studies published between January 2011 and June 2023<br>**Exclude**: published prior to 2011 or after June 2023
