# Supplementary material for: Molecular markers, mechanisms and metrics of biological aging: a scoping review
Source: Front Aging. 2026 Sep 8;7:1682867. doi: 10.3389/fragi.2026.1682867 (PMC13595061; doi:10.3389/fragi.2026.1682867)
Supplement: Supplementary file 2 [file DataSheet2.pdf]

Primary Author	Title	Publication Year	Keywords
Accardi	miR-126-3p and miR-21-5p as Hallmarks of Bio-Positive Ageing; Correlation Analysis and Machine Learning Prediction in Young to Ultra-Centenarian Sicilian Population	2022	ageing; endothelial senescence; inflamm-ageing; longevity; miRNAs
Aguiar	Influence of Body Fat on Oxidative Stress and Telomere Length of Master Athletes	2021	antiaging; aging; exercise
Ahola	Work-related exhaustion and telomere length: a population-based study	2012	telomere length; telomeres; white blood cells; psychological stress; body mass idnex; smoking habits; diagnostic medicine; aging
Al-Daghri	Gut-derived endotoxin and telomere length attrition in adults with and without type 2 diabetes	2021	telomere length; endotoxin; inflammation; type 2 diabetes mellitus
Alonso-Pedrero	Dietary Exposure to Polychlorinated Biphenyls and Dioxins and Its Relationship to Telomere Length in Subjects Older Than 55 Years from the SUN Project	2022	polychlorinated biphenyls (PCBs); dioxins; telomere length (TL); cross-sectional study; Seguimiento Universidad de Navarra (SUN) study; nutritional epidemiology
Amaro-Gahete	Association of basal metabolic rate and fuel oxidation in basal conditions and during exercise, with plasma S-klotho: the FIT-AGEING study	2019	aging, MFO, basal fat oxidation, basal metabolic rate, Fat max
Amgalan	Brain age estimation reveals older adults' accelerated senescence after traumatic brain injury	2022	magnetic resonance imaging; neurodegeneration; machine learning; biological age; chronological age
An	Body weight status and telomere length in U.S. middle-aged and older adults	2017	Body weight statusObesityAdiposityTelomereOlder adult
Arts	Leucocyte telomere length is no molecular marker of physical frailty in late-life depression	2018	Telomere length; Frailty; Depressive disorder; Aged
Bae	A model for estimating body shape biological age based on clinical parameters associated with body composition	2013	chronological age; body shape; biological age
Bahour	Diabetes mellitus correlates with increased biological age as indicated by clinical biomarkers	2022	biological age; chronological age; diabetes
Banszerus	Exploring the Relationship of Relative Telomere Length and the Epigenetic Clock in the LipidCardio Cohort	2019	biomarker of ageing; biological age; aging; telomere length; epigenetic clock; DNA methylation (DNAm) age; DNAm age acceleration; LipidCardio Study; Berlin Aging Study II (BASE-II)
Barha	Number of Children and Telomere Length in Women: A Prospective, Longitudinal Evaluation	2016	telomere length; telomeres; cell aging; children; estradiol; bioenergetics; saliva; oxidative stress
Barha	Child mortality, hypothalamic-pituitary-adrenal axis activity and cellular aging in mothers	2017	telomere length; cortisol; child health; cell aging; telomeres; aging; maternal mortality; psychological stress
Bauer	Age, aging and physiological dysregulation in safety-critical work: a retrospective longitudinal study of helicopter emergency medical services pilots	2020	functional decline; biological age; age 60 rule; workforce aging; occupational safety
Beam	Midlife Study of the Louisville Twins: Connecting Cognitive Development to Biological and Cognitive Aging	2020	Louisville Twin Study; developmental behavioral genetics; cognitive ability; aging
Belsky	Quantification of biological aging in young adults	2015	biological sciences; medical sciences; social science; psychological and cognitive sciences
Belsky	Impact of early personal-history characteristics on the Pace of Aging: implications for clinical trials of therapies to slow aging and extend healthspan	2017	--
Belsky	Change in the Rate of Biological Aging in Response to Caloric Restriction: CALERIE Biobank Analysis	2018	Caloric restriction, Biological age, Geroscience, Geroprotector
Belsky	Eleven Telomere, Epigenetic Clock, and Biomarker-Composite Quantifications of Biological Aging: Do They Measure the Same Thing?	2018	biological aging, epigenetic clock, geroscience, telomere
Belsky	Quantification of the pace of biological aging in humans through a blood test, the DunedinPoAm DNA methylation algorithm	2020	--
Berben	Blood Immunosenescence Signatures Reflecting Age, Frailty and Tumor Immune Infiltrate in Patients with Early Luminal Breast Cancer	2021	breast cancer; blood biomarkers; tumor immune infiltrate; aging; frailty
Bobrov	PhotoAgeClock: deep learning algorithms for development of non-invasive visual biomarkers of aging	2018	photographic aging clock, photographic aging biomarker, age prediction, biomedical imaging, computer vision, deep learning
Borghini	Chronic and acute effects of endurance training on telomere length	2015	--
Bostrom	HPA-axis dysregulation is not associated with accelerated epigenetic aging in patients with hypersexual disorder	2022	Biological aging; Hypersexual disorder; HPA-axis; Dexamethasone suppression test; Compulsive sexual behavior disorder; DNA methylation clock
Bountziouka	Association of shorter leucocyte telomere length with risk of frailty	2022	--
Bourassa	Intimate partner violence and lower relationship quality are associated with faster biological aging	2020	biological aging, romantic relationships, intimate partner violence, relationship quality
Bourassa	Association of Treatable Health Conditions During Adolescence With Accelerated Aging at Midlife	2022	--
Brouwers	Biological ageing and frailty markers in breast cancer patients	2015	biomarkers, breast cancer, geriatric assessment, interleukin-6, frailty
Brouwers	The impact of adjuvant chemotherapy in older breast cancer patients on clinical and biological aging parameters	2016	breast cancer, older patients, adjuvant chemotherapy, biological aging, aging biomarkers
Brunt	Circulating interleukin-37 declines with aging in healthy humans: relations to healthspan indicators and IL37 gene SNPs	2023	IL37; single-nucleotide polymorphism; anti-inflammatory; cytokines; immunosenescence
Cabrera-Mendoza	Within subject cross-tissue analyzes of epigenetic clocks in substance use disorder postmortem brain and blood	2023	--
Cao	Modelling biological age based on plasma peptides in Han Chinese adults	2020	ageing, multiple linear regression, biological age, plasma peptide, primary prevention
Cao	Weight change across adulthood and accelerated biological aging in middle-aged and older adults	2023	biological agingbody mass indexbody weight changesobesityphenotypic age acceleration
Carroll	Socioeconomic factors and leukocyte telomere length in a multi-ethnic sample: Findings from the multi-ethnic study of atherosclerosis (MESA)	2013	Telomere length; Childhood SES; Socioeconomic status; Home ownership; Wealth; Parental education; Cellular aging; Biological aging; Ethnicity
Carroll	Epigenetic Aging and Immune Senescence in Women With Insomnia Symptoms: Findings From the Women's Health Initiative Study	2017	Aging; Epigenetic; Immunosenescence; Insomnia; Methylation; Sleep
Carroll	Cognitive performance in survivors of breast cancer and markers of biological aging	2019	--
Carroll	Obstructive sleep apnea, nighttime arousals, and leukocyte telomere length: the Multi-Ethnic Study of Atherosclerosis	2019	biomarkers, aging, obstructive sleep apnea, psychoneuroimmunology, actigraphy, epidemiology, telomere length, sleep
Carroll	Lifetime discrimination in low to middle income mothers and cellular aging: A prospective analysis	2022	Discrimination; Biological aging; Telomere length; Maternal health; Mothers; Women
Carulli	Telomere length elongation after weight loss intervention in obese adults	2016	Obesity; Weight loss; Bioenteric intragastric balloon; Telomere; Telomere length
Chae	Discrimination, mental health, and leukocyte telomere length among African American men	2016	African American men; Leukocyte telomere length; Racial discrimination; Depression; Anxiety
Chen	Modeling biological age using blood biomarkers and physical measurements in Chinese adults	2023	Biological age; blood biomarkers; physical measurements; mortality; cardiovascular health
Cheng	Shortened Relative Leukocyte Telomere Length Is Associated With Prevalent and Incident Cardiovascular Complications in Type 2 Diabetes: Analysis From the Hong Kong Diabetes Register	2020	--
Chmelar	Associations Between Age, Psychosocial Work Conditions, Occupational Well-Being, and Telomere Length in Geriatric Care Professionals	2017	--
Christensen	Idiopathic early ovarian aging: is there a relation with premenopausal accelerated biological aging in young women with diminished response to ART?	2021	early ovarian aging; telomere; accelerated aging; epigenetics
Cole	Brain age predicts mortality	2018	genetics; neuroscience; predictive markers
Contini	Salivary Proteomics Reveals Significant Changes in Relation to Alzheimer's Disease and Aging	2022	--
Couoh	Differences between biological and chronological age-at-death in human skeletal remains: A change of perspective	2017	--
Crimmins	Social hallmarks of aging: Suggestions for geroscience research	2020	Hallmarks of agingBiological age – chronological ageSocial determinants of aging
Crimmins	Quest for a summary measure of biological age: the health and retirement study	2021	biological age; phenotypic age; TAME markers; biomarkers
Crowe	Associations of Loneliness and Social Isolation With Health Span and Life Span in the U.S. Health and Retirement Study	2021	Biological aging, Chronic disease, Disability, Healthy aging
D'Aquila	Methylation of the ribosomal RNA gene promoter is associated with aging and age-related decline	2017	--
D'Aquila	Epigenetic signature: implications for mitochondrial quality control in human aging	2019	methylation, aging biomarkers, epigenetics biomarkers, mitochondrial quality control, geriatric parameters, chronological age, biological, age
De Francesco	Do people living with HIV experience greater age advancement than their HIV-negative counterparts?	2019	accelerated aging; aging; biological age; biomarkers of aging; HIV; premature aging
Denham	Longer leukocyte telomeres are associated with ultra-endurance exercise independent of cardiovascular risk factors	2013	telomeres; white blood cells; cardiovascular disease risk; exercise; aerobic exercise; C-reacive proteins; cholesterol; inflammation
Denham	Increased expression of telomere-regulating genes in endurance athletes with long leukocyte telomeres	2016	--
Dhillion	Shorter Telomere Length in Carriers of APOE-ε4 and High Plasma Concentration of Glucose, Glyoxal and Other Advanced Glycation End Products (AGEs)	2020	Advanced glycation end products, Telomere, APOE-ε4
Dmitrieva	Middle-age high normal serum sodium as a risk factor for accelerated biological aging, chronic diseases, and premature mortality	2023	serum sodium; hydration; aging; chronic diseases; biological age
Dolcini	Mitochondria and aging in older individuals: an analysis of DNA methylation age metrics, leukocyte telomere length, and mitochondrial DNA copy number in the VA normative aging study	2020	epigenetic, aging, mitochondria, telomere length, public health
Donze	Evidence for Accelerated Biological Aging in Young Adults with Prader–Willi Syndrome	2020	Prader–Willi syndrome, telomere length, growth hormone
Dugué	Association of DNA Methylation-Based Biological Age With Health Risk Factors and Overall and Cause-Specific Mortality	2022	age acceleration, aging, biological age, cancer, DNA methylation, epigenetic clock, health risk factors, mortality
Dunston	Terminal galactose residues on transferrin are increased in midlife adults compared to young adults	2012	--
Earls	Multi-Omic Biological Age Estimation and Its Correlation With Wellness and Disease Phenotypes: A Longitudinal Study of 3,558 Individuals	2019	Deep phenotyping, Precision medicine, Healthy aging, Biological age, Chronological age
Epel	Wandering minds and aging cells	2013	--
Erdmann	Mammographic density, blood telomere length and lipid peroxidation	2017	cancer epidemiology; risk factors
Esposito	Dietary Polyphenol Intake Is Associated with Biological Aging, a Novel Predictor of Cardiovascular Disease: Cross-Sectional Findings from the Moli-Sani Study	2021	biological aging; dietary polyphenols; cardiovascular disease; bone health
Esposito	Mediterranean diet and other dietary patterns in association with biological aging in the Moli-sani Study cohort	2022	Biological agingMediterranean dietDash dietPalaeolithic dietNordic dietPolyphenols
Fang	Association between elder abuse and telomere shortening in older adults: A 2-year prospective study	2021	--
Farina	Racial/Ethnic Differences in Biological Aging and Their Life Course Socioeconomic Determinants: The 2016 Health and Retirement Study	2023	--
Fedintsev	Markers of arterial health could serve as accurate non-invasive predictors of human biological and chronological age	2017	biological age, stenosis, pulse wave velocity, augmentation index, artery age
Finkel	Genetic and Environmental Influences on Longitudinal Trajectories of Functional Biological Age: Comparisons Across Gender	2017	functional bioage; chronological age; twins
Forrester	Navigating Black Aging: The Biological Consequences of Stress and Depression	2022	Biological age acceleration, CARDIA, Depression symptoms, High-effort coping, Social support
Forrester	Racial differences in the association of accelerated aging with future cardiovascular events and all-cause mortality: the coronary artery risk development in young adults study, 2007–2018	2022	health disparities; accelerated aging; biological aging; cardiovascular disease
Fraszczyk	The effects of bariatric surgery on clinical profile, DNA methylation, and ageing in severely obese patients	2020	obesity; bariatric surgery; morbid obesity; DNA methylation; epigenetics; epigenetic clock; biological age; EWAS
Galkin	Psychological factors substantially contribute to biological aging: evidence from the aging rate in Chinese older adults	2022	psychological aging, lifespan psychology, aging clocks, longevity
Gao	Comparative validation of an epigenetic mortality risk score with three aging biomarkers for predicting mortality risks among older adult males	2019	Mortality risk score, DNA methylation, biological aging, mortality, epigenetic epidemiology
Gao	Role of sleep quality in the acceleration of biological aging and its potential for preventive interaction on air pollution insults: Findings from the UK Biobank cohort	2022	--
Garagnani	Methylation of ELOVL2 gene as a new epigenetic marker of age	2012	--
Gaydosh	Testing Proposed Quantifications of Biological Aging in Taiwanese Older Adults	2020	Biomarkers, Physical performance, Cognitive decline, Mortality
Geronimus	Race-Ethnicity, Poverty, Urban Stressors, and Telomere Length in a Detroit Community-based Sample	2015	--
Gialluisi	Exploring domains, clinical implications and environmental associations of a deep learning marker of biological ageing	2022	biological ageing; deep neural networks; blood markers; mortality; hospitalizations; quality of life; lifestyles; socioeconomic status
Glei	Shorter Ends, Faster End? Leukocyte Telomere Length and Mortality Among Older Taiwanese	2015	Mortality, Telomeres, Biological aging, Taiwan, Inflammation
Golab	Biological aging and physical fitness in men aged 20–70 years from Kraków, Poland	2016	--
Gong	The Relationship between Dietary Copper intake and Telomere Length in Hypertension	2022	--

Primary Author	Title	Publication Year	Keywords
Gorelik	Effect of Daily Rhythms of Cortisol Secretion on the Rate of Aging in Men	2022	Aging markers, Biological age, Cortisol, Premature aging
Graf	Testing Black-White Disparities in Biological Aging Among Older Adults in the United States: Analysis of DNA-Methylation and Blood-Chemistry Methods	2022	aging, aging clocks, biological aging, healthy aging, pace of aging, racial disparities
Habib	Association between shorter leukocyte telomeres and multiple sclerosis	2020	Autoimmune diseases; Multiple sclerosis; Genetics; Telomere lengths; Relapsing remitting; Primary progressive; Secondary progressive
Hammadah	Telomere Shortening, Regenerative Capacity, and Cardiovascular Outcomes	2017	--
Han	Biological Age Is Associated with the Active Use of Nutrition Data	2018	biological age; nutrition facts; health information; patient awareness
Han	Is Sleep Duration Associated with Biological Age (BA)?: Analysis of (2010–2015) South Korean NHANES Dataset South Korea	2018	biological age; sleep duration; self-management; metabolism
Hassett	Pain is associated with short leukocyte telomere length in women with fibromyalgia	2012	Pain; fibromyalgia; telomere; premature aging; neuroimaging
Hastings	Comparability of biological aging measures in the National Health and Nutrition Examination Study, 1999–2002	2019	Aging; Biological aging; Telomeres; NHANES; Geroscience
Hastings	Conceptual and Analytical Overlap Between Allostatic Load and Systemic Biological Aging Measures: Analyses From the National Survey of Midlife Development in the United States	2022	Biomarkers, Epidemiology, Functional performance, Healthspan, Risk factors
Hautekiet	A healthy lifestyle is positively associated with mental health and well-being and core markers in ageing	2022	mental health; lifestyle; biological ageing; mitochondrial DNA content; telomere length
Hernando	The effect of long-term ultra-endurance exercise and SOD2 genotype on telomere shortening with age	2020	--
Hertel	Measuring Biological Age via Metabonomics: The Metabolic Age Score	2016	metabonomics; nonlinear regression techniques; aging; SHIP; mortality; NMR; multimorbidity
Ho	Effect of Diet, Exercise, or Both on Biological Age and Healthy Aging in Older Adults with Obesity: Secondary Analysis of a Randomized Controlled Trial	2022	Biological age; lifestyle; diet; exercise; older adults with obesity
Hoen	Association between anxiety but not depressive disorders and leukocyte telomere length after 2 years of follow-up in a population-based sample	2013	anxiety disorders; depressive disorders; longitudinal; telomere length
Holly	Towards a gene expression biomarker set for human biological age	2013	--
Hong	Profiles of histidine-rich glycoprotein associate with age and risk of all-cause mortality	2020	aging; methods and resources
Honig	Association of shorter leukocyte telomere repeat length with dementia and mortality	2012	--
Honig	Heritability of telomere length in a study of long-lived families	2015	Telomere; Heritability; Longevity; Aging
Honkonen	Leukocyte telomere length is inversely associated with arterial wave reflection in 566 normotensive and never-treated hypertensive subjects	2020	aging, circulatory system, hemodynamics, telomeres
Hovatta	Leukocyte Telomere Length in the Finnish Diabetes Prevention Study	2012	telomere length; white blood cells; telomeres; insulin; type 2 dibetes; diet; behavioral and social aspects of health; insulin secretion
Huang	Dynamics of leukocyte telomere length in adults aged 50 and older: a longitudinal population-based cohort study	2021	leukocyte telomere length; age; sex; longitudinal study; within-subject effect; between-subject effect
Iannarelli	No Mediation Effect of Telomere Length or Mitochondrial DNA Copy Number on the Association Between Adverse Childhood Experiences (ACEs) and Central Arterial Stiffness	2022	--
Jee	Development and Application of Biological Age Prediction Models with Physical Fitness and Physiological Components in Korean Adults	2012	Biological age, Chronological age, Physiological age, Physical fitness, Principal component analysis
Jee	Selection of an optimal set of biomarkers and comparative analyses of biological age estimation models in Korean females	2017	Chronological age; Biological age; Multiple linear regression; Principle component analysis
Johnson	The plasma metabolome as a predictor of biological aging in humans	2019	Biological aging, Metabolomics, Healthspan, Precision medicine
Kajantie	No association between body size at birth and leucocyte telomere length in adult life--evidence from three cohort studies	2012	telomere length, birthweight, very low birthweight, preterm birth, prenatal, ageing
Kang	Models for estimating the metabolic syndrome biological age as the new index for evaluation and management of metabolic syndrome	2017	metabolic syndrome; biological age; biomarker; index; health care
Kang	Biological age as a health index for mortality and major age-related disease incidence in Koreans: National Health Insurance Service – Health screening 11-year follow-up study	2018	health; aging; biological age; comprehensive index
Kankaanpaa 201	Do Epigenetic Clocks Provide Explanations for Sex Differences in Life Span? A Cross-Sectional Twin Study	2022	Biological age, DNA methylation, Life span, Lifestyle, Sex gap
Karametos	Chronic Obstructive Pulmonary Disease as a Main Factor of Premature Aging	2019	chronic obstructive pulmonary disease; aging; biological marker; dehydroepiandrosterone; DHEA; growth hormone; GH
Katz	Association of leukocyte telomere length with perceived physical fatigability	2022	Telomeres; Fatigue; Biological age; Family study
Kemp	Are Biological Consequences of Childhood Exposures Detectable in Telomere Length Decades Later?	2021	Telomere length, Biological age, Adverse childhood experiences
Khan	A null mutation in SERPINE1 protects against biological aging in humans	2017	--
Kim	The frailty index outperforms DNA methylation age and its derivatives as an indicator of biological age	2017	Aging; Biological Age; Frailty; DNA methylation; Mortality
Kim	DNA methylation associated with healthy aging of elderly twins	2018	DNA methylation; frailty index; biological age; protocadherin; aging
Kingma	The association between intelligence and telomere length: a longitudinal population based study	2012	Telomere length; intelligence; educational attainment; intelligence tests; socioeconomic aspects of health; white blood cells; smoking habits; telomeres
Kocaman	The degree of premature hair graying as an independent risk marker for coronary artery disease: a predictor of biological age rather than chronological age	2012	Hair graying; Male gender; Coronary artery disease; Computed tomography coronary angiography
Koh	Left Atrial Phasic Function in Older Adults Is Associated with Fibrotic and Low-Grade Inflammatory Pathways	2023	Cardiovascular, Left atrium, Mechanisms, Biomarkers, Aging
Kolokoltsev	The effect of physical activity on biological age and body composition in 18–19-year-old girls	2022	--
Kristic	Glycans are a novel biomarker of chronological and biological ages	2014	Aging, Glycome, Glycosylation, Immunoglobulin G, Inflammation.
Kuffer	Posttraumatic stress disorder, adverse childhood events, and buccal cell telomere length in elderly {Swiss} former indentured child laborers	2016	--
Kuo	A roadmap to build a phenotypic metric of ageing: insights from the Baltimore Longitudinal Study of Aging	2020	--
Kuzawa	Birth weight and maternal energy status during pregnancy as predictors of epigenetic age acceleration in young adults from metropolitan Cebu, Philippines	2022	DOHaD; ageing; pregnancy; epigenetic clocks; senescence
Langevin	Life-course persistent antisocial behavior and accelerated biological aging in a longitudinal birth cohort	2022	antisocial trajectories; biological aging; crime; accelerated aging
Le Nguyen	Loving-kindness meditation slows biological aging in novices: Evidence from a 12-week randomized controlled trial	2019	Contemplative science; Metta; Compassion meditation; Cell aging; Positive psychology
Lee	Inverse association between adiposity and telomere length: The fels longitudinal study	2011	--
Lee	Association between dietary patterns in the remote past and telomere length	2015	--
Lee	Longitudinal associations between micronutrient consumption and leukocyte telomere length	2017	--
Lee	The link between discrimination and telomere length in African American adults	2017	--
Lee	Relationship between telomere shortening and age in Korean individuals with mild cognitive impairment and Alzheimer's disease compared to that in healthy controls	2020	aging, telomere length, mild cognitive impairment, Alzheimer's disease, positron emission tomography
Levine	Modeling the rate of senescence: can estimated biological age predict mortality more accurately than chronological age	2013	Biological age, Biomarkers, Mortality.
Levine	Evidence of accelerated aging among African Americans and its implications for mortality	2014	Aging; Racial disparities; Biomarkers; Mortality selection; Life expectancy
Levine	Is 60 the New 50? Examining Changes in Biological Age Over the Past Two Decades	2018	Biomarkers, Aging, Time trends, Obesity, Smoking
Li	Longitudinal trajectories, correlations and mortality associations of nine biological ages across 20-years follow-up	2020	--
Li	Biological age models based on a healthy Han Chinese population	2023	Biological age; Chronological age; Aging markers; Age; Healthy; Human
Lin	Transcriptome-wide association study of inflammatory biologic age	2017	inflammation, gene expression, aging, epidemiology
Lin	Whole Blood Gene Expression Associated With Clinical Biological Age	2019	Gene expression, Biologic aging, Epidemiology
Lin	Shortened leukocyte telomere length in young adults who use methamphetamine	2021	Addiction; physiology; prognostic markers
Lincoln	Social Relationships and Salivary Telomere Length Among Middle-Aged and Older African American and White Adults	2019	African Americans, Negative interaction, Social support, Telomere length
Liu	Association between Body Iron Status and Leukocyte Telomere Length, a Biomarker of Biological Aging, in a Nationally Representative Sample of US Adults	2019	Adults; Aging; Ferritin; Iron; Telomere length
Liu	Development and Validation of 2 Composite Aging Measures Using Routine Clinical Biomarkers in the Chinese Population: Analyses From 2 Prospective Cohort Studies	2021	Aging measure, Chinese population, Klemera and Doubal method, Mortality, Physiological dysregulation
Lu	Association between exposure to second-hand smoke and telomere length: cross-sectional study of 1303 non-smokers	2017	Telomere length, second-hand smoke, biological ageing
Lu	Plasma inflammation-related biomarkers are associated with intrinsic capacity in community-dwelling older adults	2023	--
Lucas	Justice for all? Beliefs about justice for self and others and telomere length in African Americans.	2018	--
Madrigano	Aging and epigenetics: Longitudinal changes in gene-specific DNA methylation	2012	aging; DNA methylation; epigenesis; genetic
Maeda	The correlation between the clinical laboratory data and the telomere length in peripheral blood leukocytes of Japanese female patients with hypertension	2011	telomere length; subtelomeric methylation; blood sugar level; renal function; arteriosclerosis
Maeda	Epigenetic status of subtelomere of peripheral leukocytes corresponds to cardiographic parameters with a sex association	2018	--
Maeda	The approximate formulas predicting personal somatic telomere length using patient blood test data	2019	--
Maeda	Chromosomal terminal methylation status is associated with gut microbiotic alterations	2021	Aging; telomere; genomic methylation; gut microbiota; postmenopause
Maeda	Telomere shortening velocity of patients administered with hypnotics is accelerated in a gender-differential manner	2021	--
Mamoshina	Population Specific Biomarkers of Human Aging: A Big Data Study Using South Korean, Canadian, and Eastern European Patient Populations	2018	Biochemistry aging clocks, Biological age, Deep Learning, Deep Neural Networks, Machine Learning
Mamoshina	Blood Biochemistry Analysis to Detect Smoking Status and Quantify Accelerated Aging in Smokers	2019	Computational platforms and environments, data processing, health policy, molecular medicine
Marioni	Association of facial ageing with DNA methylation and epigenetic age predictions	2018	DNA methylation; facial ageing, epigenetic clock, surivival
Marriott	Serum testosterone and sex hormone–binding globulin are inversely associated with leucocyte telomere length in men: a cross-sectional analysis of the UK Biobank study	2023	testosterone, sex hormone–binding globulin, leucocyte telomere length, male ageing
Martens	Retinal microcirculation and leukocyte telomere length in the general population	2018	biomarkers; cardiology
Martinez	Association between the Inflammatory Potential of the Diet and Biological Aging: A Cross-Sectional Analysis of 4510 Adults from the Moli-Sani Study Cohort	2023	aging; biological age; inflammation; inflammatory diet
Martinez de Toda	The Immunity Clock	2021	Aging, Biological age, Immune system, Prediction
Mason	Childhood Physical and Sexual Abuse History and Leukocyte Telomere Length among Women in Middle Adulthood	2015	Child abuse; telomere length; telomeres; drug abuse; blood; child health; type 2 diabetes; professions
Mastrobattista	Late-Life Depression is Associated With Increased Levels of GDF-15, a Pro-Aging Mitokine	2023	Late life depressionbiological markersGDF-15AgingGeroscience
Mate	Accelerated immunosenescence, oxidation and inflammation lead to a higher biological age in COPD patients	2021	Oxidative stress; Inflammatory stress; Rate of aging
Maxwell	Telomere Attrition and Decreased Fetuin-A Levels Indicate Accelerated Biological Aging and Are Implicated in the Pathogenesis of Colorectal Cancer	2011	--
McClelland	Accelerated ageing and renal dysfunction links lower socioeconomic status and dietary phosphate intake	2016	phosphate, diet, poverty, ageing, CKD, pSoBid
McCrory	Epigenetic Clocks and Allostatic Load Reveal Potential Sex-Specific Drivers of Biological Aging	2020	Biology of aging, Biomarkers, DNA methylation
McLachlan	Attitudes to ageing, biomarkers of ageing and mortality: the Lothian Birth Cohort 1936	2020	--

Primary Author	Title	Publication Year	Keywords
Mehta	Telomere length is associated with HIV infection, methamphetamine use, inflammation, and comorbid disease risk	2021	HIV; Methamphetamine; Telomeres; Aging; Cardiovascular
Meier	Cellular aging over 13 years associated with incident antinuclear antibody positivity in the Baltimore Longitudinal Study of Aging	2019	Autoimmunity; Telomere length; Antinuclear antibodies; Epidemiology; Immune aging
Meisel	Construction of a Biological Age Score to Predict Tooth Loss over 10 Years	2019	--
Meng	Using Inflammatory Biological Age To Evaluate the Preventing Aging Effect of a Polyphenol-Probiotic-Enhanced Dietary Pattern in Adults Aged 50 Years and Older	2023	Polyphenol-probiotic-enhanced dietary pattern; gut microbiota; intestinal metabolite; inflammatory biology age; metabolome
Meyer	Leukocyte telomere length is related to appendicular lean mass: cross-sectional data from the Berlin Aging Study II (BASE-II)	2016	appendicular lean massBASE-IIleukocytesmuscle masssarcopeniatelomere length
Minami	Correlates of telomere length shortening in peripheral leukocytes of HIV-infected individuals and association with leukoaraiosis	2019	Telomere length; HIV; HIV infections; virl load; magnetic resonance imaging; inflammation; regression analysis; white blood cells
Mitnitski	Heterogeneity of Human Aging and Its Assessment	2017	Biological aging, Biomarkers, Frailty indices, Biological age, Health heterogeneity
Mongelli	Evidence for Biological Age Acceleration and Telomere Shortening in COVID-19 Survivors	2021	biological age; COVID-19; post-COVID-19; telomeres; epigenetics; DNA methylation; ACE2; DPP-4; DeltaAge
Moverare-Skrtic	Leukocyte Telomere Length (LTL) is reduced in stable mild cognitive impairment but low LTL is not associated with conversion to Alzheimer's Disease: A pilot study	2012	Leukocyte telomere length; Biological aging; Alzheimer's disease; Mild cognitive impairment; Cerebrospinal fluid
Mukli	Urinary Biomarkers of Oxidative Stress in Aging: Implications for Prediction of Accelerated Biological Age in Prospective Cohort Studies	2022	--
Muniesa	Telomere Length in Elite Athletes	2017	leukocytes; biological aging; DNA; high-level athletes; telomerase
Muscari	N-terminal pro B-type natriuretic peptide (NT-proBNP): a possible surrogate of biological age in the elderly people	2021	NT-proBNP; mortality; biological age; epidemiological studies
Needham	Do black/white differences in telomere length depend on socioeconomic status?	2019	--
Negasheva	Biological age and tempos of aging in women over 60 in connection with their morphofunctional characteristics	2014	biological age; tempos of aging; body build; body composition; elderly and long-lived women
Ng	Socio-Environmental, Lifestyle, Behavioural, and Psychological Determinants of Biological Ageing: The Singapore Longitudinal Ageing Study	2020	Health span, Promoting factors, Physical function, Mental and cognitive activity, Nutrition manuscript length
Normando	Association between socioeconomic markers and adult telomere length differs according to sex: Pro-Saúde study	2020	Telomere; Aging; Social determinants of health; Brazil
Nusinovici	Retinal photograph-based deep learning predicts biological age, and stratifies morbidity and mortality risk	2022	Deep learning, artificial intelligence, biological age, retinal photograph, mortality, cardiovascular disease, cancer, older people
Nwanaji-Enwerem	An epigenetic aging analysis of randomized metformin and weight loss interventions in overweight postmenopausal breast cancer survivors	2021	RCT; DNA methylation age; biomarkers; GrimAge; PhenoAge
O'Donovan	Childhood trauma associated with short leukocyte telomere length in posttraumatic stress disorder	2011	Anxiety; biological aging; childhood trauma; post-traumatic stress disorder; telomere length
O'Donovan	Cumulative Inflammatory Load Is Associated with Short Leukocyte Telomere Length in the Health, Aging and Body Composition Study	2011	Inflammation; inflammatory diseases; telomeres; C-reactive proteins; white blood cells; elderly; cytokines; telomere length
Obeid	Effect of adding B-vitamins to vitamin D and calcium supplementation on CpG methylation of epigenetic aging markers	2018	Aging; DNA-Methylation; Epigenetics; Supplementation; Vitamins
Ock	Organophosphate insecticide exposure and telomere length in U.S. adults	2019	Organophosphate insecticide; Telomere length; NHANES; Epidemiology; Aging
Okazaki	Epigenetic clock analysis and increased plasminogen activator inhibitor-1 in high-functioning autism spectrum disorder	2022	Autism spectrum disorder; epigenetics; blood plasma; DNA methylation; plasma proteins; telomere length; blood; methylation
Ormseth	Telomere Length and Coronary Atherosclerosis in Rheumatoid Arthritis	2016	Rheumatoid arthritis; telomere; atherosclerosis; cardiovascular; aging
Osthus	Telomere Length and Long-Term Endurance Exercise: Does Exercise Training Affect Biological Age? A Pilot Study	2012	Telomere length; exercise; telomeres; aging; oxygen; physical fitness; elderly; sports
Pan	Association of PM2.5 Components with Acceleration of Aging: Moderating Role of Sex Hormones	2023	KDM-biological age; PM2.5 components; BTH area; sex hormones; modification
Paparazzo	A blood-based molecular clock for biological age estimation	2022	sjTRECs; ELOVL2; age prediction; epigenetics
Pathai	Accelerated biological ageing in HIV-infected individuals in South Africa: a case–control study	2013	accelerated ageing; Africa; biomarkers of ageing; CDKN2A; HIV; telomeres
Pathai	Assessment of candidate ocular biomarkers of ageing in a South African adult population: relationship with chronological age and systemic biomarkers	2013	Telomeres; CDKN2A; Lens density; Retinal vessel calibre; Corneal endothelium; Retinal nerve fibre layer; Frailty
Pathai	Ocular parameters of biological ageing in HIV-infected individuals in South Africa: relationship with chronological age and systemic biomarkers of ageing	2013	Telomeres; CDKN2A; Lens density; Retinal vessel calibre; HIV
Pavanello	Exploring epigenetic age in response to intensive relaxing training: a pilot study to slow down biological age	2019	epigenetic age; DNA methylation age; relaxing training; telomere length; myocardial infarction patient; telomerase
Pearce	DNA-methylation-based telomere length estimator: comparisons with measurements from flow FISH and qPCR	2021	telomere length, qPCR, flow FISH, DNAmTL, agreement
Peixoto	Factors associated with early biological aging in older people with HIV	2023	Biological age; aging; HIV; artificial intelligence
Praveen	Relative telomere length and mitochondrial DNA copy number variation with age: association with plasma folate and vitamin B12	2020	Relative telomere length; Mitochondrial DNA copy number; Age; Folate; Vitamin B12
Pruszkowska-Przybylska	Evaluation of epigenetic age calculators between preeclampsia and normotensive pregnancies in an Australian cohort	2022	DNA; epigenetics; genetics research; hypertension
Pyrkov	Extracting biological age from biomedical data via deep learning: too much of a good thing?	2018	Ageing; biophysics; machine learning; population screening
Pyrkov	Quantitative characterization of biological age and frailty based on locomotor activity records	2018	biological clock, health span, NHANES, UK Biobank, physical activity
Raghu	Deep Learning to Estimate Biological Age From Chest Radiographs	2021	--
Rahman	Deep learning using convolutional LSTM estimates biological age from physical activity	2019	Biomarkers; biomedical engineering; epidemiology
Rahman	Centroid of age neighborhoods: A new approach to estimate biological age	2020	Biomarkers; estimation; blood pressure; correlation; informatics; blood
Rapp	Where are populations aging better? A global comparison of healthy aging across organization for economic cooperation and development countries	2022	health capital depreciation; healthy aging; physiological age
Raschenberger	The association of relative telomere length with symptomatic peripheral arterial disease: results from the CAVASIC study	2013	Peripheral arterial disease; Cardiovascular disease; Telomere length; Aging; Atherosclerosis; Chromosomal stability
Rasmussen	Association between elevated suPAR, a new biomarker of inflammation, and accelerated aging	2021	Gait speed, Immunosenescence, Inflammaging, MRI, Pace of aging
Rehkopf	Longer leukocyte telomere length in Costa Rica's Nicoya Peninsula: a population-based study	2013	Telomere length; Costa Rica; Aging; Biomarkers; Socioeconomic; Longevity
Rej	Shortened telomere length is associated with unfair treatment attributed to race in African Americans living in Tallahassee, Florida	2020	--
Rentscher	Chronic stress exposure and daily stress appraisals relate to biological aging marker p16INK4a	2019	Stress; Daily diary; Biological aging; Cellular senescence; Telomere length; Gene expression
Rentscher	Relationship closeness buffers the effects of perceived stress on transcriptomic indicators of cellular stress and biological aging marker p16INK4a	2020	psychological stress, relationship closeness, biological aging, cellular senescence, gene expression
Revesz	Dysregulated physiological stress systems and accelerated cellular aging	2014	Cellular aging; Telomeres; Stress; Aging; Inflammation; Autonomic nervous system; Cortisol
Revesz	Associations between cellular aging markers and metabolic syndrome: findings from the CARDIA study	2017	--
Roberts	Telomere length and the risk of atrial fibrillation: insights into the role of biological versus chronological aging	2014	--
Robertson	Is telomere length socially patterned? Evidence from the West of Scotland Twenty-07 Study	2012	Telomere length; telomeres; socioeconomic aspects of health; employment; social stratification; time measurememnt; aging; finance
Robinson	Determinants of accelerated metabolomic and epigenetic aging in a UK cohort	2020	--
Ross	Immune epigenetic age in pregnancy and 1 year after birth: associations with weight change	2020	--
Russo	Telomere length variation in juvenile acute myocardial infarction	2012	Coronary heart disease; telomere length; white blood cells; polymerase chain reaction; telomeres; cardiovascular disease risk; hypercholesterolemia; myocardial infarction
Ryan	Telomere length in depression and association with therapeutic response to electroconvulsive therapy and cognitive side-effects	2020	Cognitive outcomes; depression; electroconvulsive therapy; side-effect; telomere
Safaee	Association of telomere length with risk of complications in adult spinal deformity surgery: a pilot study of 43 patients	2023	telomere; cellular age; chronological age; complications; deformity surgery
Salignon	Beyond chronological age: a multidimensional approach to survival prediction in older adults	2023	Biological age, Chronological age, Multidimensional assessment, Personalized medicine, Survival
Samuel	Socioeconomic status, race/ethnicity, and diurnal cortisol trajectories in middle-aged and older adults	2018	Earlier aging, Education, Hypothalamic pituitary adrenal axis, Wealth
Sanchis	Growth differentiation factor 15 and geriatric conditions in acute coronary syndrome	2019	Growth differentiation factor 15ElderlyAcute coronary syndrome
Savolainen	Associations between early life stress, self-reported traumatic experiences across the lifespan and leukocyte telomere length in elderly adults	2014	Telomere length; Early life stress; Traumatic experiences; Cellular aging; Biomarker; Aging-related diseases
Sawhney	Telomere shortening and telomerase activity in ischaemic cardiomyopathy patients–Potential markers of ventricular arrhythmia	2016	Ventricular arrhythmia; Telomere biology; Implantable cardioverter defibrillator
Schafer	The senescence-associated secretome as an indicator of age and medical risk	2020	Aging
Schrempft	Associations between life-course socioeconomic conditions and the pace of aging	2022	Biological age, Biomarkers, Physiological decline, Social inequalities, Social mobility
Schumann	Using machine learning to estimate the calendar age based on autonomic cardiovascular function	2023	aging, heart rate variability, blood pressure variability, baroreflex, pulse pressure
Seki	No strong association among epigenetic modifications by DNA methylation, telomere length, and physical fitness in biological aging	2023	Telomere; exercise; physical fitness; DNA methylation; epigenetical aging
Senior	Multidimensional associations between nutrient intake and healthy ageing in humans	2022	Ageing; dysregulation; geometric framework; healthspan; nutrition; systems epidemiology
Shalev	Internalizing disorders and leukocyte telomere erosion: a prospective study of depression, generalized anxiety disorder and post-traumatic stress disorder	2014	Depression; generalized anxiety disorder; internalizing disorders; longitudinal; post-traumatic stress disorder; telomere length
Shapiro	Familial aggregation of the aging process: biological age measured in young adult offspring as a predictor of parental mortality	2023	Cohort study; biological age; aging; mortality; familial aggregation; intergenerational associations
Shiels	Accelerated telomere attrition is associated with relative household income, diet and inflammation in the pSoBid cohort	2011	Telomere length; telomeres; diet; cholesterol; socioeconomic aspects of health; biomarkers; social stratification; housing
Shin	Association between snoring and leukocyte telomere length	2016	population-based study, leukocyte telomere length, polysomnography, snoring
Shin	Sex-Specific Association between Longitudinal Changes in Adiposity, FTO rs9939609 Polymorphism, and Leukocyte Telomere Length	2016	leukocyte telomere length; biological aging; adiposity; FTO polymorphism; longitudinal study
Shin	Plasma proteomic signature of cellular senescence and markers of biological aging among postmenopausal women	2022	--
Shirazi	Parity predicts biological age acceleration in post-menopausal, but not pre-menopausal, women	2020	Evolutionary theory; reproductive biology; senescence
Shokr	The Use of Retinal Microvascular Function and Telomere Length in Age and Blood Pressure Prediction in Individuals with Low Cardiovascular Risk	2022	biological age; telomeres; prediction; blood pressure; regression
Sillanpaa	Biological clocks and physical functioning in monozygotic female twins	2018	Epigenetic clock; telomeres; methylation; twin design; post-menopausal; physical function
Simons	Testing life course models whereby juvenile and adult adversity combine to influence speed of biological aging	2019	--
Smeets	Leukocyte Telomere Length in Young Adults Born Preterm: Support for Accelerated Biological Ageing	2015	Telomere length; cardiovascular disease; young adults; cholesterol; telomeres; birth weight; medical risk factors; preterm birth
Sol	Plasma acylcarnitines and gut-derived aromatic amino acids as sex-specific hub metabolites of the human aging metabolome	2023	--
Sood	A novel multi-tissue RNA diagnostic of healthy ageing relates to cognitive health status	2015	Healthy aging; cognitive health; general score; healthy elderly humans; Uppsala Longitudinal Study of Adult Men (ULSAM)
Srour	Serum markers of biological ageing provide long-term prediction of life expectancy—a longitudinal analysis in middle-aged and older German adults	2022	life expectancy, ageing biomarkers, lifestyle factors, biological ageing, prevention older people
St. Sauver	Inflammatory biomarkers, multi-morbidity, and biologic aging	2022	--
Sternang	Associations between functional biological age and cognition among older adults in rural Bangladesh: comparisons with chronological age	2019	--
Strandberg	Association of telomere length in older men with mortality and midlife body mass index and smoking	2011	Telomeres, Obesity, Smoking, Mortality
Strandberg	Association between alcohol consumption in healthy midlife and telomere length in older men. The Helsinki Businessmen Study	2012	Aged; alcohol; midlife; telomere

Primary Author	Title	Publication Year	Keywords
Strazhesko	Physiological health indexes predict deterioration and mortality in patients with COVID-19: a comparative study	2022	biological age, COVID-19, COVID-19 prognosis, SARS-CoV-2, COVID-19 outcome, pandemic
Sumida	Association between Nrf2 and CDKN2A expression in patients with end-stage renal disease: a pilot study	2020	aging, CDKN2A, end-stage renal disease, inflammation, Nrf2
Surtees	Educational attainment and mean leukocyte telomere length in women in the European Prospective Investigation into Cancer (EPIC)-Norfolk population study	2012	Education; Epidemiology; Psychological stress; Socioeconomic status; Telomere length
Suvarna	Metabolic and Cardiovascular Ageing Indices in Relation to Glycated Haemoglobin in Healthy and Diabetic Subjects	2017	Ageing; ageing index; glycosylated haemoglobin; heart rate variability; resting heart rate; type 2 diabetes mellitus
Tian	Association between oxidative stress and peripheral leukocyte telomere length in patients with premature coronary artery disease	2017	Coronary Artery Disease, Oxidative Stress, Telomere
Topiwala	Alcohol consumption and telomere length: Mendelian randomization clarifies alcohol's effects	2022	Genetics; prognostic markers
Toyoshima	Difference between “Physical Fitness Age” Based on Physical Function and Chronological Age Is Associated with Obesity, Hyperglycemia, Depressive Symptoms, and Low Serum Albumin	2022	Biological age; physical fitness age;physical function;sarcopenia; aging
Tucker	Alpha-and gamma-tocopherol and telomere length in 5768 US men and women: a NHANES study	2017	cell aging; antioxidant; vitamin E; DNA
Tucker	Dietary fiber and telomere length in 5674 US adults: an NHANES study of biological aging	2018	complex carbohydrate; ageing; lignin; whole grains; resistant starch; diet
Tucker	Milk fat intake and telomere length in US women and men: The role of the milk fat fraction	2019	--
Tucker	Fruit and vegetable intake and telomere length in a random sample of 5448 US adults	2021	telomere; diet; carbohydrate; NHANES; antioxidant; inflammation; legume; potato
Tucker	Insulin resistance and biological aging: the role of body mass, waist circumference, and inflammation	2022	--
Verhoeven	Major depressive disorder and accelerated cellular aging: results from a large psychiatric cohort study	2014	cell aging; depression; mood disorders; telomere shortening
Verschoor	Comparing biological age estimates using domain-specific measures from the Canadian Longitudinal Study on Aging	2021	Biological age, Biological measures, Biomarkers, CLSA, Healthy aging
Vetter	Epigenetic clock and relative telomere length represent largely different aspects of aging in the Berlin Aging Study II (BASE-II)	2019	DNA methylation age, Epigenetic clock, Telomere length, Berlin Aging Study II, BASE-II
Vetter	Relationship between 5 epigenetic clocks, telomere length, and functional capacity assessed in older adults: cross-sectional and longitudinal analyses	2022	Biological age, Epigenetic clock, Frailty, Longitudinal, Telomere length
Vincent	Assessing the contributions of childhood maltreatment subtypes and depression case-control status on telomere length reveals a specific role of physical neglect	2017	--
von Falkenhausen	Common electrocardiogram measures are not associated with telomere length	2022	electrocardiogram, telomere length, cardiac aging
von Kanel	Leukocyte telomere length and hemostatic factors in a South African cohort: the SABPA Study	2014	blood coagulation; cardiovascular disease; ethnicity;fibrinolysis; genetics
von Kanel	Comparison of telomere length in black and white teachers from South Africa: the sympathetic activity and ambulatory blood pressure in Africans study	2015	--
Vyas	Pilot study of DNA methylation, molecular aging markers and measures of health and well-being in aging	2019	human behaviour; predictive markers; scientific community
Vyas	Lifestyle and behavioral factors and mitochondrial DNA copy number in a diverse cohort of mid-life and older adults	2020	Smoking habits; physical activity; alcohol consumption; depression; mitochondrial DNA: aging; oxidative stress; Hispanic people
Waaijer	Morphometric skin characteristics dependent on chronological and biological age: the Leiden Longevity Study	2012	Skin ageing; chronological age; biological age; familial longevity; epidermis; elastic fibres
Waaijer	DNA damage markers in dermal fibroblasts in vitro reflect chronological donor age	2016	human aging, 53BP1, telomere-associated foci, micronuclei, biological age
Wagenmakers	BrainAge of patients with severe late-life depression referred for electroconvulsive therapy	2023	Late-life depressionBrainAge gapBiological ageElectroconvulsive therapy
Wan	Molecular markers of aging, exercise capacity, & physical activity in COPD	2021	Chronic obstructive pulmonary diseaseEpigenetic clockExercise tolerancePhysical activity
Wang	A machine learning–based biological aging prediction and its associations with healthy lifestyles: the Dongfeng–Tongji cohort	2022	biological aging; machine learning; mortality risk; healthy lifestyles; cohort study
Ward-Caviness	Long-term exposure to air pollution is associated with biological aging	2016	epigenetic aging, telomere length, biological aging, air pollution, black carbon, Gerotarget
Watkins	Hostility and telomere shortening among US military veterans: Results from the National Health and Resilience in Veterans Study	2016	Hostility; Aging; Telomere length; Veterans; Anger
Waziry	Quantification of biological age as a determinant of age-related diseases in the Rotterdam Study: a structural equation modeling approach	2019	Biological age; morbidity; mortality; biologically young; elderly
Weber	Learning on the job, the use of selection, optimization, and compensation strategies, and their association with telomere length as an indicator of biological aging	2019	Learning opportunities; selection, optimization, compensation; telomere length; resources; aging
Wikgren	Shorter telomere length is linked to brain atrophy and white matter hyperintensities	2014	brain atrophy, older people, telomere length, white matter hyperintensities
Wilson	Are macular drusen in midlife a marker of accelerated biological ageing?	2023	Ageing; biomarker; drusen
Wium-Andersen	Telomere length and depression: prospective cohort study and Mendelian randomisation study in 67 306 individuals	2017	--
Wiweko	Chronological age vs biological age: an age-related normogram for antral follicle count, FSH and anti-Mullerian hormone	2013	Normogram; anti-Mullerian hormone; antral follicle count; FSH
Wolkowitz	Leukocyte telomere length: effects of schizophrenia, age, and gender	2017	Schizophrenia; Telomere; Aging; Gender; Antipsychotics; Life style
Wynchank	Delayed sleep-onset and biological age: late sleep-onset is associated with shorter telomere length	2019	aging, leukocyte telomere length, delayed sleep phase, circadian rhythms, insomnia
Xia	Three-dimensional facial-image analysis to predict heterogeneity of the human ageing rate and the impact of lifestyle	2020	Ageing; machine learning
Xie	Dietary inflammatory potential and biological aging among US adults: a population-based study	2023	Dietary inflammatory index; aging; telomere length; NHANES; Klotho
Yang	A machine learning-based data mining in medical examination data: a biological features-based biological age prediction model	2022	Biological age; biological features; machine learning; interpolation; stacking; health status
Yeap	Epidemiological and Mendelian randomization studies of dihydrotestosterone and estradiol and leukocyte telomere length in men	2016	--
Yeap	Cross-sectional associations of sex hormones with leucocyte telomere length, a marker of biological age, in a community-based cohort of older men	2019	--
Yeap	Associations of plasma IGF1, IGFBP3 and estradiol with leucocyte telomere length, a marker of biological age, in men	2020	--
Yen	Older adults with higher income or marriage have longer telomeres	2013	telomere, income, marital status, neighbourhood, stress, older people
Yu	Telomere length is not associated with frailty in older Chinese elderly: Cross-sectional and longitudinal analysis	2015	Ageing; Frailty phenotype; Incident frailty; Pre-frailty; Telomere length
Yu	Profiling IgG N-glycans as potential biomarker of chronological and biological ages: A community-based study in a Han Chinese population	2016	biological age; biomarker; chronological age; immunoglobulin G; N-glycan
Yu	Differences between multimodal brain-age and chronological-age are linked to telomere shortening	2022	Brain-age; Cognitive-age; Telomere; Resting-state functional connectivity; Structural connectivity; Subcortical gray matter; Cortical thickness
Zelazniewicz	Perceived facial age and biochemical indicators of glycemia in adult men and women	2022	Ageing; human behaviour; predictive markers
Zhang	Select aging biomarkers based on telomere length and chronological age to build a biological age equation	2014	Telomere length; biological age; chronological age; aging markers
Zhang	The correlation between peripheral leukocyte telomere length and indicators of cardiovascular aging	2014	Telomere length; Cardiovascular function; Blood pressure; Electrocardiogram; Cardiovascular ultrasound
Zhang	Model construction for biological age based on a cross-sectional study of a healthy Chinese Han population	2017	Biological agechronological ageprincipal component analysis
Zhang	Phthalates and phenols, leukocyte telomere length, and breast cancer risk and mortality in the long island breast cancer study project	2022	--
Zhao	Depressive symptoms are associated with leukocyte telomere length in American Indians: findings from the Strong Heart Family Study	2016	American Indian, depression, leukocyte telomere length, Strong Heart Study
Zhao	Ultra-weak photon emission of hands in aging prediction	2016	Ultra-weak photon emission; Model prediction; Aging
Zhao	Land use mix and leukocyte telomere length in Mexican Americans	2021	Environmental sciences; environmental social sciences; risk factors
Zhong	Estimating biological age in the Singapore longitudinal aging study	2020	Aging, Physiological biomarkers, Biological age, Mortality, Frailty
Zhu	Human PBMC scRNA-seq–based aging clocks reveal ribosome to inflammation balance as a single-cell aging hallmark and super longevity	2023	--
